# Synthesis of an [(NHC)_2_Pd(SiMe_3_)_2_] Complex and Catalytic *cis*-Bis(silyl)ations of Alkynes with Unactivated Disilanes**

**DOI:** 10.1002/anie.201501764

**Published:** 2015-04-09

**Authors:** Melvyn B Ansell, Debbie E Roberts, F Geoffrey N Cloke, Oscar Navarro, John Spencer

**Affiliations:** Department of Chemistry, University of SussexBrighton BN1 9QJ (UK)

**Keywords:** alkynes, N-heterocyclic carbene, palladium, silicon, synthetic methods

## Abstract

The novel complex *cis*-[(ITMe)_2_Pd(SiMe_3_)_2_ (ITMe=1,3,4,5-tetramethylimidazol-2-ylidene) has been synthesized by mild oxidative cleavage of Me_3_SiSiMe_3_ using [(ITMe)_2_Pd^0^]. The use of this complex as precatalyst for the *cis*-bis(silyl)ation of alkynes using unactivated disilanes is reported.

The transition metal catalyzed activation of disilanes for the synthesis of high-value organosilicon compounds has received a significant amount of attention from industry and academia. Applications encompass the formation of silanes,[1] bis(silyl)ation of unsaturated compounds,[2] aryl/acyl silane synthesis,[3,4] and protection of alcohols.[5] Since the oxidative cleavage of the Si—Si bond by a low-valent platinum-group transition-metal center is proposed as a vital step for some of these processes,[6] the isolation of the resulting bis(silyl) transition-metal complexes is of great interest for elucidating reaction mechanisms. Unfortunately, the synthesis of such complexes has been largely limited to the oxidative addition of strained or activated disilanes.[7]

The cleavage of non-activated hexamethyldisilane is particularly challenging. Examples of the resulting bis(trimethylsilyl) platinum-group metal complexes are rare: only two have been described in the literature, and they bear either phosphine or isocyanide ligands. Braun and co-workers reported that [Pt(PEt_3_)_3_] reacted with a large excess of hexamethyldisilane to yield *cis*-[Pt(PEt_3_)_2_(SiMe_3_)_2_] at ambient temperature, but it only went to 50 % completion after three weeks.[8] Earlier, Ito and co-workers synthesized *cis*-[Pt(CNAd)_2_(SiMe_3_)_2_] (CNAd=1-adamantyl isocyanide) from [Pt_3_(CNAd)_6_] using 30 equivalents of hexamethyldisilane at 80 °C.[9]

It is well documented that N-heterocyclic carbenes (NHCs) have equivalent or better σ-donor character than the most common phosphines and that NHC/M complexes (M=metal) are less prone to decomposition by cleavage of the (NHC)–M bond.[10,11] As part of our ongoing studies of NHC/palladium-catalyzed reactions, we decided to explore their potential to activate hexamethyldisilane since there are no examples in the literature of palladium complexes capable of this reaction. Herein we report on the synthesis of the novel complex *cis*-[Pd(ITMe)_2_(SiMe_3_)_2_] from [(ITMe)_2_Pd^0^] and its inclusion in a catalytic cycle leading to the *cis*-bis(silyl)ation of alkynes.

While very recent literature on NHC/Pd complexes features the use of large NHCs as a common denominator,[12] we decided to start our studies with one of the smallest NHCs available, that is, 1,3,4,5-tetramethylimidazol-2-ylidene (ITMe; **1**), which exhibits a very small percent buried volume and a high σ-donor character.[13] The conventional synthetic route to **1** was established by Kuhn and co-workers.[14] It involves the formation of the corresponding thione by a ring-forming double condensation of *N*,*N*′-dimethylthiourea and acetoin, and subsequent reductive desulfurization of the thione using potassium metal, with an overall yield of about 76 %. A thorough modification of the synthetic protocol, including a microwave-mediated cyclization step, allowed us to obtain **1** in 86 % overall yield even on a gram scale (Scheme 1).[15]

**Scheme 1:**
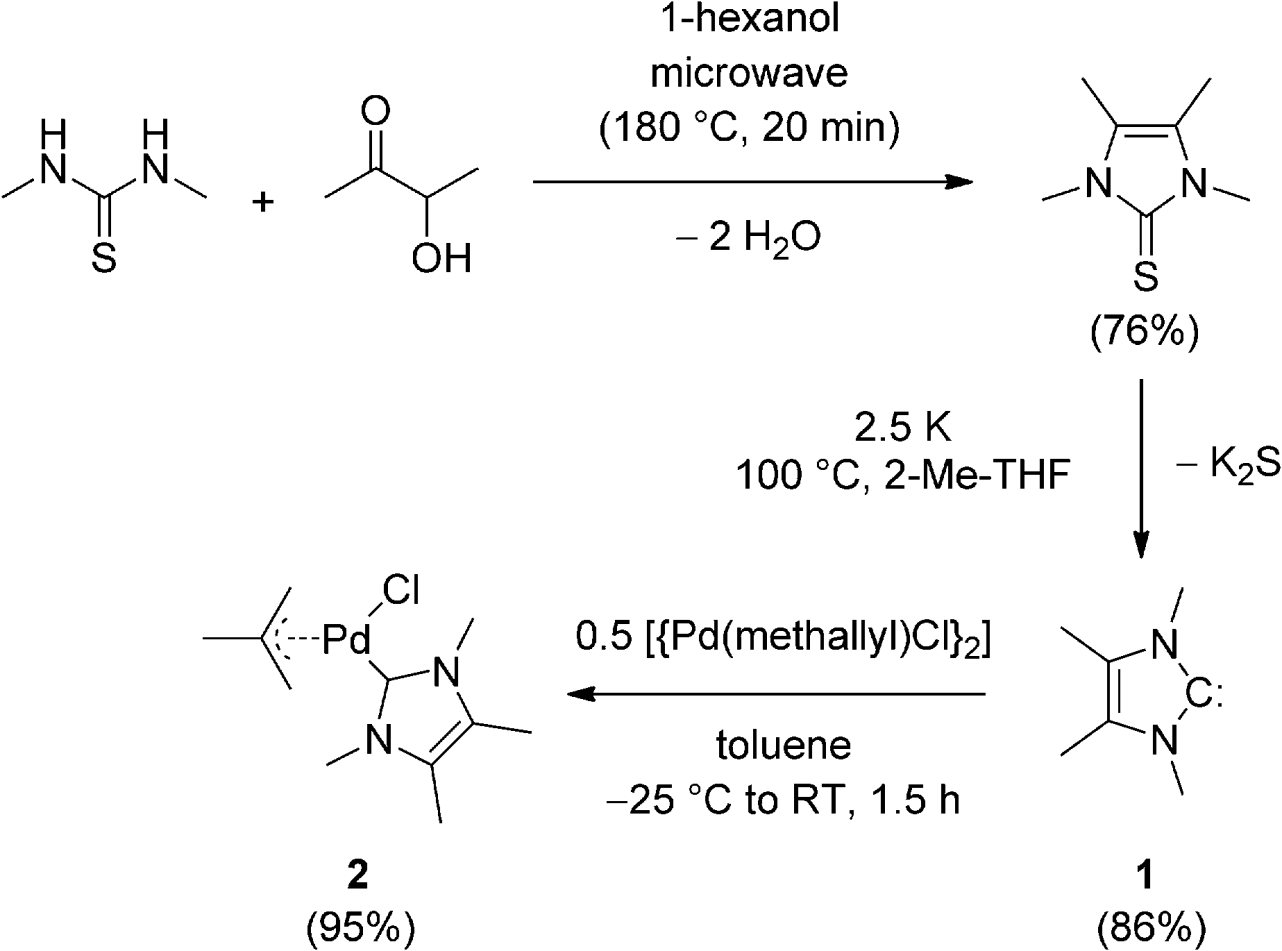
Synthesis of 1 and 2. 2-Me-THF=2-methyltetrahydrofuran.

The second step involved the synthesis of [(ITMe)Pd(methallyl)Cl] (**2**). The synthesis of this complex has not been reported, although unsuccessful attempts were detailed by Cavell and co-workers.[16] The conventional synthetic route to [(NHC)Pd(R-allyl)Cl] species involves the reaction of the corresponding [{Pd(R-allyl)Cl}_2_] dimer with a free NHC.[17] We found that reacting [{Pd(methallyl)Cl}_2_] with a slight excess of **1** in toluene, initially at −25 °C, and then warming to ambient temperature, led to the formation of **2** in 95 % yield (Scheme 1).

[(ITMe)_2_Pd^0^] (**3**) has been proposed as the active catalytic species in a number of reactions.[18] The only reported synthesis of **3** was achieved through metal vapor synthesis (MVS).[19] Recently, Fantasia and Nolan used [(NHC)Pd(allyl)Cl] complexes as precursors to easily synthesize a series of [(NHC)_2_Pd^0^] complexes,[20] suggesting that the solvent employed in these reactions (isopropanol) was also a reagent and essential to the mechanism of these transformations. Unfortunately, the application of this methodology to the synthesis of **3** resulted in the precipitation of large quantities of Pd black. Modifying this procedure by using isopropanol in stoichiometric quantities resulted in the first solution-based synthesis of **3**, which was formed as a yellow crystalline precipitate. Its isolation, however, proved difficult because of its limited solubility in toluene, THF, and pyridine, and its instability in alcohols or halogenated solvents. Consequently, the reaction mixture was directly reacted with hexamethyldisilane at room temperature for 18 hours (Scheme 2). *cis*-[(ITMe)_2_Pd(SiMe_3_)_2_] (**4**) was collected as an off-white solid in 62 % yield.

**Scheme 2:**
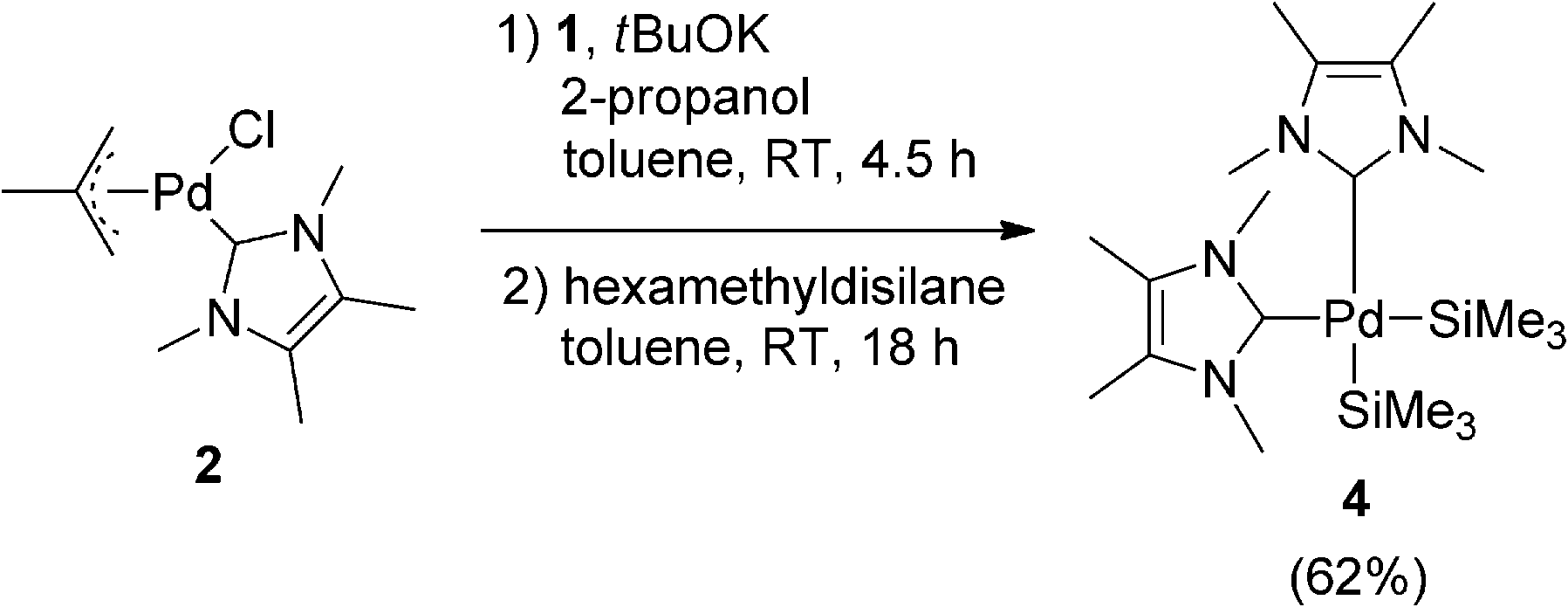
Synthesis of 4.

Single crystals of **4** were isolated from a saturated solution of toluene at −30 °C, and X-ray analysis revealed that **4** displays a marginally distorted square-planar geometry with the two NHCs in a *cis* configuration and orthogonal to the Si-Pd-Si plane (Figure 1). The Pd–Si bond lengths are comparable with those found in similar complexes such as [(dcpe)Pd(SiMe_2_H)_2_] (dcpe=1,2-bis(dicyclohexylphosphino)ethane).[6a

**Figure 1:**
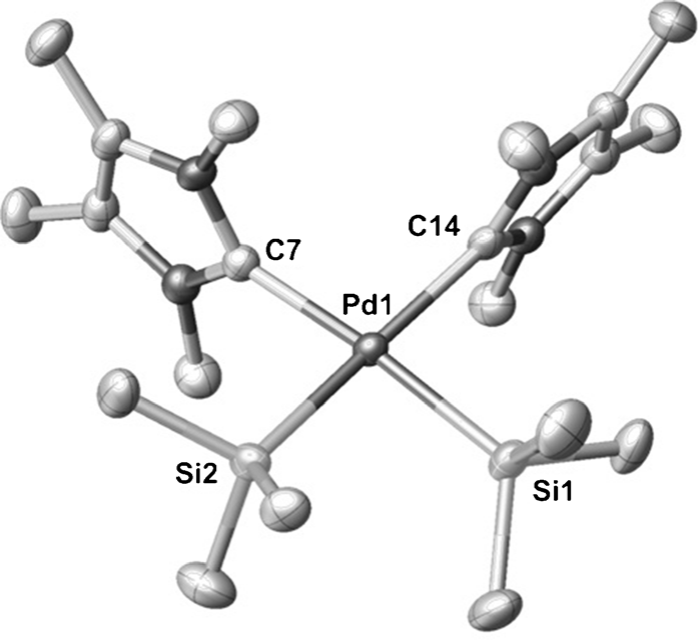
Molecular structure of 4 with thermal ellipsoids at the 50 % probability level.[29] Hydrogen atoms are omitted for clarity. Selected bond lengths [Å] and angles [°]: Pd–Si1: 2.3557(6), Pd–Si2 2.3468(6), Pd–C7 2.102(2), Pd–C14 2.119(2); Si1-Pd-Si2 88.65(2), Si1-Pd-C14 88.44(6), Si2-Pd-C7 88.74(6), C7-Pd-C14 94.74(8).

The reactivity of **4** was then investigated. A solution of **4** in C_6_D_6_ was heated to 85 °C and resulted in an intense yellow solution in less than 1.5 hours. ^1^H NMR analysis showed the formation of hexamethyldisilane and **3** (Scheme 3). The formation of the reductive elimination products was limited to 69 % conversion, even upon increasing the temperature and heating time, probably because of the recombination of the two products to form **4**.

**Scheme 3:**
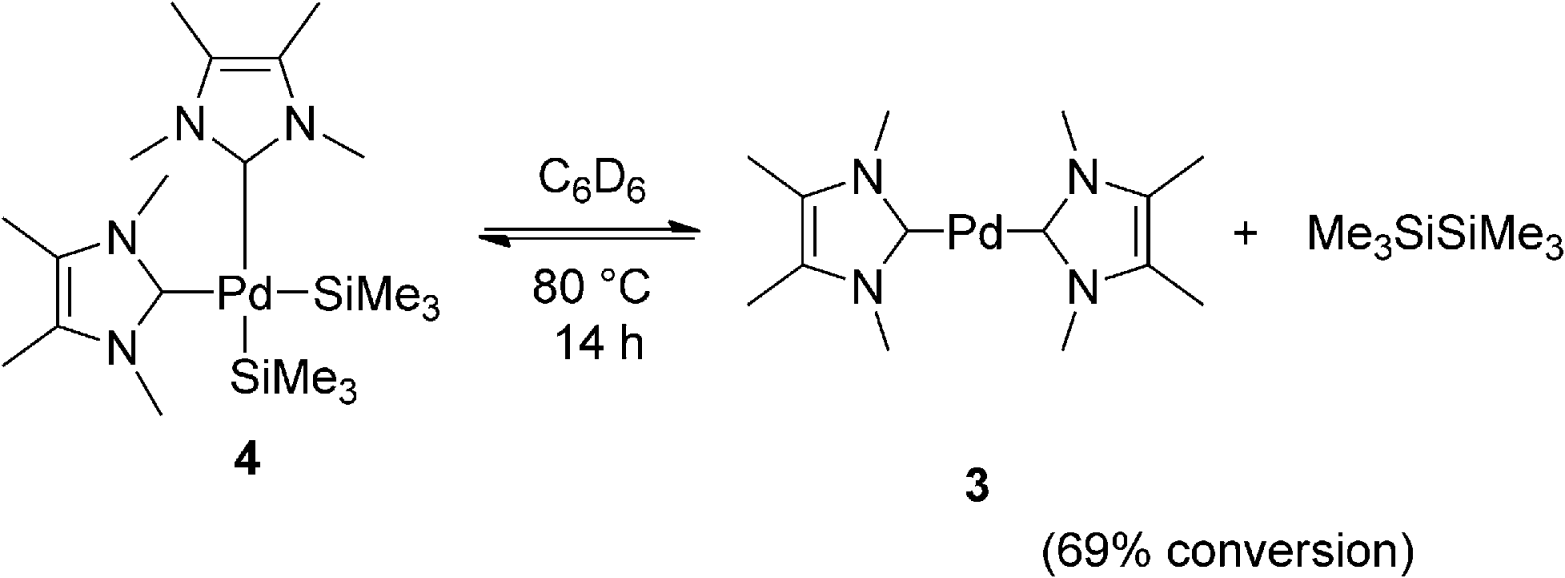
Reversible formation/decomposition of 4.

To our knowledge, the bis(silyl)ation of disubstituted alkynes using hexamethyldisilane has not been reported. The stoichiometric reaction of **4** with diphenylacetylene at room temperature yielded the corresponding *cis*-bis(silyl)ated product **5** (Scheme 4) within 30 hours in quantitative yield (see the Supporting Information).[21] This reaction also resulted in the quantitative formation of the novel complex [(ITMe)_2_Pd(PhC—CPh)] (**6**), which could be easily isolated after an *n*-hexane extraction. Compound **6** was fully characterized by NMR spectroscopy and elemental analysis. Single crystals of **6** were obtained from a saturated toluene solution upon cooling to −30 °C and the result of X-ray analysis is depicted in Figure 2, featuring a Y-shaped structure. There is a clear and expected elongation of the C—C bond and a shortening of the C—C—C bond when compared to free diphenylacetylene.[22] To the best of our knowledge, this is the first example of an NHC/Pd^0^ complex bearing an η^2^-bound alkyne. This species can react with an excess of hexamethyldisilane at 50 °C for over 5 days, resulting in the formation of **4** and **5** (Scheme 5).

**Figure 2:**
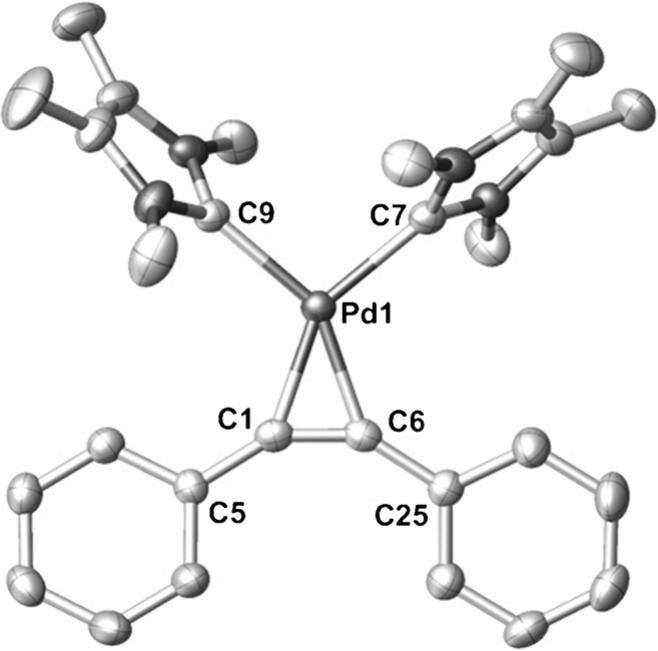
Molecular structure of 6 with thermal ellipsoids at the 50 % probability level.[29] Hydrogen atoms are omitted for clarity. Selected bond lengths [Å] and angles [°]: Pd1–C1 2.033(3), Pd1–C6 2.029(3), C1–C6 1.290(4); C6-C1-C5 147.5(3), C1-C6-C25 146.03(3).

**Scheme 4:**
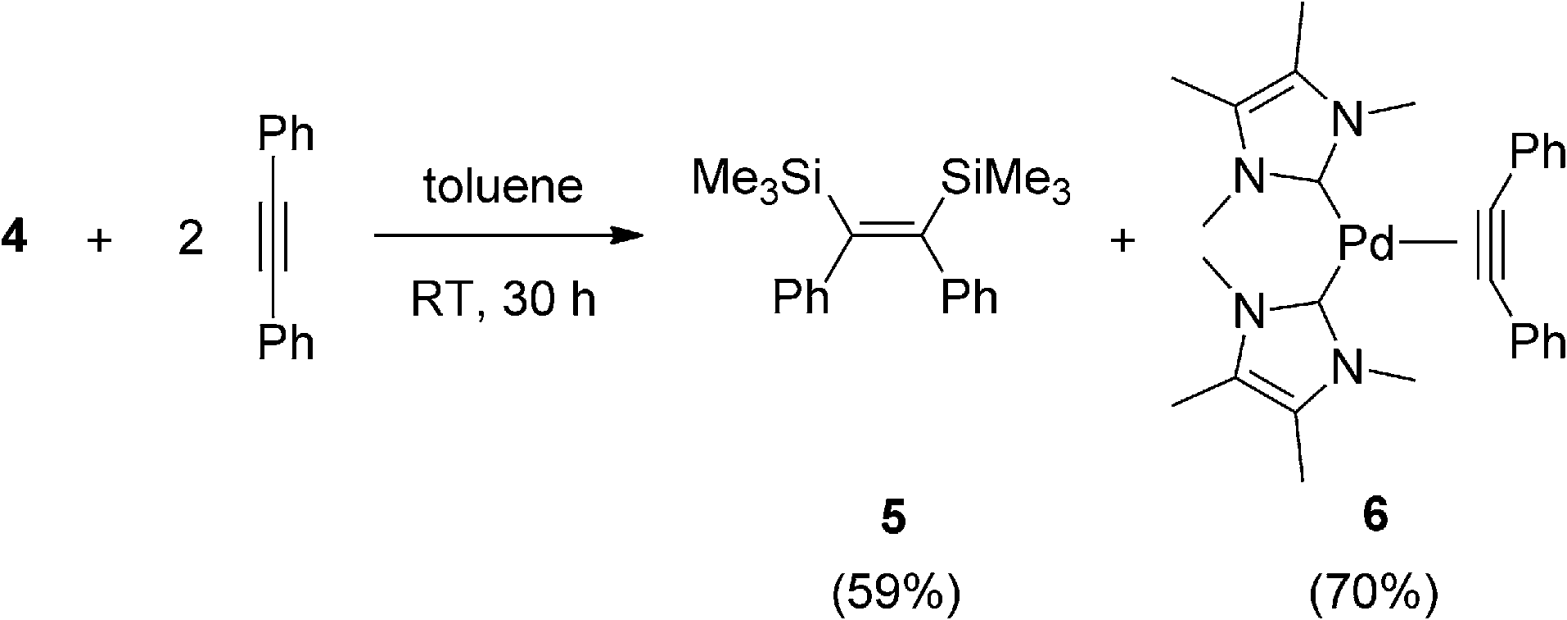
Stoichiometric bis(silyl)ation of diphenylacetylene.

**Scheme 5:**
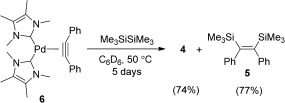
Reaction of 6 with hexamethyldisilane.

With all this information in hand, we proceeded to the inclusion of all these organometallic species into a catalytic cycle. Diphenylacetylene and hexamethyldisilane were selected as model substrates for the initial optimization of the reaction parameters. To our delight, 100 % stereoselective conversion into **5** (yield=94 %) was observed using 1 mol % of **4** (100 °C for 24 h in C_6_D_6_).[21] This reaction is the first reported catalytic synthesis of **5**. To test the versatility of **4** towards a range of challenging electronics and sterics surrounding the C—C bond, a series of internal alkynes and non-activated disilanes were also used as substrates. For instance, the reaction of diphenylacetylene and an excess of PhMe_2_SiSiMe_2_Ph yielded compound **7** (Scheme 6). The only synthesis reported for this compound involved the stoichiometric reaction of *cis*-[(PPh_2_Me)_2_Pt(SiMe_2_Ph)_2_] with diphenylacetylene.[23] The novel compounds **8**, **9**, **10**, and **11** were all synthesized as *Z* isomers from the corresponding unsymmetrical internal acetylenes and excess hexamethyldisilane. Compound **10** was synthesized with greater than 90 % conversion into the desired product. However, its isolation from the crude reaction mixture proved troublesome, and after numerous attempts a maximum of 41 % of the desired compound was obtained. The reaction of hexamethyldisilane with 1-phenyl-2-trimethylsilylacetylene resulted in the formation of **12** (49 % yield), but it required a considerable increase in catalyst loading and reaction time. The only previous reported syntheses of **12** required either the stoichiometric addition of Grignard reagents with acetylenes[24] or the addition of methyl lithium to a silyldisilacyclobutene.[25] The protocol was applied to a terminal alkyne, phenylacetylene, affording compound **13**, which was synthesized in a yield comparable to that of the best catalytic protocol in the literature.[26] Unfortunately, the reaction of hexamethyldisilane with 2-heptyne under these reaction conditions gave very low conversion into the desired product (<5 %).

**Scheme 6:**
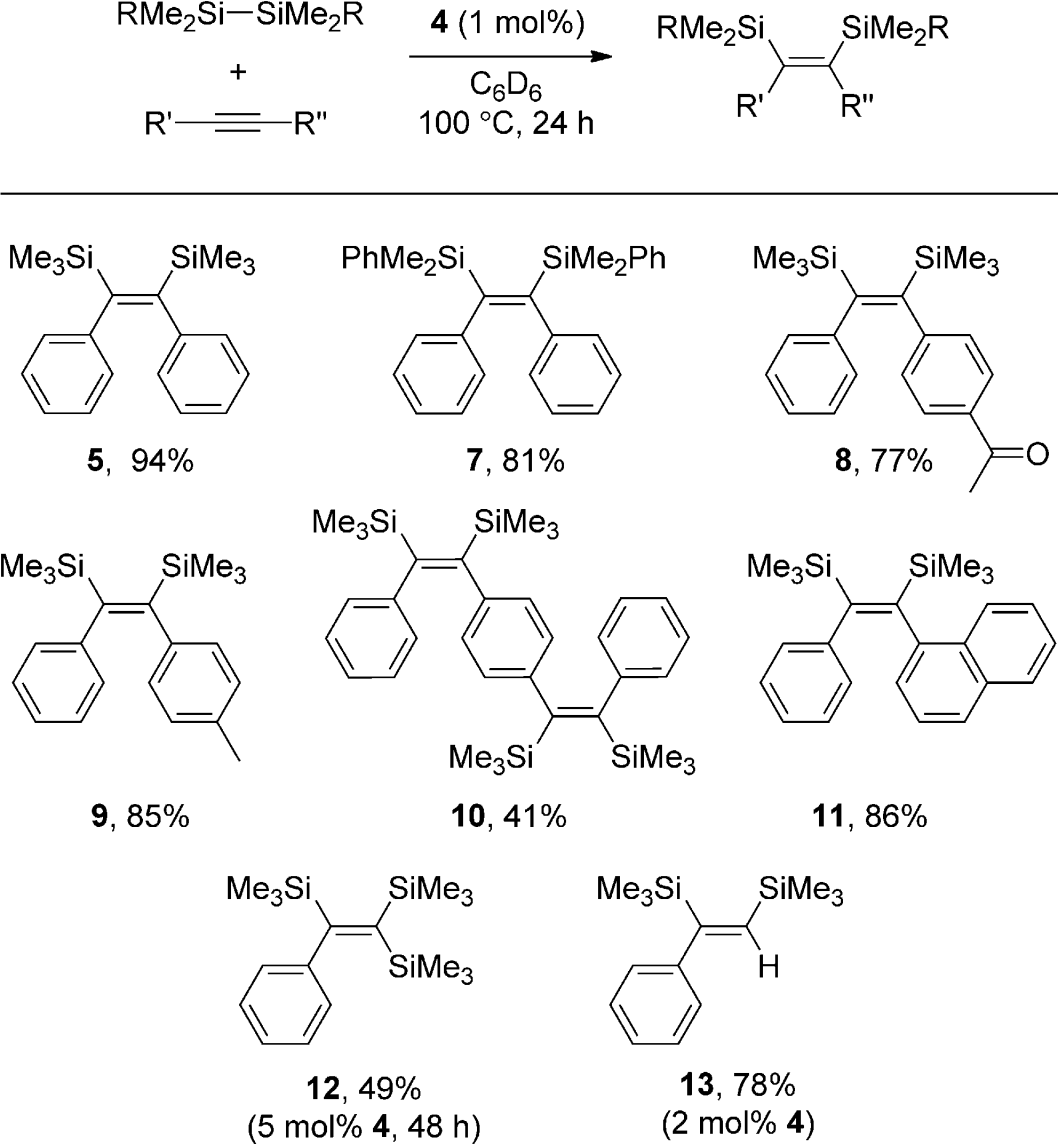
Catalytic bis(silyl)ation reactions.

The results from the catalytic reactions and the isolation of **4** and **5** prompted us to propose a mechanism for the catalytic cycle, in which **3** is the catalytically active species (Scheme 7). This 14 electron species can oxidatively add hexamethyldisilane, thus yielding **4**, followed by a migratory insertion of a silyl group into the acetylene to give the corresponding vinyl-palladium-silyl complex.[27] This complex would be stabilized by a weak interaction between the silicon from the vinylsilyl moiety and the palladium center,[28] thus allowing a stereoselective reductive elimination to yield **5**. The coordination of diphenylacetylene to **3** affords **6**, which could be considered the resting state of **3.**

**Scheme 7:**
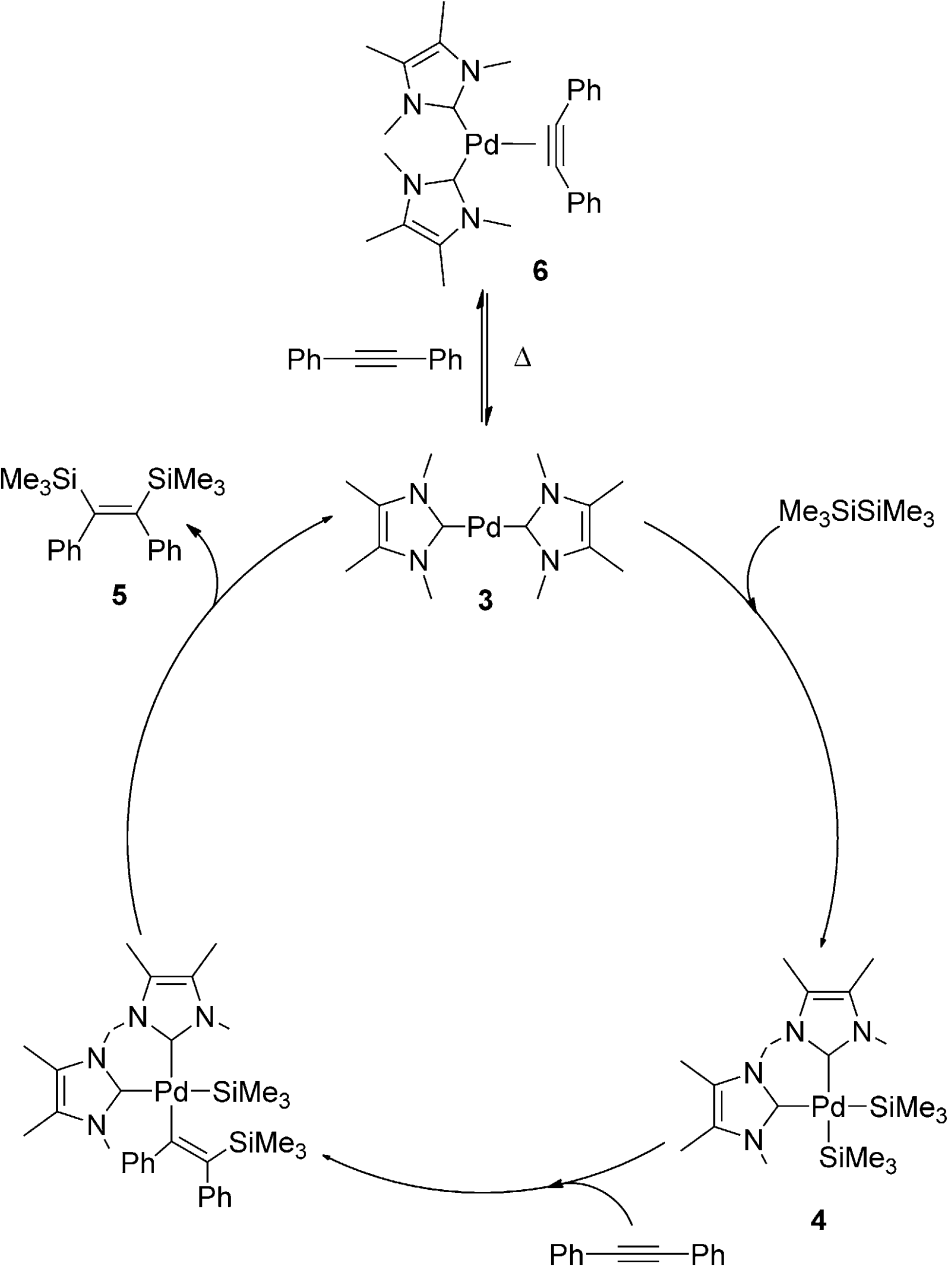
Proposed catalytic cycle for the bis(silyl)ation of internal acetylenes.

In conclusion, we have synthesized the first NHC-bearing complex resulting from the oxidative addition of hexamethyldisilane to a palladium center at room temperature. This complex was used as a precatalyst for the bis(silyl)ation of electronically and sterically challenging internal acetylenes using non-activated disilanes. A series of novel 1,2-disilyl-stilbenes were synthesized in high yield and with 100 % *Z* stereoselectivity. Studies on the activity of **4** in this and other catalytic processes are currently ongoing in our laboratories.

In memory of Mike Lappert
