# Supplementary material for: Synthesis of an [(NHC)2Pd(SiMe3)2] Complex and Catalytic cis-Bis(silyl)ations of Alkynes with Unactivated Disilanes
Source: Angew Chem Int Ed Engl. 2015 Apr 9;54(19):5578–82. doi: 10.1002/anie.201501764 (PMC4449112; doi:10.1002/anie.201501764)
Supplement: Supplementary file 1 [file anie0054-5578-sd1.pdf]

Angewandte Chemie
Eine Zeitschrift der Gesellschaft Deutscher Chemiker

Supporting Information

German Edition: DOI:

Synthesis of an $[(NHC)_2Pd(SiMe_3)_2]$ Complex and Catalytic *cis*-Bis(silyl)ations of Alkynes with Unactivated Disilanes**

Melvyn B. Ansell, Debbie E. Roberts, F. Geoffrey N. Cloke, Oscar Navarro, and John Spencer**

anie_201501764_sm_miscellaneous_information.pdf

Table of Contents

Synthesis and Catalysis..S1

Spectroscopic Data..S12

Crystallographic Data..S43

cis-[Pd(ITMe)$_2$(SiMe$_3$)$_2$] (4)... .S43

Pd(ITMe)$_2$(PhCCPh) (6).. S53

cis-(Me$_3$Si)(Ph)C=C(Ph)(SiMe$_3$) (5)..S63

1,4-bis((Z)-2-phenyl-1,2-bis(trimethylsilyl)vinyl)benzene (10)..........................S71

cis-(Ph)(Me$_3$Si)C=C(SiMe$_3$)(naphthalene) (11)..S78

References...S86

General methods, instrumentation and starting materials:

The manipulation of air sensitive compounds and their spectroscopic measurements were undertaken using standard Schlenk line techniques under pre-dried argon (using a BASF R3-11(G) catalyst and 4 Å molecular sieves), or in a MBraun glovebox under N_2 (O_2 < 10.0 ppm). All glassware was dried in a 160 ^{o}C oven prior to use. All solvents used for air sensitive compounds were dried by vacuum distillation followed by distillation over potassium (hexane and toluene) or stored over activated 4 Å molecular sieves under an Ar atmosphere (2-methyl tetrahydrofuran). Dried solvents were degassed and stored over argon in ampoules containing activated 4 Å molecular sieves. 1-Hexanol was used as purchased (Fischer). Deuterated benzene, C_6D_6, was degassed and dried by refluxing over potassium for 3 days, vacuum transferred into ampoules and stored under N_2. Deuterated chloroform, $CDCl_3$, was used as purchased.

NMR spectra were recorded on a Varian VNMRS 400 (^{1}H 399.5 MHz; ^{13}C{^{1}H} 100.46 MHz; ^{29}Si{^{1}H} 79.4 MHz) or 500 (^{1}H 499.91 MHz; ^{13}C{^{1}H} 125.71 MHz). The spectra were referenced to the corresponding protic solvent (^{1}H) or signals of the solvent (^{13}C). ^{29}Si{^{1}H} were referenced externally relative to the internal standard $SiMe_4$. All spectra carried out on the Varian VNMRS 400 and VNMRS 500 were recorded at 303 K.

Elemental analyses were carried out at the Elemental Analysis Service, London Metropolitan University. High resolution mass spectrometry was carried out by the EPSRC UK National Mass Spectrometry Facility, University of Swansea.

Improved synthesis of 1,3,4,5-tetramethylimidazol-2-thione

A microwave vial was charged with N,N'dimethylthiourea (4.34 g, 41.67 mmol), acetoin (3.74 g, 42.45 mmol), 1 spatula of $MgSO_4$ and 1-hexanol (60.0 ml). The resulting mixture was heated in the microwave at 185 °C for 20 mins (Dynamic mode – 300W). After removing all volatiles, the resulting off-white solid was washed with cold diethyl ether (3 x 20.0 ml). Yield: 5.70 g, 76 %. ^{1}H NMR (499.91 MHz, CDCl3): δ = 3.55 [s, 6H, N(1,3)-C**H3**], 2.09 [s, 6H, C(4,5)-C**H**$_3$]. ^{13}C{^{1}H} NMR (125.71 MHz, $CDCl_3$): δ = 161.8 [s, **C**(2)], 121.1 [s, **C**(4,5)], 32.3 [s, N(1,3)-**C**H_3], 9.6 [s, C(4,5)-**C**H_3].

^{1}H NMR (399.5 MHz, C_6D_6): δ = 3.13 [s, 6H, N(1,3)-C**H**$_3$], 1.25 [s, 6H, C(4,5)-C**H**$_3$].

Adjusted synthesis of 1,3,4,5-tetramethylimidazol-2-ylidene (ITMe) (1)

In an ampoule, 1,3,4,5-tetramethylimidazol-2-thione (1.50 g, 9.58 mmol) and potassium (0.95 g, 24.30 mmol) were suspended in 2-methyl THF (45.0 ml). The resulting reaction mixture was heated to 100 °C for 20 h. After cooling the mixture was filtered by an air-sensitive frit, the volatiles were removed in vacuo and the resulting off-white solid was dried under vacuum. Yield: 1.02 g, 86 %. ^{1}H NMR (399.5 MHz, C_6D_6): δ = 3.37 (s, 6H, N(1,3)-C**H**$_3$), 1.60 (s, 6H, C(4,5)-C**H**$_3$).

Synthesis [Pd(methallyl)(ITMe)Cl] (2)

[Pd(methallyl)Cl]$_2$ (0.44 g, 1.12 mmol) in toluene (20.0 ml) was cooled to −25 °C. A toluene (15.0 ml) solution of **1** (0.28 g, 2.25 mmol) was added dropwise over a 20 min period. After stirring at −25 °C under an Ar atmosphere for 1 h, the reaction mixture was then allowed to warm to ambient temperature and stirred for a further 1.5 h. The solution was then filtered by cannula, volatiles were removed in vacuo and the pale brown-grey solid was washed with hexane (3 x 10.0 ml). Yield = 0.68 g, 95 %.^{1}H NMR (399.5 MHz, C_6D_6): δ = 4.18 (s, 1H,

H$_2$CCMeCH$_2$), 3.27 (s, 6H, N(1,3)-C**H**$_3$), 3.25 (s, 1H, **H**$_2$CCMeCH$_2$), 2.86 (s, 1H, H$_2$CCMeC**H**$_2$), 2.16 (s, 1H, H$_2$CCMeC**H**$_2$), 1.73 (s, 3H, H$_2$CC**Me**CH$_2$), 1.33 (s, 6H, C(4,5)-C**H**$_3$). ^{13}C{^{1}H} (100.46 MHz, C$_6$D$_6$): δ = 178.7 (s, N**C**N), 124.6 (s, **C**(4,5)-CH$_3$), 124.6 (s, H$_2$C**C**MeCH$_2$) 70.3 (s, H$_2$**C**CMeCH$_2$), 47.1 (s, H$_2$CCMe**C**H$_2$), 35.1 (s, N(1,3)-**C**H$_3$), 23.7 (s, H$_2$CC**Me**CH$_2$), 8.5 (s, C(4,5)-**C**H$_3$). Elem. Anal.: Calcd for C$_{11}$H$_{19}$N$_2$ClPd: C, 41.10 %; H, 5.96 %; N, 8.72 %.Found: C, 40.98 %; H, 5.92 %; N, 8.75%.

Synthesis of *cis*-[Pd(ITMe)$_2$(SiMe$_3$)$_2$] (4)

In the glove box, **2** (0.50 g, 1.55 mmol), *t*-BuOK (0.17 g, 1.55 mmol) and **1** (0.20 g, 1.57 mmol) were suspended in toluene (20.0 ml). An isopropanol (0.09 g, 1.55 mmol) toluene (5.0 ml) solution was added and the reaction mixture was stirred at ambient temperature for 4.5 h. Me$_3$SiSiMe$_3$ (1.14 g, 7.81 mmol) was then added and the solution stirred for a further 18 h. The mixture was then filtered by cannula, volatiles were removed in vacuo and the resulting off-white solid was washed with hexane (3 x 5.0 ml). Yield: 0.48 g, 62 %. ^{1}H NMR (399.5 MHz, C$_6$D$_6$): δ = 3.35 (s, 12H, N(1,3)-C**H**$_3$), 1.41 (s, 12H, C(4,5)-C**H**$_3$), 0.59 (s, 18H, Si(C**H**$_3$)$_3$). ^{13}C{^{1}H} NMR (100.46 MHz, C$_6$D$_6$): δ = 196.7 (s, N**C**N), 123.6 (s, **C**(4,5)-CH$_3$), 35.2 (s, N(1,3)-**C**H$_3$), 9.5 (s, Si(**C**H$_3$)$_3$), 8.8 (s, C(4,5)-**C**H$_3$). ^{29}Si{^{1}H} NMR (79.4 MHz-, C$_6$D$_6$): δ = 0.46. Elem. Anal.: Calcd for C$_{20}$H$_{42}$N$_4$Si$_2$Pd: C, 47.93 %; H, 8.45 %; N, 11.18 %. Found: C, 47.96 %; H, 8.51 %; N, 11.04%.

Heating of 4

In a Youngs tap NMR tube a solution of **4** (0.01 g, 19.36 μmol) in C$_6$D$_6$ (0.7 ml) was heated in a heating block to 85 ºC over 24 h. This resulted in partial reversible reductive elimination to **3** and Me$_3$SiSiMe$_3$. The maximum conversion to Me$_3$SiSiMe$_3$ and Pd(ITMe)$_2$ was observed as 69 % (suggested by integration of the SiMe$_3$ in ^{1}H NMR spectrum). Heating to

higher temperatures (95 °C) did not supersede this conversion. A small quantity of $Me_3SiOSiMe_3$ was observed over time (9 % of all $SiMe_3$ present).

$Pd(ITMe)_2$ (**3**): ^1H NMR (399.5 MHz, C_6D_6): δ = 3.93 (s, 12H, N(1,3)-CH_3), 1.55 (s, 12H, C(4,5)-CH_3).[1]

4: ^1H NMR (399.5 MHz, C_6D_6): δ = 3.35 (s, 12H, N(1,3)-CH_3), 1.42 (s, 12H, C(4,5)-CH_3), 0.58 (s, 18H, $SiMe_3$).

$Me_3SiSiMe_3$: ^1H NMR (399.5 MHz, C_6D_6): δ = 0.08 (s, 18H, $SiMe_3$).

$Me_3SiOSiMe_3$: ^1H NMR (399.5 MHz, C_6D_6): δ = 0.12 (s, 18H, $SiMe_3$).[2]

Synthesis of (*Z*)-1,2-diphenyl-1,2-bis(trimethylsilyl)ethene (5) and Pd(ITMe)$_2$(PhCCPh) (6)

Isolation of 6

In an ampoule a solution of **4** (0.05 g, 96.4 μmol) and diphenylacetylene (0.03 g, 193.0 μmol) in C_6D_6 (5.0 ml) was stirred at ambient temperature for 30 h. The volatiles were then removed in vacuo. The resulting yellow solid was washed with hexane (3 x 10.0 ml). Yield: 0.04 g, 70 %. ^1H NMR (399.5 MHz, C_6D_6): δ = 7.99 (dd, $^3J_{HH}$ = 8.1, $^4J_{HH}$ = 1.3 Hz, 4H, *m*-C_6H_5), 7.27 (m, 4H, *o*-C_6H_5), 7.05 (tt, $^3J_{HH}$ = 7.3, $^4J_{HH}$ = 1.2 Hz, 2H, *p*-C_6H_5), 3.49 (s, 12H, N(1,3)-C**H**$_3$), 1.54 (s, 12H, C(4,5)-CH_3). $^{13}C\{^1H\}$ NMR (100.46 MHz, C_6D_6): δ = 198.7 (s, N**C**N), 138.6 (s, C≡**C**), 130.1 (s, *o*-C_6H_5), 128.2 (s, *m*-C_6H_5), 126.3 (s, *i*-C_6H_5), 124.2 (s, *p*-C_6H_5), 123.1 (s, **C**(4,5)-CH_3), 35.2 (s, N(1,3)-**C**H$_3$), 9.0 (s, C(4,5)-**C**H$_3$). Elem. Anal.: Calcd for $C_{28}H_{34}N_4Pd$: C, 63.03 %; H, 6.43 %; N, 10.51 %. Found: C, 62.87 %; H, 6.56 %; N, 10.46 %.

Isolation of 5

The hexane washes from the previous reaction were concentrated down and washed with water (3 x 10.0 ml). The organic layer was collected and dried with anhydrous $MgSO_4$. After filtering and washing the $MgSO_4$ with hexane (3 x 5.0 ml). The filtrates volatiles were removed in vacuo to reveal a white crystalline solid. Melting point: 87.4 – 88.7 °C. Yield: 0.02 g, 59 %. ^{1}H NMR (399.5 MHz, C_6D_6): δ = 6.93 (m, 4H, *o*- C_6H_4), 6.76 (m, 2H, *p*-C_6H_4), 6.69 (m, 4H, *m*-Ph-H), 0.21 (s, 18H, $SiMe_3$).^{1}H NMR (499.9 MHz, $CDCl_3$): δ = 6.97 (m, 4H, C_6H_5), 6.85 (m, 2H, *p*-C_6H_5), 6.63 (m, 4H, C_6H_5), 0.13 (s, 18H, $SiMe_3$). ^{1}H NMR (399.5 MHz, CCl_4): δ = 6.73 (m, 10 H, C_6H_5), 0.14 (s, 18H, $SiMe_3$). ^{13}C{^{1}H} (125.72 MHz, C_6D_6): δ = 158.9 (s, C=**C**), 146.8 (s, *i*-C_6H_5), 128.4 9s, *m*-C_6H_5), 127.6 (s, *o*-C_6H_5), 124.8 (s, *p*-C_6H_5), 1.8 (s, Si**Me**$_3$). ^{29}Si{^{1}H} NMR (79.4 MHz, C_6D_6): δ = −7.75. Elem. Anal.: Calcd for $C_{20}H_{28}Si_2$: C, 74.00 %; H, 8.69%. Found: C, 73.86%; H, 8.63 %. HRMS (APCI) m/z: [M + H]$^+$ Calcd for $C_{20}H_{28}Si_2H$ 325.1802; Found 325.1809.

Synthesis of 4 and 5 from 6

Isolation of 4

In an ampoule, **6** (0.02 g, 41.28 μmol) and $Me_3SiSiMe_3$ (0.03 g, 206.31 μmol) in toluene (5.0 ml) under a N_2 atmosphere was heated at 50 ºC for 5 d. On cooling the volatiles were removed in vacuo and the off-white solid was washed with hexane (3 x 3.0 ml). Yield: 0.015 g, 74 %. ^{1}H NMR (399.5 MHz, C_6D_6): δ = 3.34 (s, 12H, N(1,3)-CH_3), 1.41 (s, 12H, C(4,5)-CH_3), 0.59 (s, 18H, $SiMe_3$).

Isolation of 5

The hexane washes from the previous reaction were concentrated and washed with water (3 x 15.0 ml). The organic layer was collected collected had its volatiles removed in vacuo to

reveal **5,** a white crystalline solid. Yield: 0.01 g, 77 %. ^{1}H NMR (399.5 MHz, C_6D_6): δ = 6.93 (m, 4H, *o*-C_6H_5), 6.77 (m, 2H, *p*-C_6H_5), 6.70 (m, 4H, *m*-C_6H_5), 0.21 (s, 18H, $SiMe_3$).

Catalysis using 4 (1 mol %)

Synthesis of 5

In separate ampoules two reaction mixtures containing $Me_3SiSiMe_3$ (153.0 μl, 0.75 mmol), diphenylacetylene (0.09 g, 0.50 mmol) and **4** (0.003 g, 4.99 μmol) in C_6D_6 (0.75 ml) were heated at 100 ºC for 24 h under a N_2 atmosphere. After cooling the samples were combined. The volatiles were removed in vacuo to reveal an off-white solid. This was re-dissolved in hexane (30.0 ml), filtered through a plug of silica and washed with water (3 x 20.0 ml). The hexane solution was collected and its volatiles were removed in vacuo. The resulting off-white solid was washed with water (1 x 20.0 ml) and dried under vacuum. A white powdered solid resulted. Yield: 0.37 g, 94 %. ^{1}H NMR (399.5 MHz, C_6D_6): δ = 6.92 (m, 4H, C_6H_5), 6.76 (m, 2H, *p*-C_6H_5), 6.70 (m, 4H, C_6H_5), 0.21 (s, 18H, $SiMe_3$).

Synthesis of (*Z*)-1,2-bis(dimethyl(phenyl)silyl)-1,2-diphenylethene (7)

In an ampoule, a mixture of $PhMe_2SiSiMe_2Ph$ (0.10 g, 0.37 mmol), diphenylacetylene (0.05 g, 0.25 mmol) and **4** (0.001 g, 2.49 μmol) in C_6D_6 (0.25 ml) was heated to 100 ºC for 24 h under a N_2 atmosphere. After cooling the volatiles were removed in vacuo. The resulting solid was re-dissolved in hexane (30.0 ml) and subsequently washed with H_2O (3 x 20.0 ml). The organic fraction was collected and concentrated. The crude material was purified on silica gel (hexane) to afford pure white powdered solid. Yield: 0.18 g, 81 %. ^{1}H NMR (399.5 MHz, $CDCl_3$): δ = 7.47 (m, 4H), 7.32 (m, 6H), 6.92 (m, 4H), 6.82 (m, 2H), 6.67 (m, 2H), 0.09 (s, 12H, Si**Me**$_2$Ph).^{1}H NMR (399.5 MHz, C_6D_6): δ = 7.57 (m, 4H), 7.23 (m, 6H), 6.87 (m, 4H), 6.78-6.70 (m, 6H), 0.21 (s, 12H, Si**Me**$_2$Ph).^{13}C{^{1}H} NMR (125.72 MHz, C_6D_6): δ =

158.9 (s, **C**=C), 146.4 (s, *i*-Ph), 139.9 (s, $SiMe_2$**Ph**), 134.9 (s, $SiMe_2$**Ph**), 129.7 (s, $SiMe_2$**Ph**), 128.7 (s, $SiMe_2$**Ph**), 128.4 (s, Ph), 127.5 (s, Ph), 125.0 (s, *p*-Ph), 1.0 (s, Si**Me_2**Ph).

Synthesis of (*Z*)-1-(4-(2-phenyl-1,2-bis(trimethylsilyl)vinyl)phenyl)ethanone (8)

In an ampoule, a mixture of 1-(4-(phenylethynyl)phenyl)ethanone (0.04 g, 0.19 mmol), $Me_3SiSiMe_3$ (59.0 μL, 0.29 mmol) and **4** (0.001 g, 1.94 μmol) in C_6D_6 (0.20 ml) was heated to 100 °C for 24 h under a N_2 atmosphere. After cooling the volatiles were removed in vacuo. The purple solid was re-dissolved in chloroform (20.0 ml) and washed with H_2O (3 x 20.0 ml). The chloroform fraction was collected and filtered through a plug of silica. The resulting filtrates solvent was removed in vacuo to reveal a pale yellow crystalline solid. This was washed with H_2O (15.0 ml) and dried under vacuum. Melting point: 65.1 - 68.3 °C. Yield: 0.05 g, 74 %. ^1H NMR (399.5 MHz, C_6D_6): δ = 7.60 (m, 2H, *m*-$C_6H_4C(O)CH_3$), 6.91 (m, 2H, *m*-C_6H_5), 6.74 (m, 1H, *p*-C_6H_5), 6.67 (m, 2H, *o*-$C_6H_4C(O)CH_3$), 6.64 (m, 2H, *o*-C_6H_5), 1.92 (s, 3H, -$C_6H_4C(O)C$**H_3**), 0.19 (s, 9H, $SiMe_3$), 0.17 (s, 9H, $SiMe_3$). ^1H NMR (399.5 MHz, $CDCl_3$): δ = 7.60 (d, $^3J_{HH}$ = 8.0 Hz, 2H, *m*-$C_6H_4C(O)CH_3$), 6.97 (m, 2H, *m*- C_6H_5), 6.85 (m, 1H, *p*- C_6H_5), 6.74 (d, $^3J_{HH}$ = 8.0 Hz, 2H, *o*-$C_6H_4C(O)CH_3$), 6.62 (m, 2H, *o*- C_6H_5), 2.46 (s, 3H, -$C_6H_4C(O)C$**H_3**), 0.14 (s, 9H, $SiMe_3$), 0.14 (s, 9 H, $SiMe_3$). $^{13}C\{^1H\}$ NMR (100.46 MHz, C_6D_6): δ = 195.8 (s, -C_6H_4**C**$(O)CH_3$), 159.5 (s, Ph**C**=C-), 158.1 (s, PhC=**C-**), 152.0 (s, *i*-$C_6H_4C(O)CH_3$), 146.4 (s, *i*- C_6H_5), 134.3 (s, *p*-$C_6H_4C(O)CH_3$), 128.4 (s, *o*-$C_6H_4C(O)CH_3$), 128.1 (s, *m*-$C_6H_4C(O)CH_3$), 127.8 (s, *o*- C_6H_5), 127.7 (s, *m*- C_6H_5), 25.8 (s, -$C_6H_4C(O)$**C**H_3), 1.73 (s, $SiMe_3$), 1.71 (s, $SiMe_3$). $^{13}C\{^1H\}$ NMR (125.72, $CDCl_3$): δ = 198.0 (s, -C_6H_4**C**$(O)CH_3$), 159.4 (s, (Ph)(Me_3Si)**C**=C-), 157.7 (s, -C=**C**($SiMe_3$)($C_6H_4C(O)CH_3$)), 152. 6 (s, *i*-$C_6H_4C(O)CH_3$), 146.2 (s, *i*- C_6H_5), 133.6 (s, *p*-$C_6H_4C(O)CH_3$), 128.1 (s, *o*-$C_6H_4C(O)CH_3$), 127.6 (s, *o*- C_6H_5), 127.6 (s, *m*-$C_6H_4C(O)CH_3$), 127.4 (s, *m*- C_6H_5), 124.7 (s, *p*- C_6H_5), 26.5 (s, -$C_6H_4C(O)$**C**H_3), 1.7 (s, $SiMe_3$), 1.6 (s, $SiMe_3$). $^{29}Si\{^1H\}$ NMR (79.4 MHz,

C_6D_6): δ = −7.38 (s, $SiMe_3$), −7.77 (s, $SiMe_3$). Elem. Anal.: Calcd for $C_{22}H_{30}OSi_2$: C, 72.07 %; H, 8.25 %. Found: C, 72.02 %; H, 8.37 %.HRMS (APCI) m/z: $[M + H]^+$ Calcd for $C_{22}H_{30}Si_2OH$ 367.1908; Found 367.1907.

Synthesis of (*Z*)-(1-phenyl-2-(p-tolyl)ethene-1,2-diyl)bis(trimethylsilane) (9)

In an ampoule, a mixture of $Me_3SiSiMe_3$ (54.0 µl, 0.26 mmol), 1-methyl-4-(phenylethynyl)benzene (0.03 g, 0.18 mmol) and **4** (0.001 g, 1.80 µmol) in C_6D_6 (0.25 ml) was heated to 100 ºC for 24 h under a N_2 atmosphere. After cooling the volatiles were removed in vacuo. The resulting solid was re-dissolved in $CHCl_3$ (20.0 ml), filtered through a plug of silica and washed with H_2O (30.0 ml). The organic fraction was collected, the volatiles removed and the resulting solid off-white powdered solid was washed with H_2O (20.0 ml). Melting point: 62.2 - 64.6 °C. Yield: 0.05 g, 90 %.

^1H NMR (399.5 MHz, C_6D_6): δ = 6.94 (m, 2H, *m*-C_6H_5), 6.74 (m, 5H, C_6H_5), 6.64 (m, 2H, *o*-$C_6H_4CH_3$), 1.92 (s, 3H, $C_6H_4C\mathbf{H_3}$), 0.24 (s, 9H, $SiMe_3$), 0.22 (s, 9H, $SiMe_3$). ^1H NMR (399.5 MHz, $CDCl_3$): δ = 6.98 (m, 2H, *m*- C_6H_5), 6.86 (m, 2H, *p*- C_6H_5), 6.77 (d, $^3J_{HH}$ = 7.6 Hz, 2H, *m*-$C_6H_4CH_3$), 6.63 (m, 2H, *o*-C_6H_5), 6.51 (d, $^3J_{HH}$ = 7.6 Hz, 2H, *o*-$C_6H_4CH_3$), 2.13 (s, 3H, $C_6H_4C\mathbf{H_3}$), 0.12 (s, 9H, $SiMe_3$), 0.12 (s, 9H, $SiMe_3$). $^{13}C\{^1H\}$ NMR (100.46 MHz, C6D6):δ = 158.9 (s, **C**=**C**), 147.0 (s, *i*- C_6H_5), 143.8 (s, *i*- $C_6H_4CH_3$), 133.9 (s, *p*- $C_6H_4CH_3$), 128.4 (s, *m*-$C_6H_4CH_3$), 128.1 (s, *o*- C_6H_5), 128.0 (s, *o*- $C_6H_4CH_3$), 124.8 (s, *p*- C_6H_5), 21.0 (s, $C_6H_4\mathbf{C}H_3$), 1.9 (s, $SiMe_3$), 1.9 (s, $SiMe_3$). $^{13}C\{^1H\}$ NMR (125.72 MHz, $CDCl_3$):δ =158.5 (s, -C=**C**($SiMe_3$)($C_6H_4CH_3$), 158.4 (s, (C_6H_5)($SiMe_3$)**C**=C-), 146. 8 (s, *i*-C_6H_5), 143.5 (s, *i*-$C_6H_4CH_3$), 133.5 (s, *p*-$C_6H_4CH_3$), 127.9 (s, *o*-C_6H_5), 127.9 (s, *m*-$C_6H_4CH_3$), 127.7 (s, *o*-$C_6H_4CH_3$),127.1 (s, *m*-C_6H_5), 124.2 (s, *p*-C_6H_5), 21.4 (s, $C_6H_4\mathbf{C}H_3$), 1.8 (s, $SiMe_3$), 1.8 (s, $SiMe_3$). $^{29}Si\{^1H\}$ (79.4 MHz, C_6D_6): δ = −7.84, −7.84. Elem. Anal.: Calcd for $C_{20}H_{30}Si_2$: C,

74.48 %; H, 8.93 %. Found: C, 74.35 %; H, 9.03 %. HRMS (APCI) m/z: $[M + H]^+$ Calcd for $C_{21}H_{30}Si_2H$ 339.1959; Found 339.1965.

Synthesis of 1,4-bis((Z)-2-phenyl-1,2-bis(trimethylsilyl)vinyl)benzene (10)

1,4-bis(phenylethynyl)bezene (0.047 g, 0.17 mmol), HMDS (95.0 µl, 0.46 mmol) and **4** (0.002 g, 3.39 µmol) was dissolved in C_6D_6 (0.6 ml). The resulting reaction mixture was heated to 100 ºC for 24 h under a N_2 atmosphere. On cooling the volatiles were removed in vacuo. The oily solid was washed with H_2O (20.0 ml) and extracted using $CHCl_3$ (30.0 ml). The organics were collected and filtered through a plug of silica. 1,4-bis((Z)-2-phenyl-1,2-bis(trimethylsilyl)vinyl)benzene was obtained by preparative TLC (100 % hexane). Melting point: 203.4 - 204.5 ºC. Yield: 0.039 g, 41.0 %. ^{1}H NMR (499.1 MHz, $CDCl_3$): δ = 6.89 (t, $^3J_{HH}$ = 7.7 Hz, 4H, *o*-Ph), 6.77 (t, $^3J_{HH}$ = 7.2 Hz, 2H, *p*-Ph), 6.53 (dd, $^3J_{HH}$ = 7.4, 0.9 Hz, 4H, *m*-Ph), 6.21 (s, 4H, brid-Ph), 0.08 (s, 18H, $SiMe_3$), −0.06 (s, 18H, $SiMe_3$). ^{13}C{^{1}H} NMR (100.46 MHz, $CDCl_3$): δ = 159.1 (s, C=**C**), 158.0 (s, **C**=C), 146.7 (s, *i*-Ph), 142.5 (s, *i*-brid-Ph), 127.9 (s, *o*-Ph), 127.1 (s, *m*-Ph), 126.6 (s, brid-Ph), 124.1 (s, *p*-Ph), 1.7 (s, $SiMe_3$), 1.7 (s, $SiMe_3$). ^{29}Si{^{1}H} NMR (79.4 MHz, $CDCl_3$): δ = −7.95. Elem. Anal.: Calcd for $C_{34}H_{50}Si_4$: C, 71.50 %; H, 8.82 %. Found: C, 71.34 %; H, 8.70 %. HRMS (ESI) m/z: [M + Na]+ Calcd for $C_{34}H_{50}Si_4$ 593.2882; Found 593.2890.

Synthesis of (*Z*)-(1-(naphthalen-1-yl)-2-phenylethene-1,2-diyl)bis(trimethylsilane) (11)

In an ampoule, a mixture of 1-(phenylethynyl)naphthalene (0.08 g, 0.35 mmol), $Me_3SiSiMe_3$ (106.0 µml, 0.52 mmol) and **4** (0.002 g, 3.39 µmol) in C_6D_6 (0.35 ml) was heated to 100 ºC for 24 h under a N_2 atmosphere. After cooling all volatiles were removed in vacuo revealing a brown oil. This was re-dissolved in $CDCl_3$ (20.0 ml), filtered through a plug of silica and washed with H_2O (3 x 20.0 ml). The organic layer was collected, the volatiles removed and

the resulting off-white powdered solid was washed with H_2O (20.0 ml). Melting point: 75.2-78.3 $^{\circ}$C. Yield: 0.11 g, 86 %. ^{1}H NMR (499.91 MHz, $CDCl_3$): δ = 7.82 (d, $^3J_{HH}$ = 8.0 Hz, 1H, 10-NA), 7.62 (d, $^3J_{HH}$ = 7.9 Hz, 1H, 7-NA), 7.39 (m, 2H, 4,9-NA), 7.34 (dd, $^3J_{HH}$ = 9.1, 7.0 Hz, 1H, 8-NA), 7.15 (dd, $^3J_{HH}$ = 8.5, 6.8 Hz, 1H, 3-NA), 6.90 (br, 1H, *m*-C_6H_5), 6.78 (dd, $^3J_{HH}$ = 6.9, 1.4 Hz, 1H, 2-NA), 6.69 (br, 1H, *o*-C_6H_5), 6.69 (dd, $^3J_{HH}$ = 8.7, 6.9 MHz, 1H, *p*-C_6H_5), 6.64 (br, 1H, *m*-C_6H_5), 6.52 (br, 1H, *o*-C_6H_5). ^{13}C{^{1}H} (125.72 MHz, $CDCl_3$): 160.1 (s, (Ph)(Me_3Si)**C**=C-), 156.8 (s, -C=**C**($SiMe_3$)(NA)), 146.4 (s, *i*- C_6H_5), 144.5 (s, *i*-NA), 133.3 (s, 5-NA), 131.3 (s, 6-NA), 128.1 (s, 7-NA), 128.1, 126.8 (s, 10-NA), 126.8, 125.2 (s, 4-NA), 125.2, 125.2, 125.2 (s, 8-NA), 124.8 (s, 2-NA), 124.8 (s, 9-NA), 124.8, 124.4 (s, 3-NA), 124.4. ^{29}Si{^{1}H} NMR (79.4 MHz, $CDCl_3$): δ = −7.00, −7.51. Elem. Anal.: Calcd for $C_{24}H_{30}Si_2$: C, 76.94 %; H, 8.07 %. Found: C, 76.87 %; H, 7.96 %. HRMS (APCI) m/z: [M + H]$^+$ Calcd for $C_{24}H_{30}Si_2H$ 375.1959; Found 375.1957.

Synthesis of 1-phenyl-1,2,2-tris(trimethylsilyl)ethylene (12)

In an ampoule, 1-phenyl-2-trimethylsilylacetylene (60.0 μl, 0.30 mmol), HMDS (94.0 μl, 0.46 mmol), *cis*-Pd(ITMe)$_2$($SiMe_3$)$_2$ (0.008 g, 15.16 μmol) were dissolved in C_6D_6 (0.5 ml). The resulting reaction mixture was heated to 100 °C for 48 h under a N_2 atmosphere. On cooling the volatiles were removed in vacuo. The white oily solid was re-dissolved in DCM and filtered through a plug of silica. The DCM was removed in vacuo and the white powdered solid washed with H_2O (20.0 ml). Yield: 0.048 g, 49.1 %. ^{1}H NMR (499.91 MHz, $CDCl_3$): δ = 7.19 (t, J_{HH} = 7.1 Hz, 2H, Ph), 7.13 (tt, J_{HH} = 7.2, 1.0 Hz, 1H, *p*-Ph), 6.82 (dd, J_{HH} = 7.4, 1.0 Hz, 2H, Ph), 0.32 (s, 9H, $SiMe_3$), 0.06 (s, 9H, $SiMe_3$), −0.27 (s, 9H, $SiMe_3$).

Synthesis of (Z)-1,2-bis(trimethylsilyl)-1-phenylethene (15)

In an ampoule, phenyacetylene (40.0 µl, 0.36 mmol), HMDS (112.0 µl, 0.54 mmol) and **4** (0.004 g, 7.18 µmol) were dissolved in C_6D_6 (0.35 ml). The resulting reaction mixture was heated to 100 ºC under a N_2 atmosphere for 24 h. On cooling the reaction mixture hexane (20.0 ml) was added. This solution was washed with H_2O (3 x 20.0 ml). The organic fractions were collected and filtered through a plug of silica. The low boiling point volatiles were then removed in vacuo. Crude product was purified on silica gel (hexane) to afford a colourless oil. Yield: 0.070 g, 77.8 %. ^{1}H NMR (399.5 MHz, $CDCl_3$): δ = 7.25 (m, 2H, Ph), 7.16 (t, $^3J_{HH}$ = 7.6 Hz, 1H, *p*-Ph), 7.03 (dd, J_{HH} = 7.6, 1.2 Hz, 2H, Ph), 6.43 (s, 1H, =CH), 0.21 (s, 9H, $SiMe_3$), 0.15 (s, 9H, $SiMe_3$). ^{13}C{^{1}H} NMR (100.46 MHz, $CDCl_3$): δ = 164.4, 151.1, 149.0, 127.9, 126.4, 125.7, 1.3, 1.1. ^{29}Si{^{1}H} NMr (79.4 MHz, $CDCl_3$): δ = −7.19, −10.67.

Catalysis with Pd(ITMe)$_2$(PhCCPh) (1 mol %)

Synthesis of 5

In an ampoule, a mixture of diphenylacetylene (0.03 g, 0.17 mmol), $Me_3SiSiMe_3$ (55.0 µl, 0.27 mmol) and **6** (0.001 g, 1.69 µmol) in C_6D_6 (0.2 ml) was heated to 100 ºC for 24 h under a N_2 atmosphere. After cooling the volatiles were removed in vacuo. The resulting off-white solid was re-dissolved in $CHCl_3$ (20.0 ml) and was filtered through a plug of silica. The filtrates volatiles were removed in vacuo to reveal white powdered solid. This was washed with H_2O (20.0 ml). Yield: 0.05 g, 90 %. ^{1}H NMR (499.91 MHz, $CDCl_3$): δ = 6.97 (m, 4H, Ph), 6.85 (m, 2H, *p*-Ph), 6.62 (dd, $^3J_{HH}$ = 8.2, 1.4 Hz, 4H, Ph), 0.13 (s, 18H, $SiMe_3$).

Spectroscopic Data

[Pd(ITMe)(methallyl)Cl] (2)

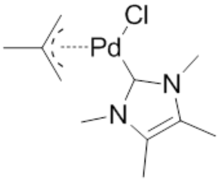

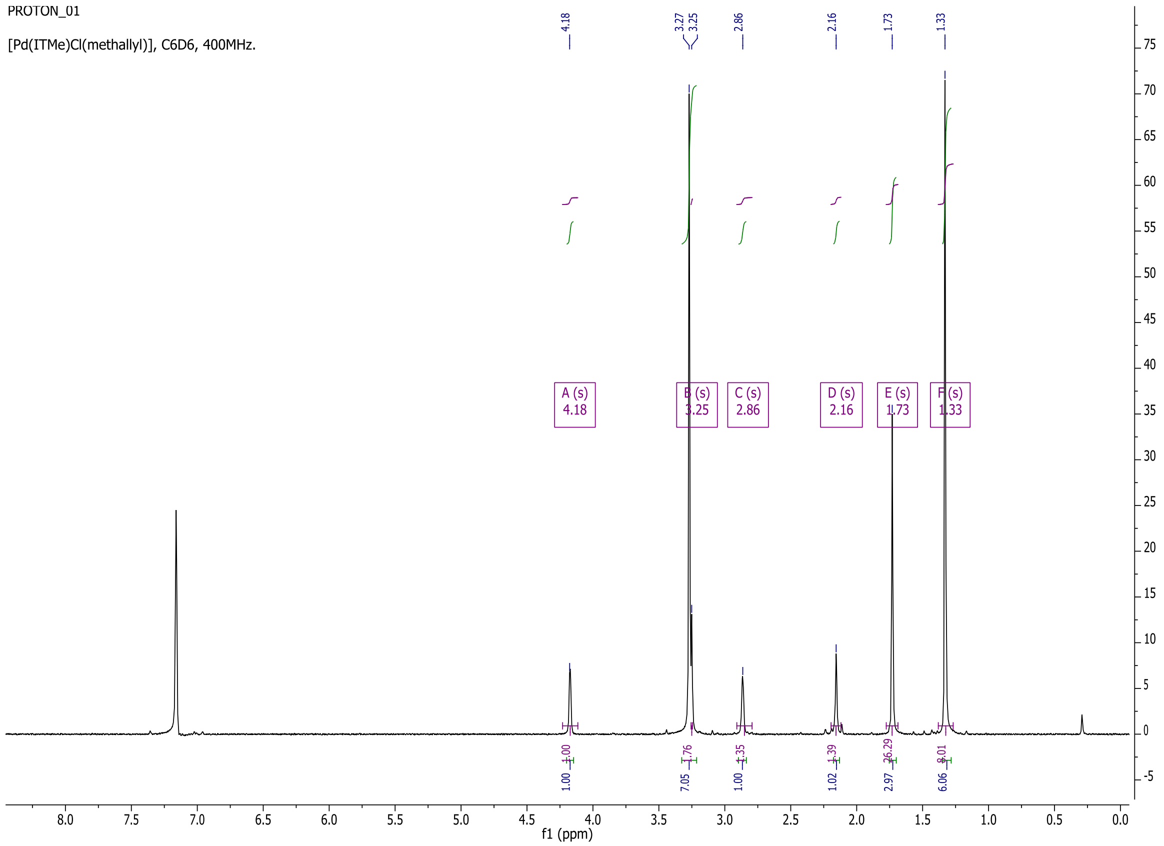

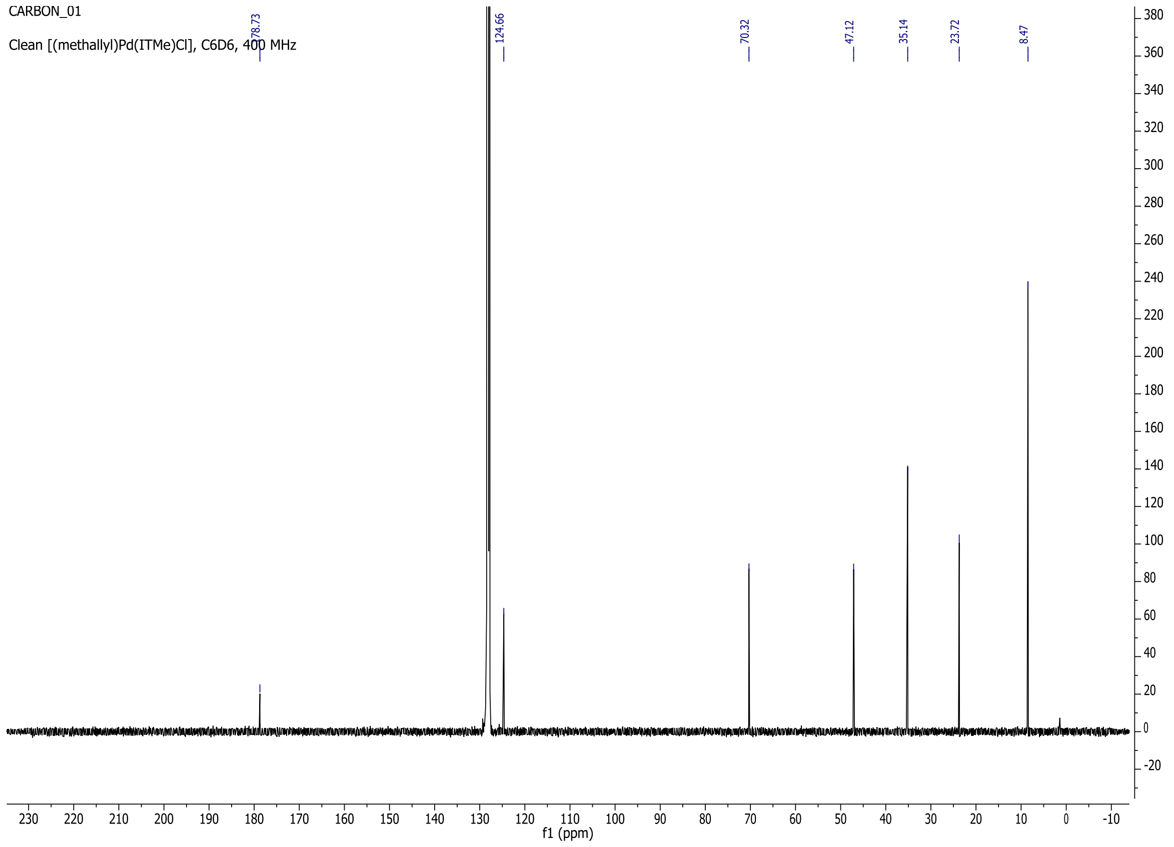
CARBON_01
Clean [(methallyl)Pd(ITMe)Cl], C6D6, 400 MHz
178.73
124.66
70.32
47.12
35.14
23.72
8.47
f1 (ppm)

cis-[Pd(ITMe)$_2$(SiMe$_3$)$_2$] (4)

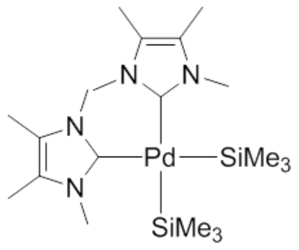

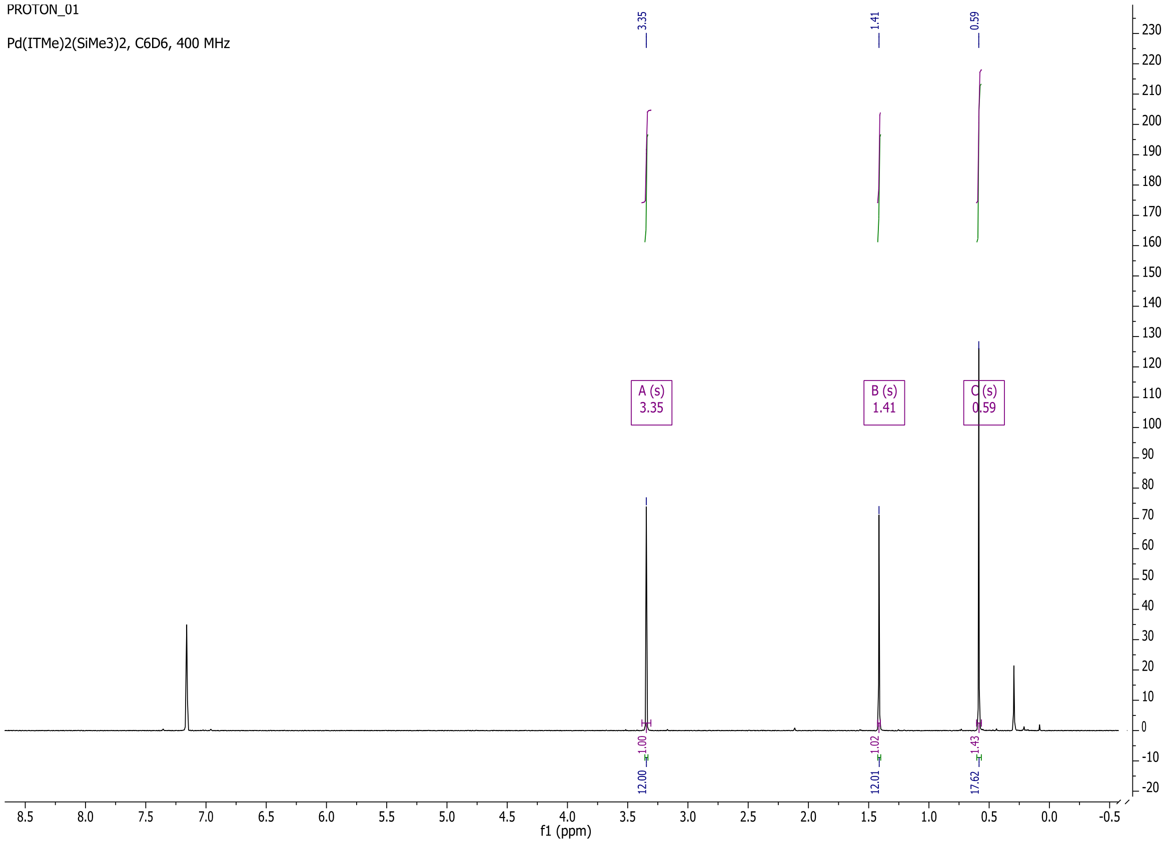

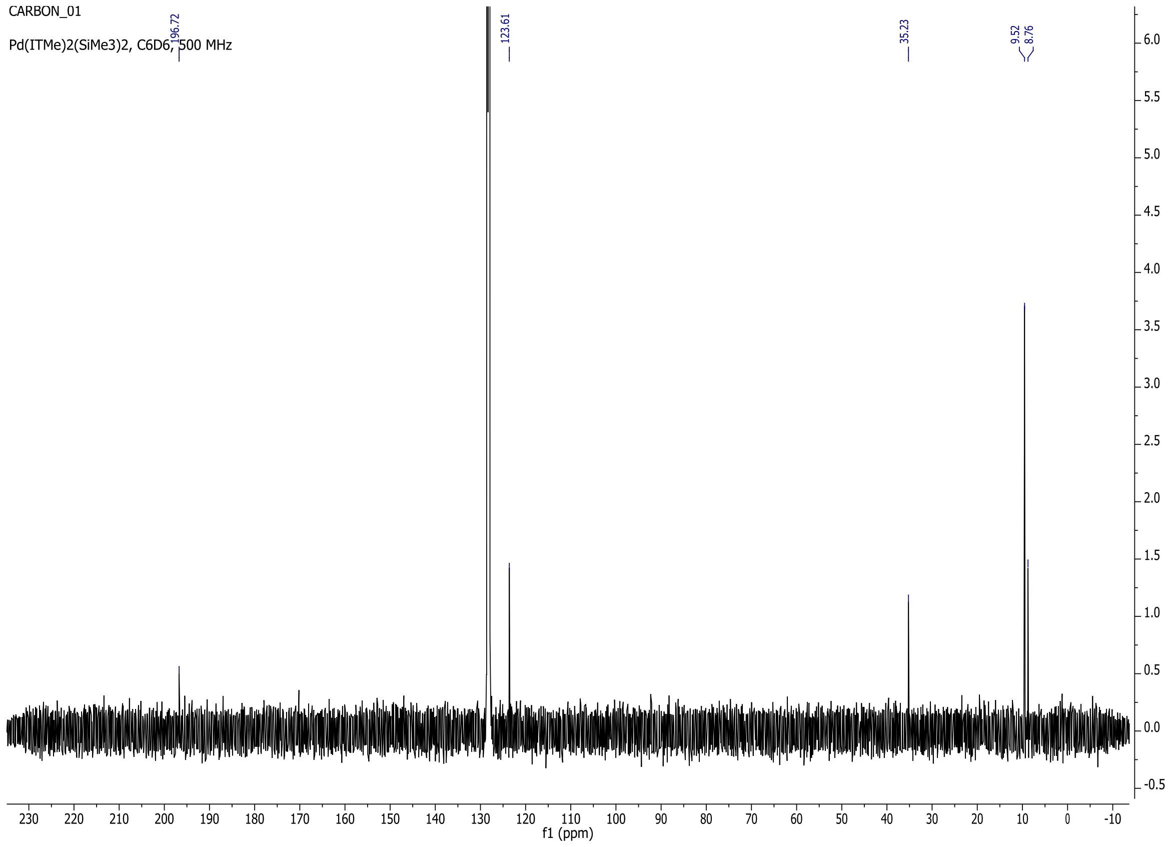
CARBON_01
Pd(ITMe)2(SiMe3)2, C6D6, 500 MHz
196.72
123.61
35.23
9.52
8.76
f1 (ppm)

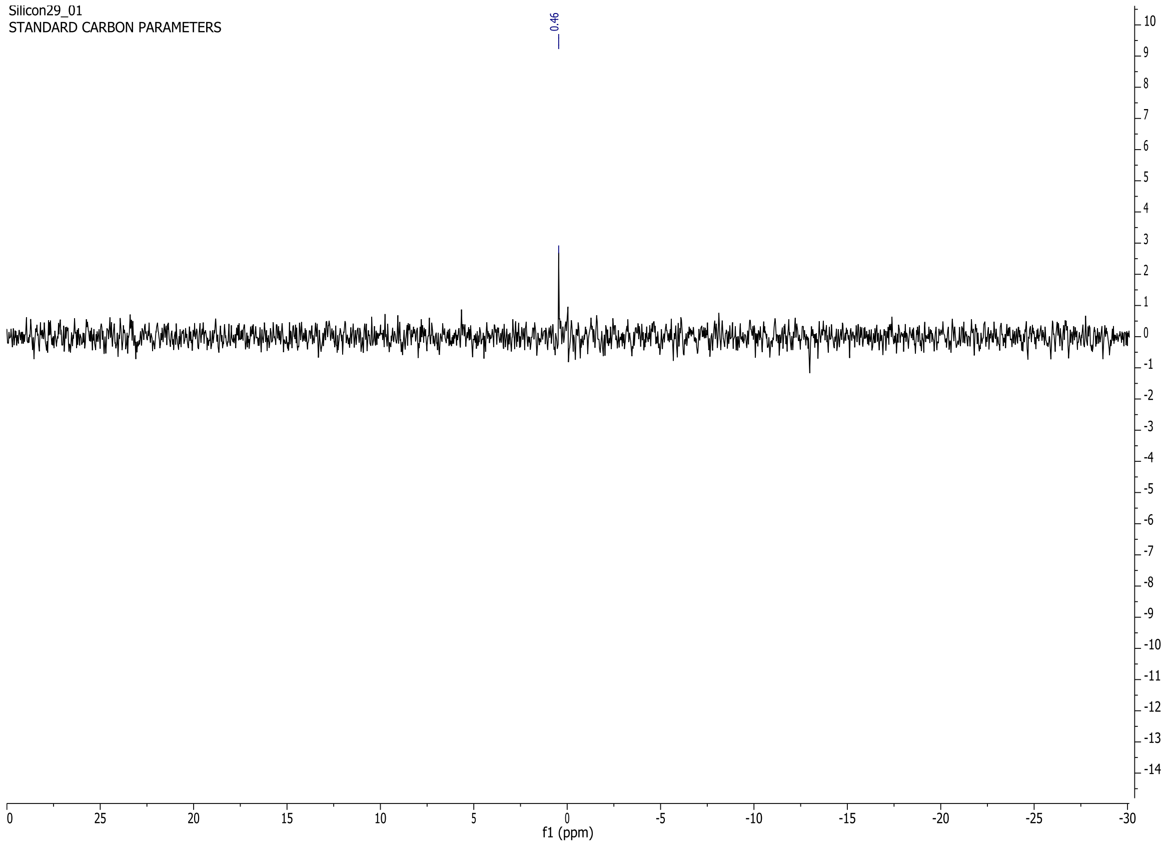
Silicon29_01
STANDARD CARBON PARAMETERS
0.46
f1 (ppm)

Heating of *cis*-[Pd(ITMe)$_2$(SiMe$_3$)$_2$]

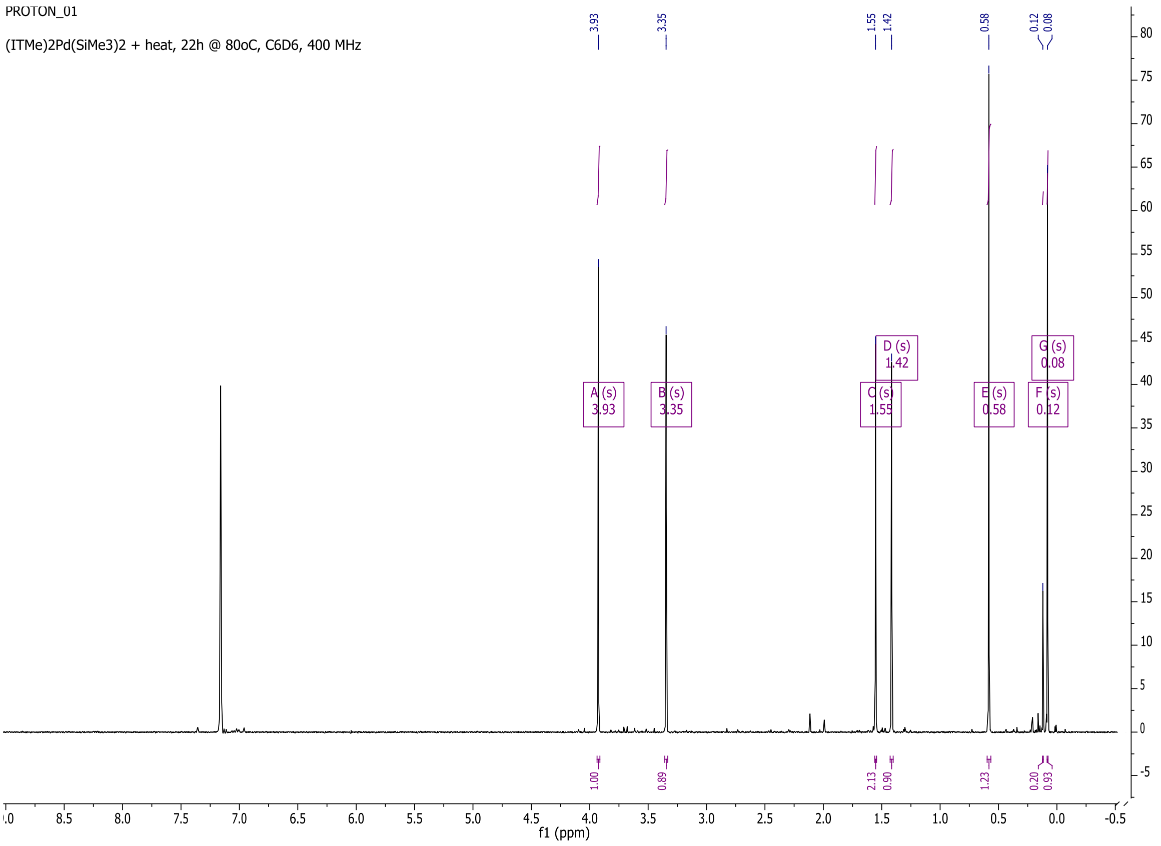

Pd(ITMe)$_2$(PhCCPh) (6)

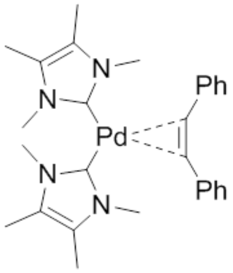

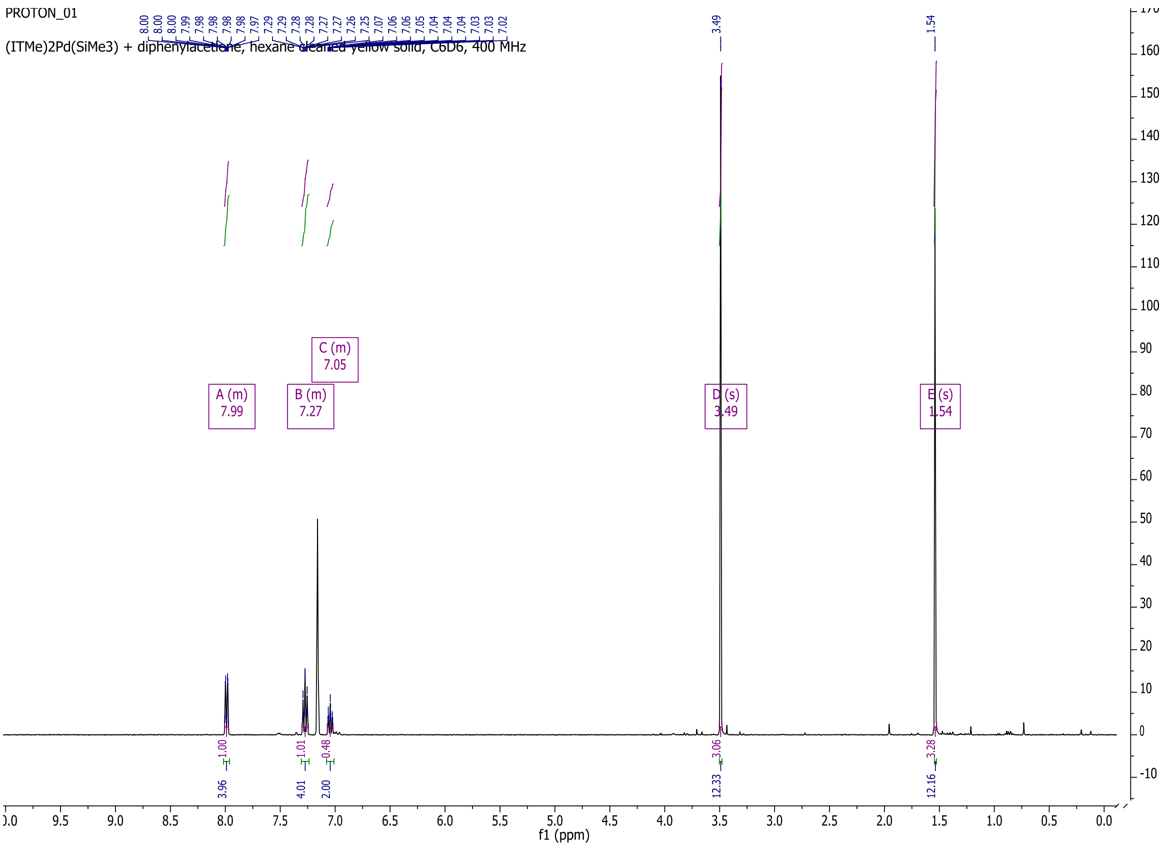

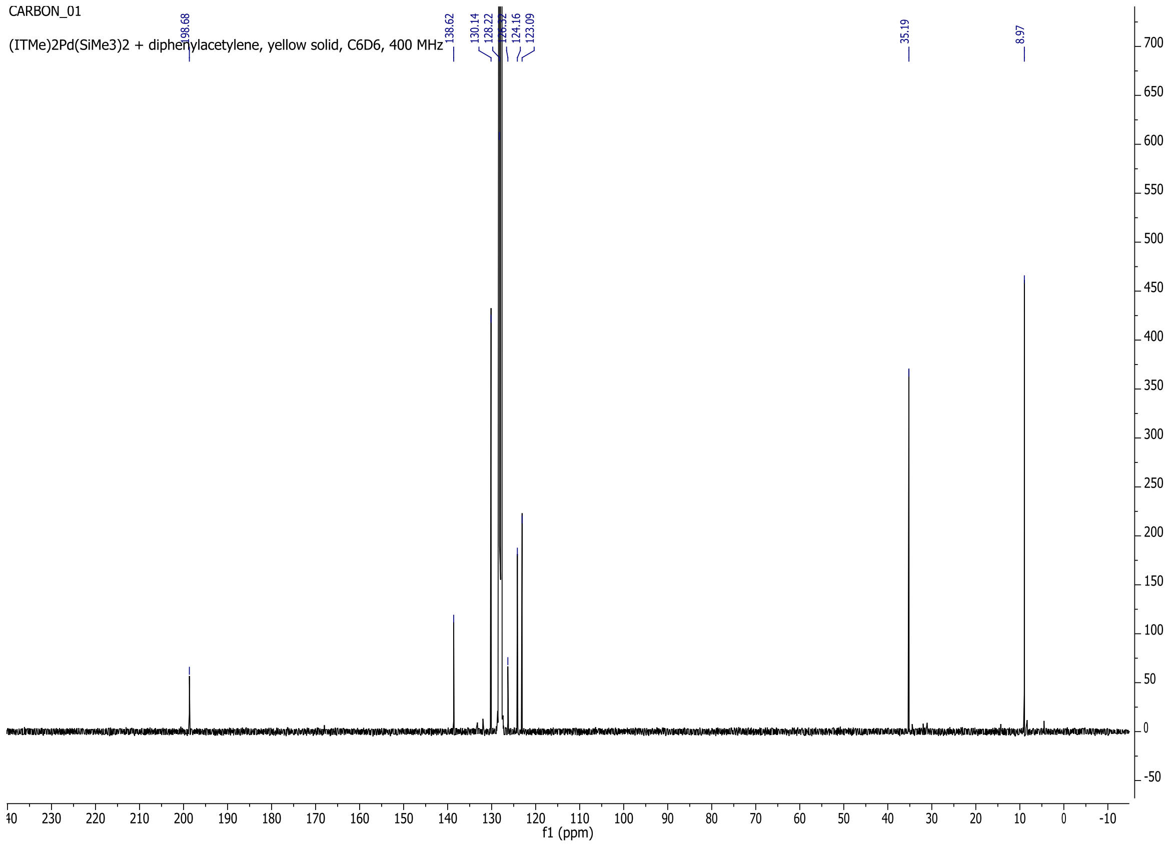
CARBON_01
(ITMe)2Pd(SiMe3)2 + diphenylacetylene, yellow solid, C6D6, 400 MHz
198.68
138.62
130.14
128.22
126.52
124.16
123.09
35.19
8.97
f1 (ppm)

cis-(Me_3Si)(Ph)C=C(Ph)($SiMe_3$) (5)

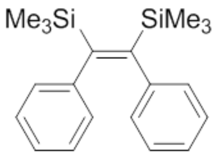

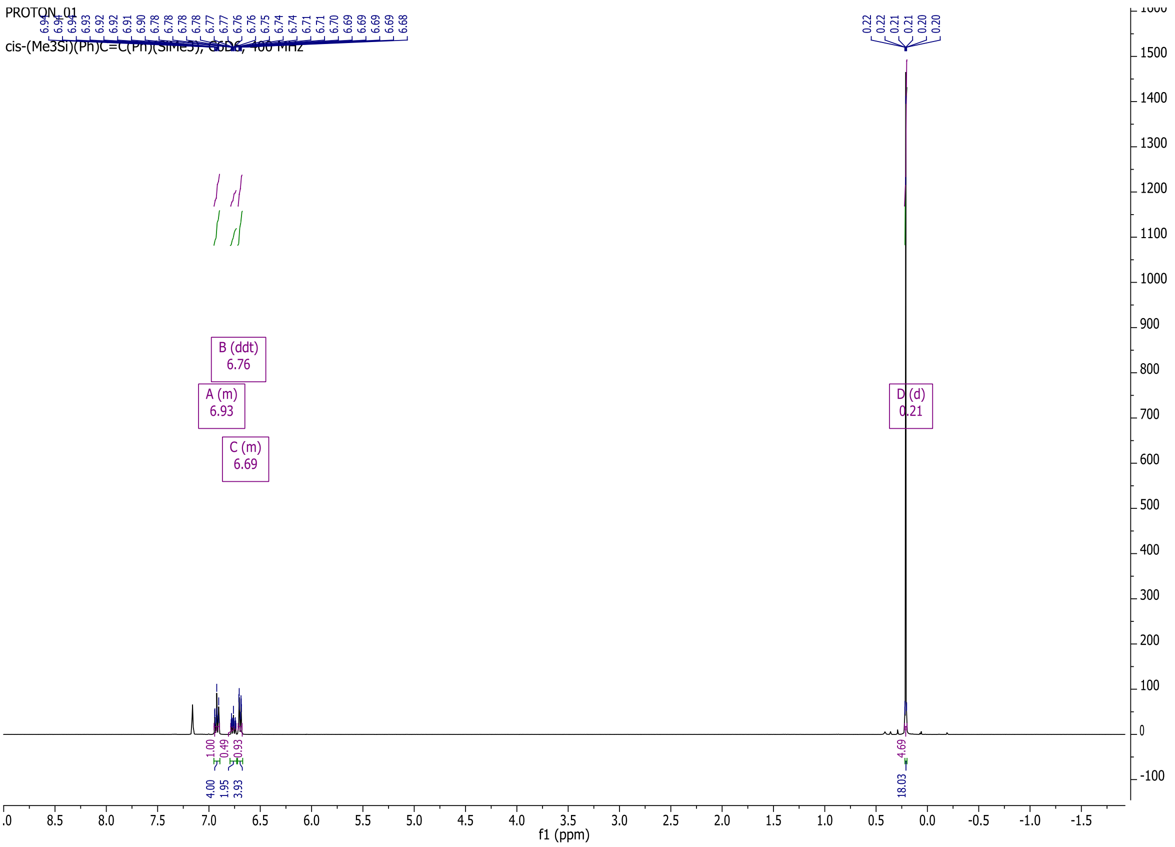

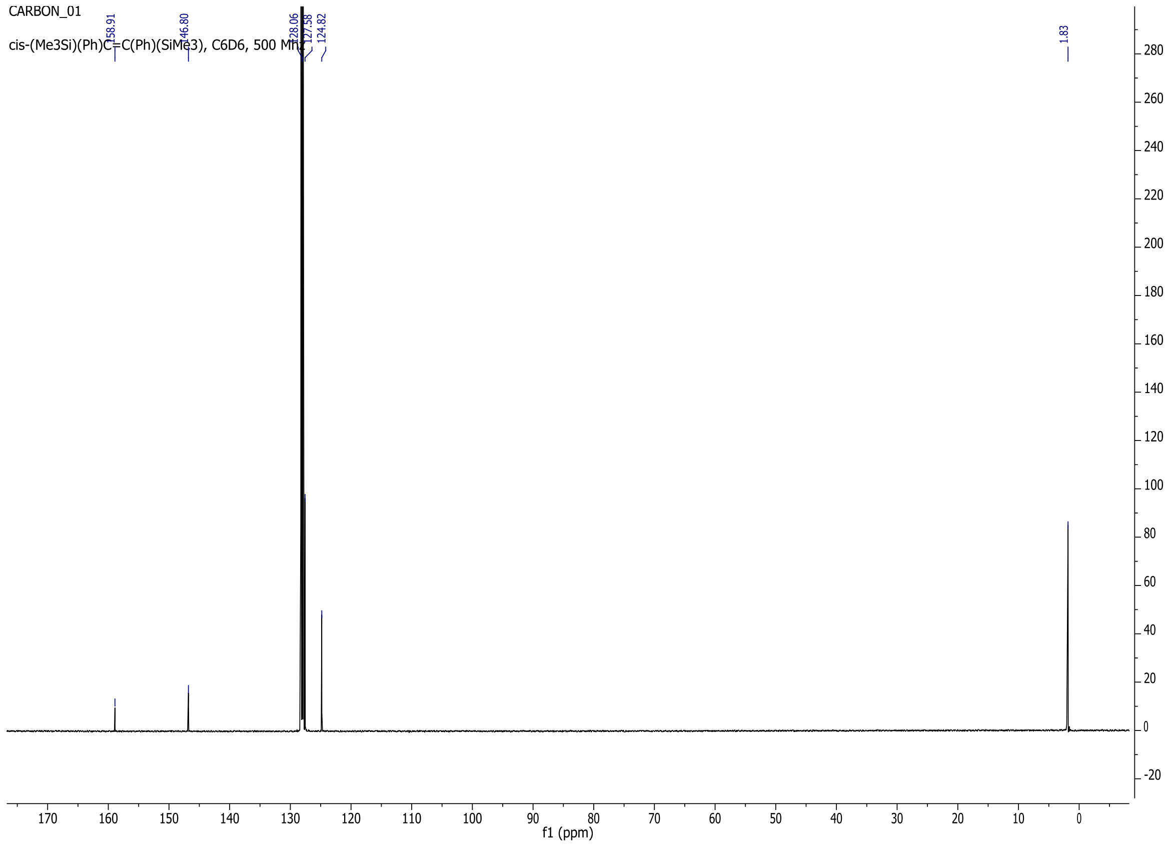
CARBON_01
cis-(Me3Si)(Ph)C=C(Ph)(SiMe3), C6D6, 500 MHz
158.91
146.80
128.06
127.58
124.82
1.83
f1 (ppm)

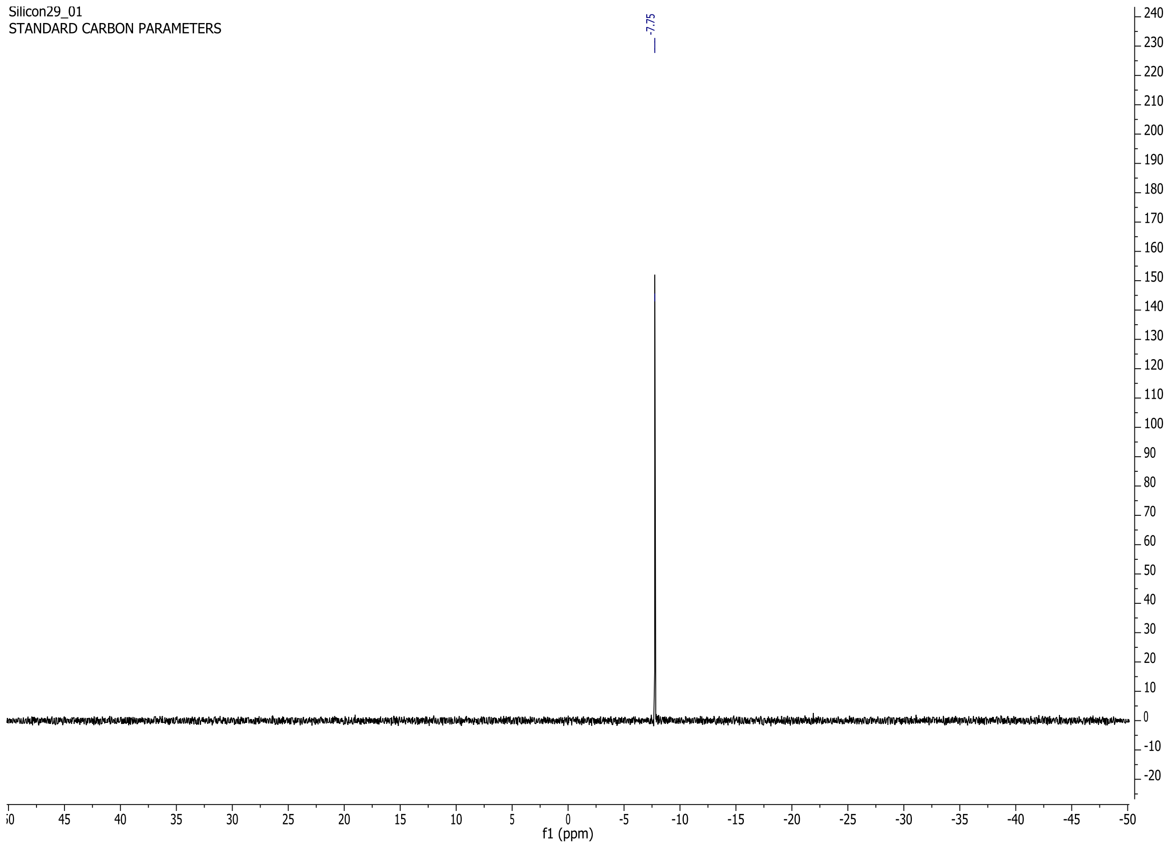
Silicon29_01
STANDARD CARBON PARAMETERS
-7.75
f1 (ppm)

MBA1158 MW=324?
ASAP (SOLID + NH4OAc)

EPSRC National Facility Swansea
LTQ Orbitrap XL

Melvyn
28/01/2015 02:19:35 PM

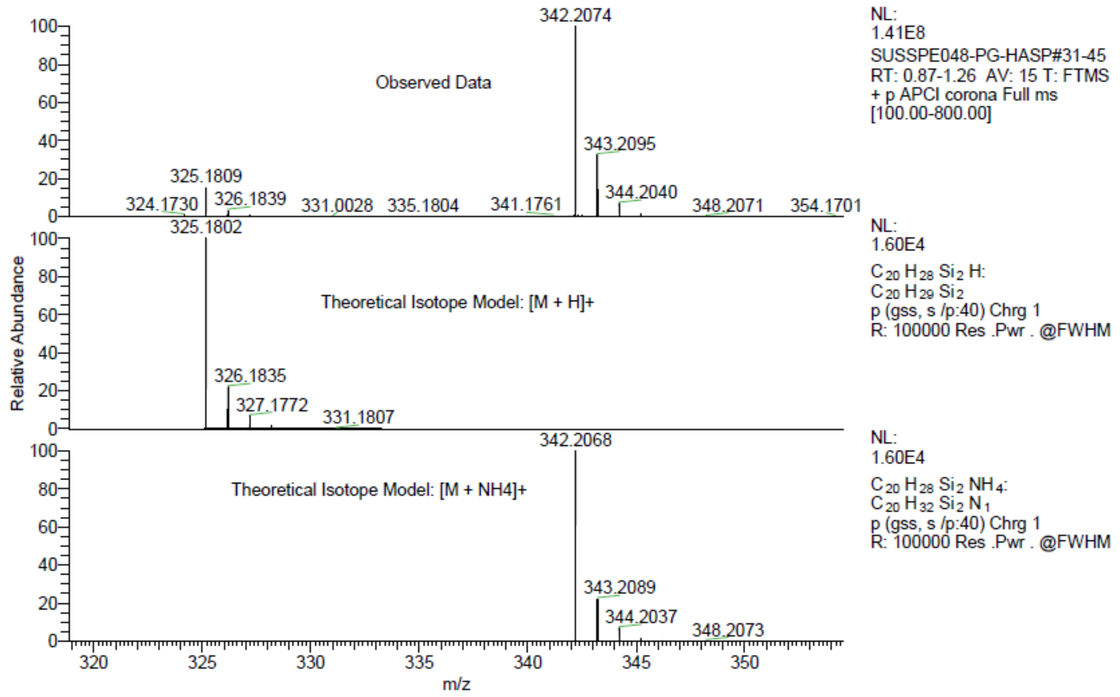

cis-(Ph)($SiMe_2Ph$)C=C($SiMe_2Ph$)(Ph) (7)

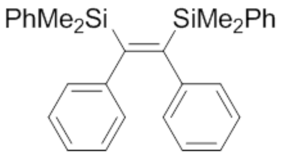

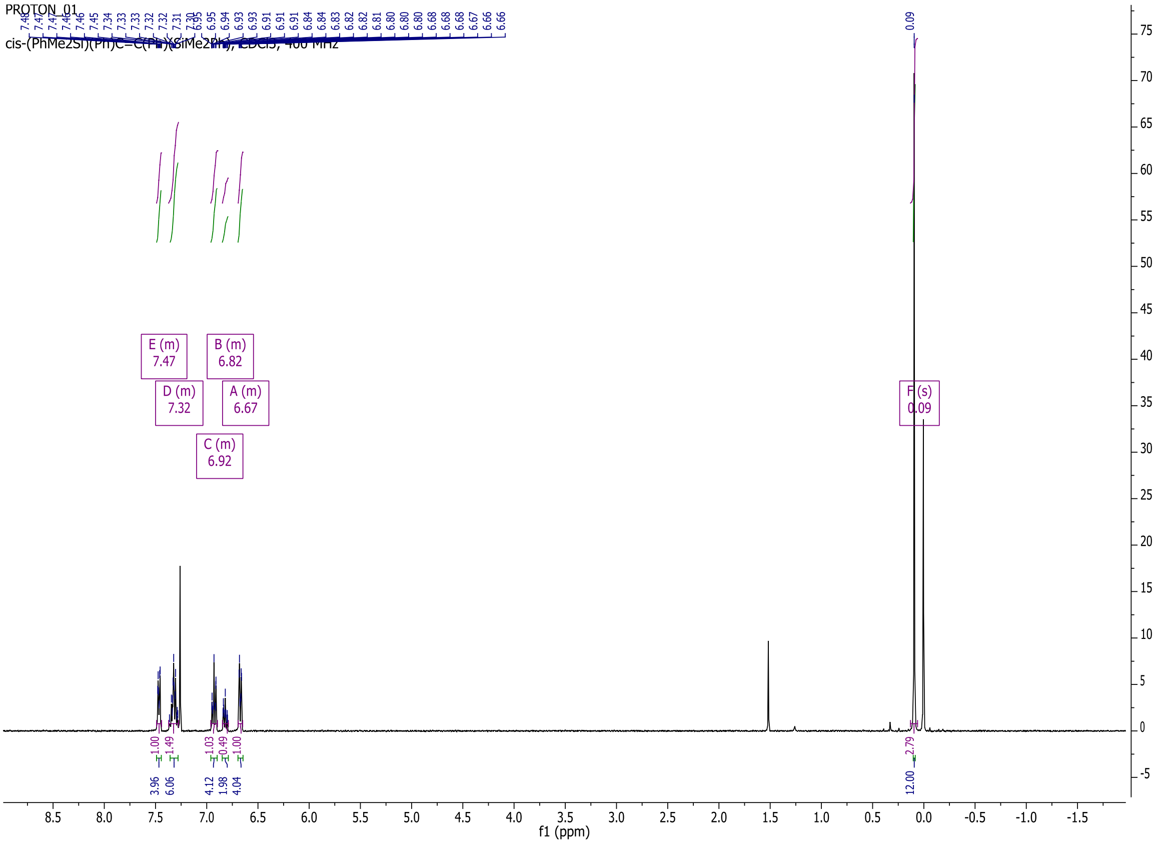

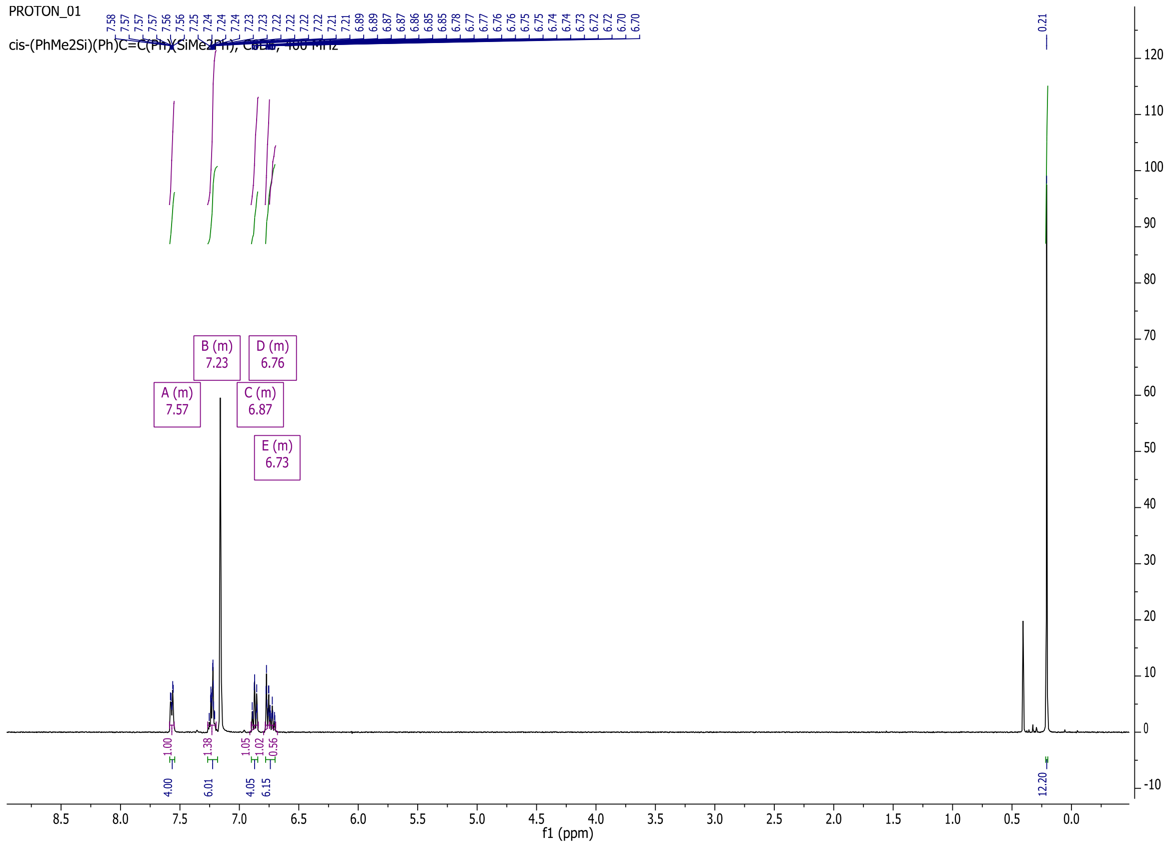
PROTON_01
7.58
7.57
7.57
7.57
7.56
7.56
7.25
7.24
7.24
7.24
7.23
7.23
7.22
7.22
7.22
7.22
7.22
7.21
7.21
6.89
6.87
6.87
6.86
6.85
6.85
6.78
6.77
6.77
6.76
6.76
6.75
6.75
6.74
6.74
6.73
6.72
6.72
6.70
6.70
0.21
A (m)
7.57
B (m)
7.23
C (m)
6.87
D (m)
6.76
E (m)
6.73
1.00
1.38
1.05
1.02
0.56
4.00
6.01
4.05
6.15
12.20
f1 (ppm)

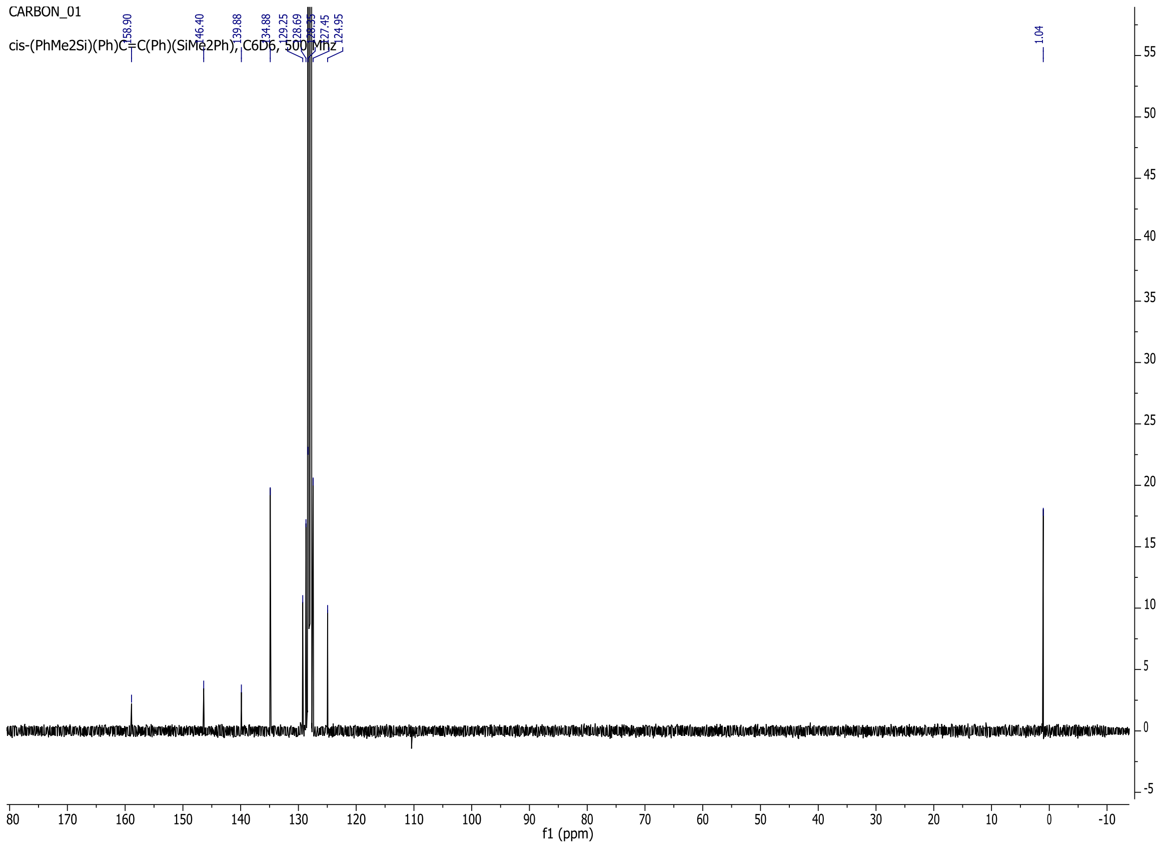
CARBON_01
cis-(PhMe2Si)(Ph)C=C(Ph)(SiMe2Ph), C6D6, 500 MHz
158.90
146.40
139.88
134.88
129.25
128.69
127.45
124.95
1.04
f1 (ppm)

cis-(Ph)(Me_3Si)C=C($SiMe_3$) (*p*-$C_6H_4C(O)CH_3$)($SiMe_3$) (8)

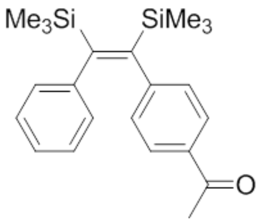

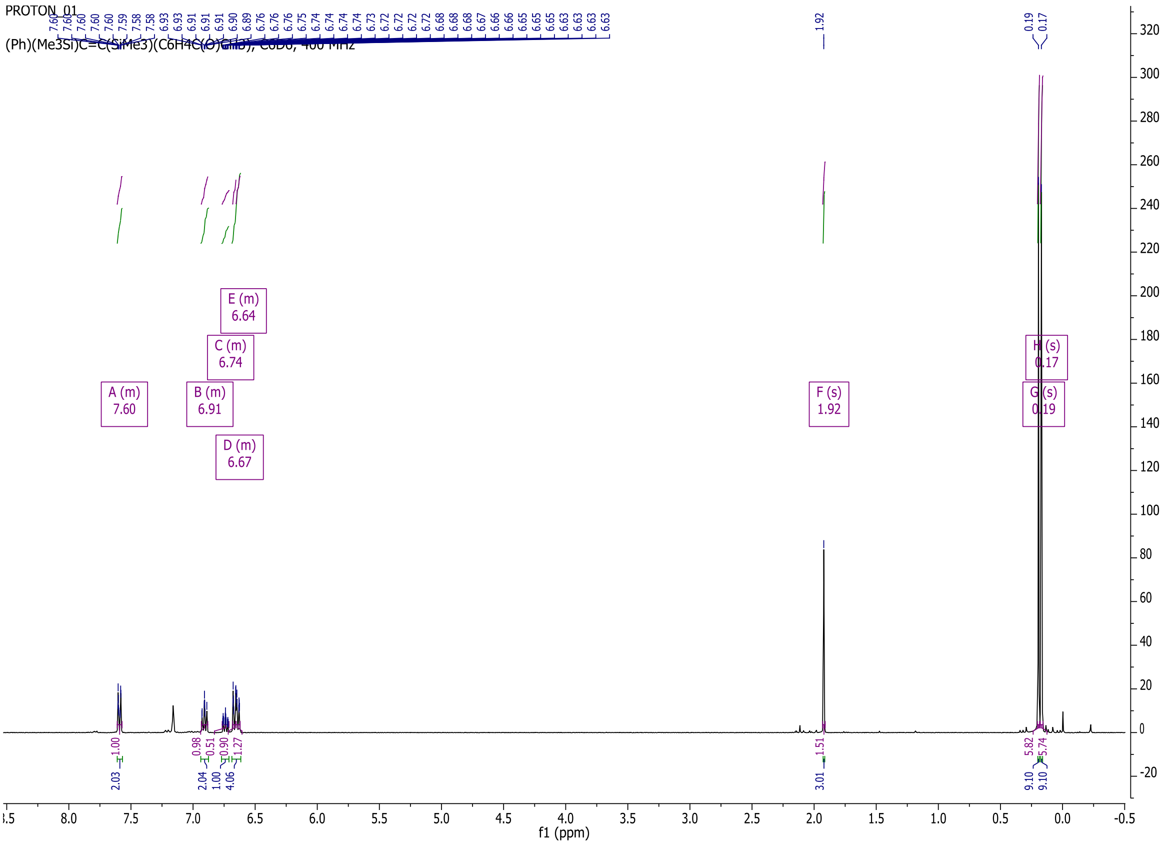

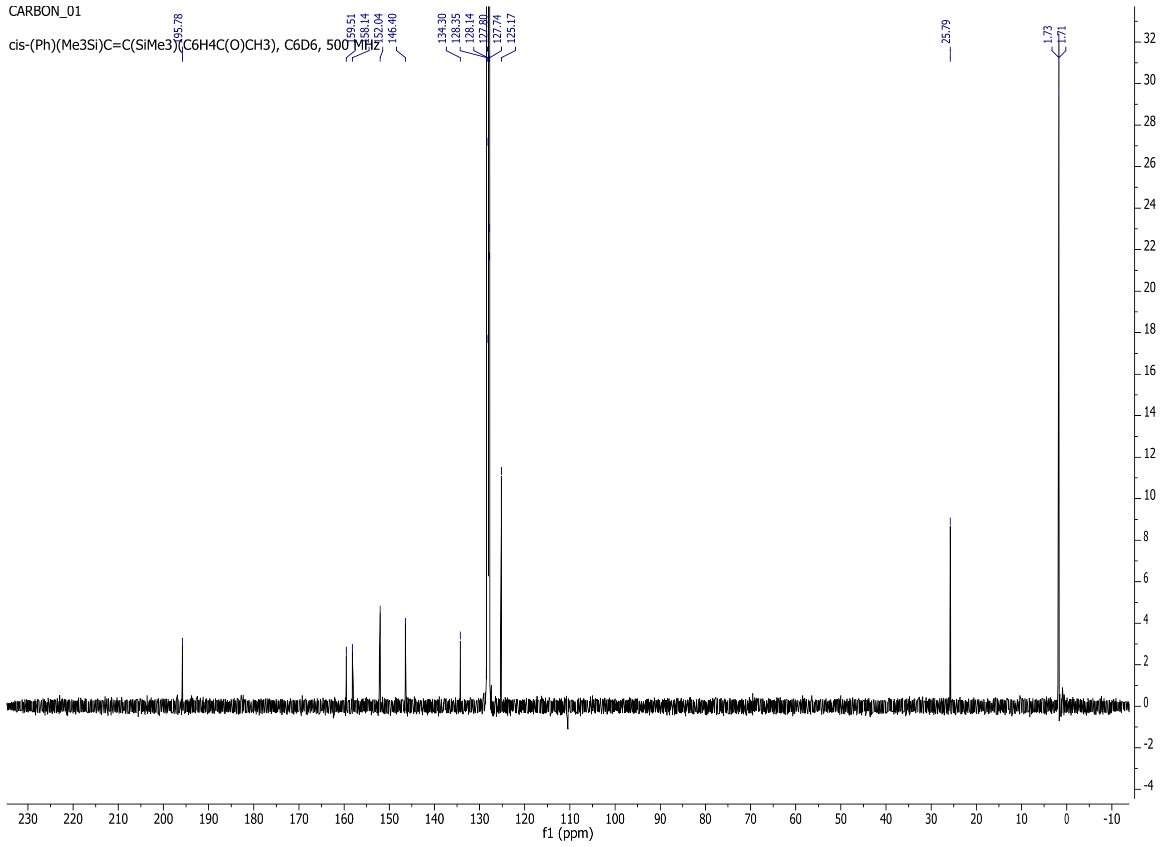
CARBON_01
cis-(Ph)(Me3Si)C=C(SiMe3)(C6H4C(O)CH3), C6D6, 500 MHz
195.78
159.51
158.14
152.04
146.40
134.30
128.35
128.14
127.80
127.74
125.17
25.79
1.73
1.71
f1 (ppm)

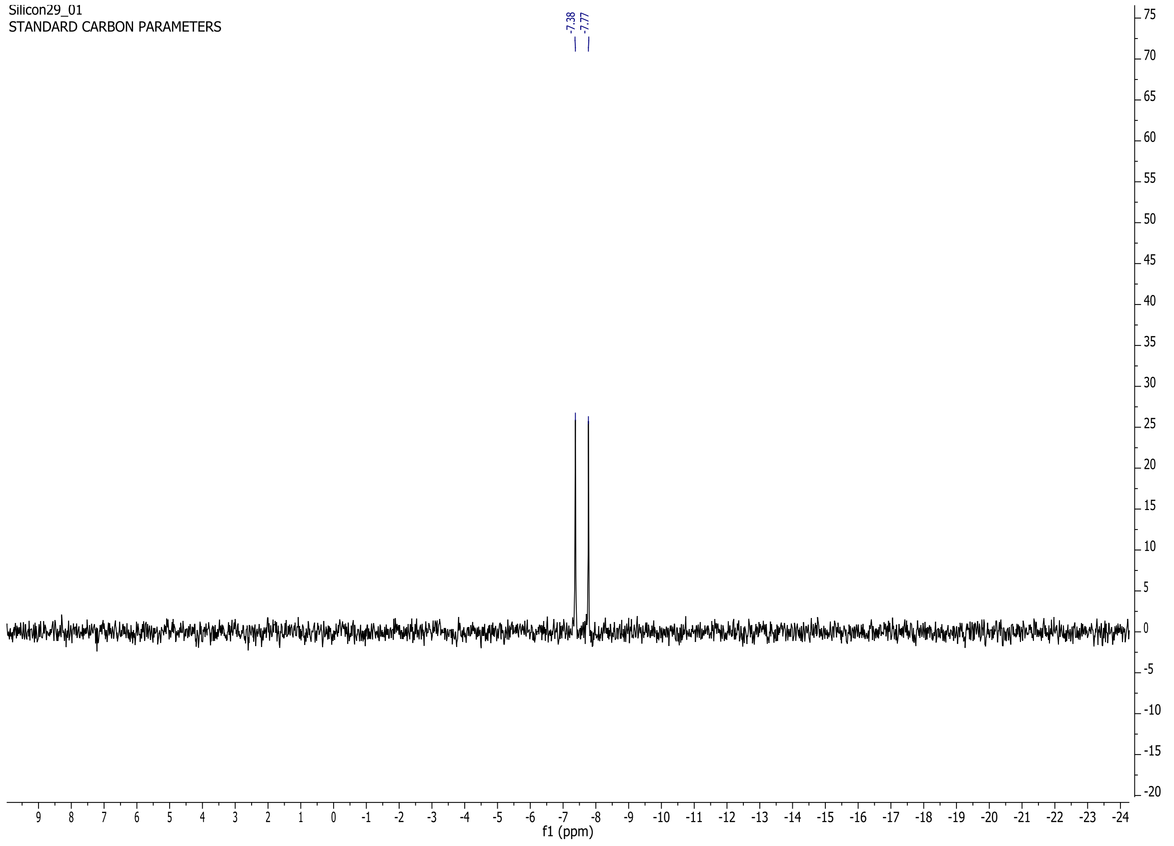
Silicon29_01
STANDARD CARBON PARAMETERS
-7.38
-7.77
f1 (ppm)

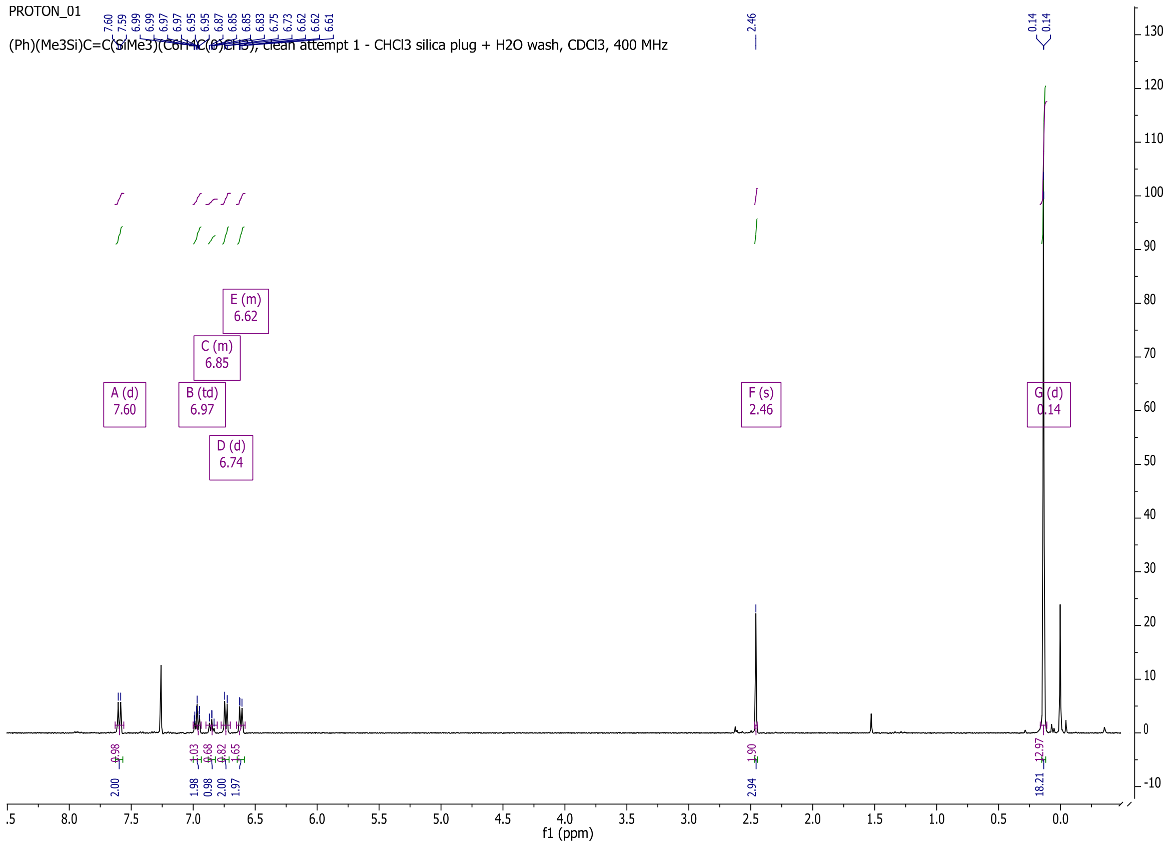
PROTON_01
(Ph)(Me3Si)C=C(SiMe3)(C6H4C(O)CH3), clean attempt 1 - CHCl3 silica plug + H2O wash, CDCl3, 400 MHz
A (d) 7.60
B (td) 6.97
C (m) 6.85
D (d) 6.74
E (m) 6.62
F (s) 2.46
G (d) 0.14
f1 (ppm)

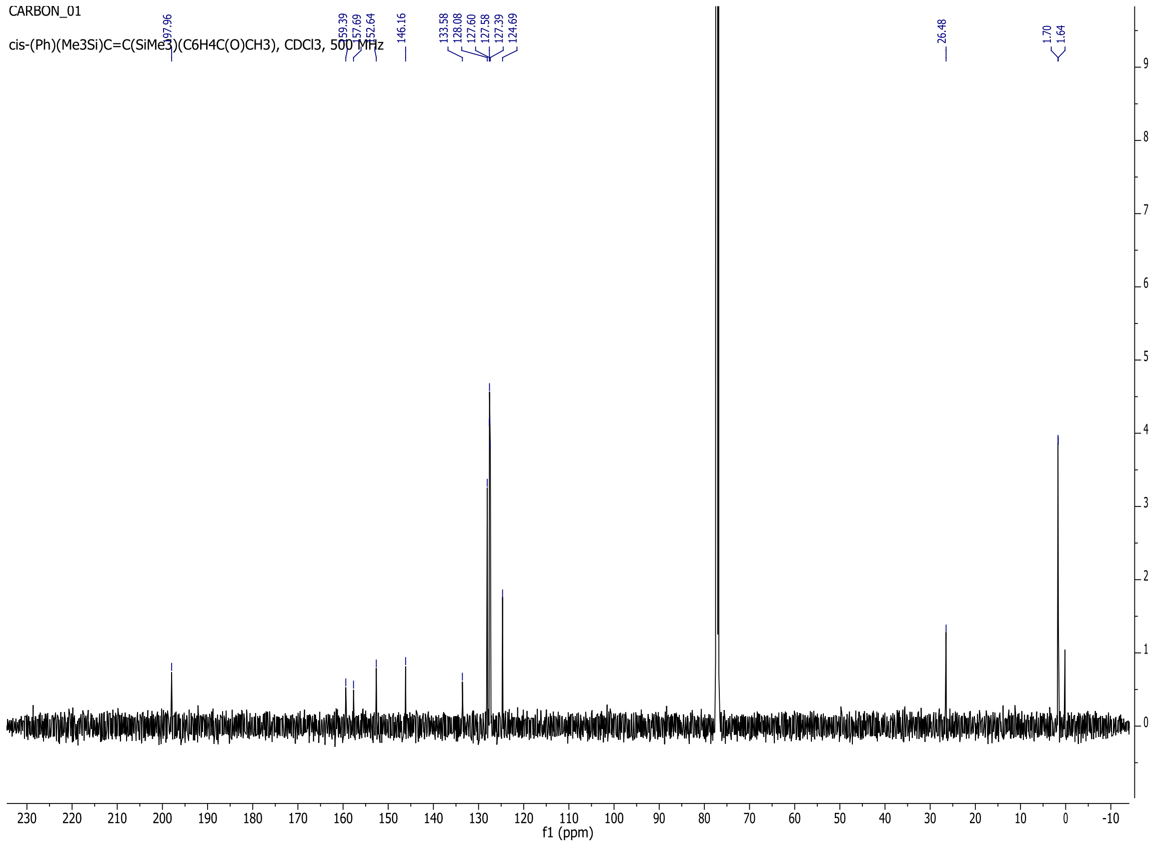
CARBON_01
cis-(Ph)(Me3Si)C=C(SiMe3)(C6H4C(O)CH3), CDCl3, 500 MHz
197.96
159.39
157.69
152.64
146.16
133.58
128.08
127.60
127.58
127.39
124.69
26.48
1.70
1.64
f1 (ppm)

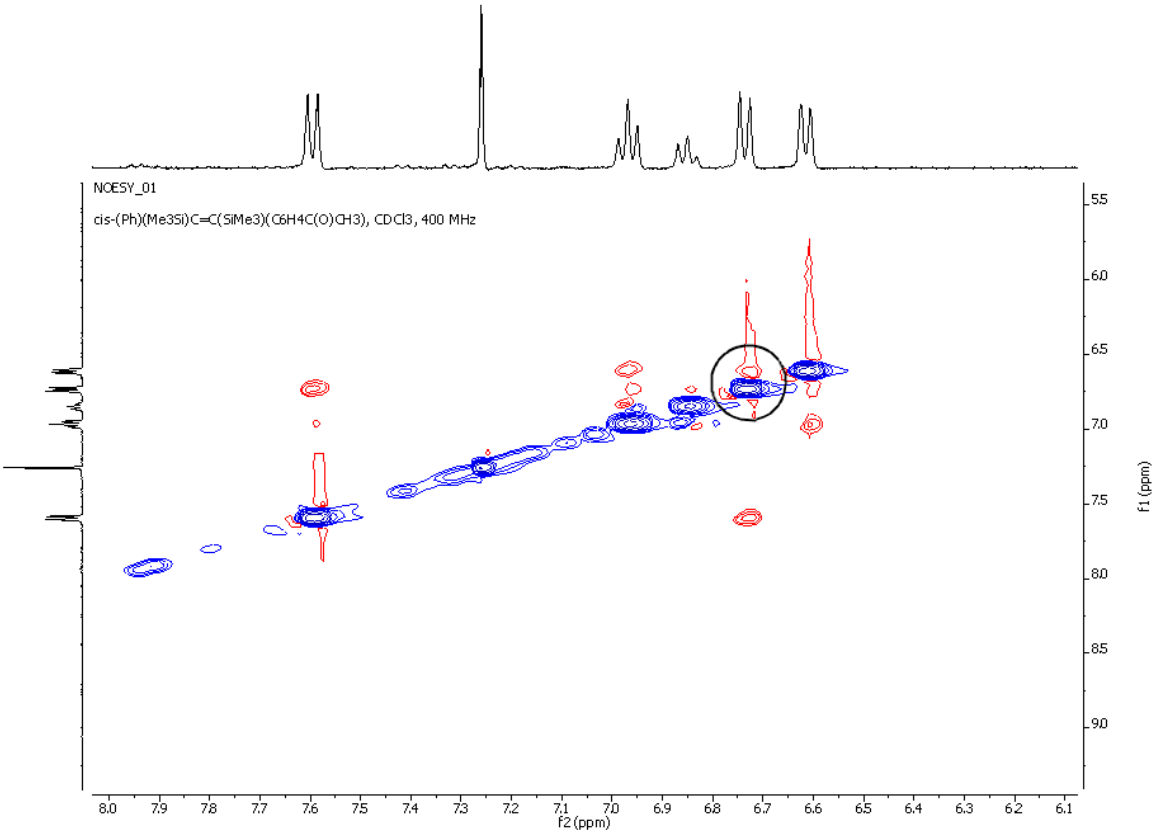
NOESY_01
cis-(Ph)(Me3Si)C=C(SiMe3)(C6H4C(O)CH3), CDCl3, 400 MHz
f2 (ppm)
f1 (ppm)

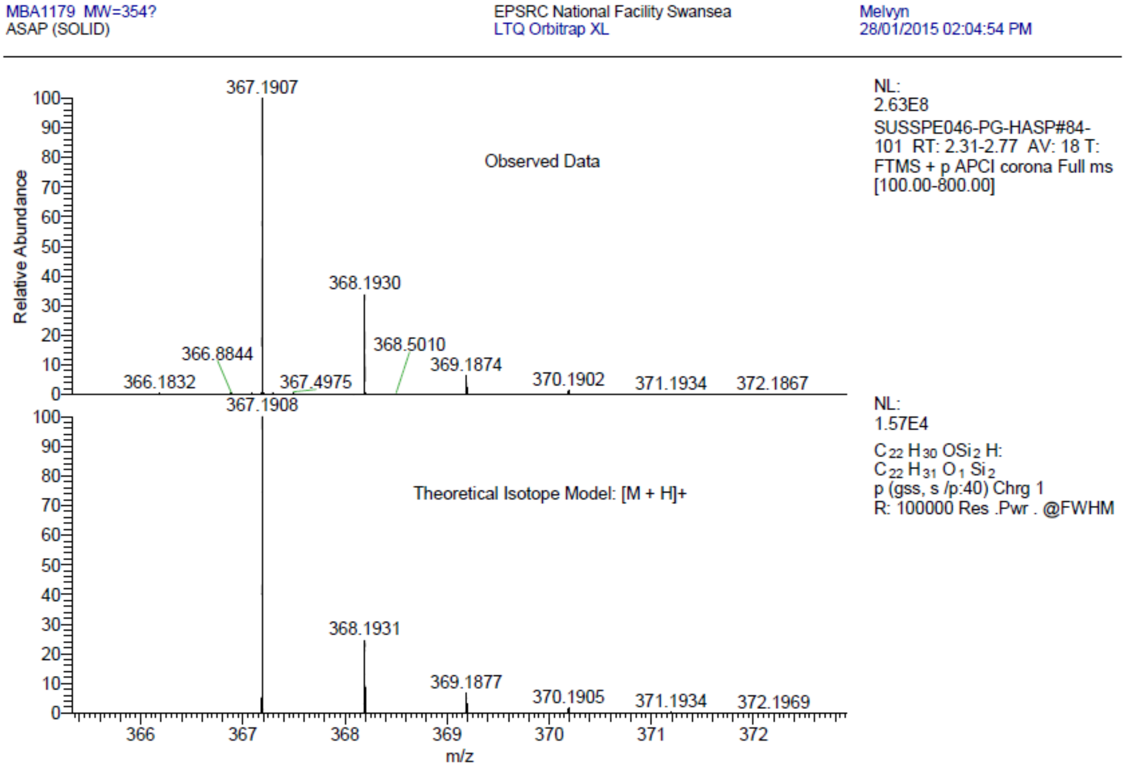
MBA1179 MW=354?
ASAP (SOLID)
EPSRC National Facility Swansea
LTQ Orbitrap XL
Melvyn
28/01/2015 02:04:54 PM
Observed Data
NL:
2.63E8
SUSSPE046-PG-HASP#84-101 RT: 2.31-2.77 AV: 18 T: FTMS + p APCI corona Full ms [100.00-800.00]
367.1907
368.1930
366.8844
368.5010
369.1874
366.1832
367.4975
370.1902
371.1934
372.1867
Theoretical Isotope Model: [M + H]+
NL:
1.57E4
C22 H30 OSi2 H:
C22 H31 O1 Si2
p (gss, s /p:40) Chrg 1
R: 100000 Res .Pwr . @FWHM
367.1908
368.1931
369.1877
370.1905
371.1934
372.1969
Relative Abundance
m/z

***cis*-(Ph)($SiMe_3$)C=C($SiMe_3$)(*p*-Tol) (9)**

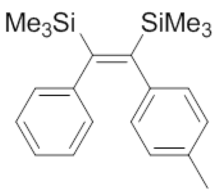

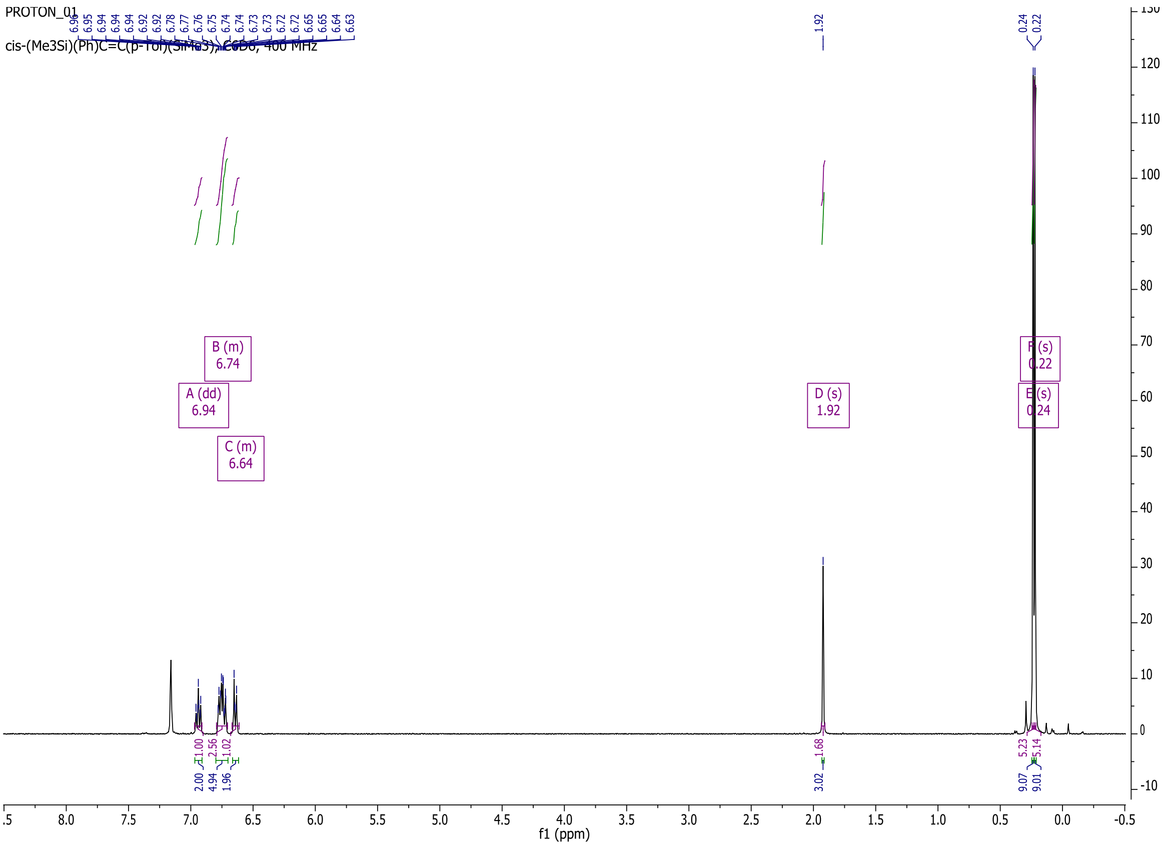

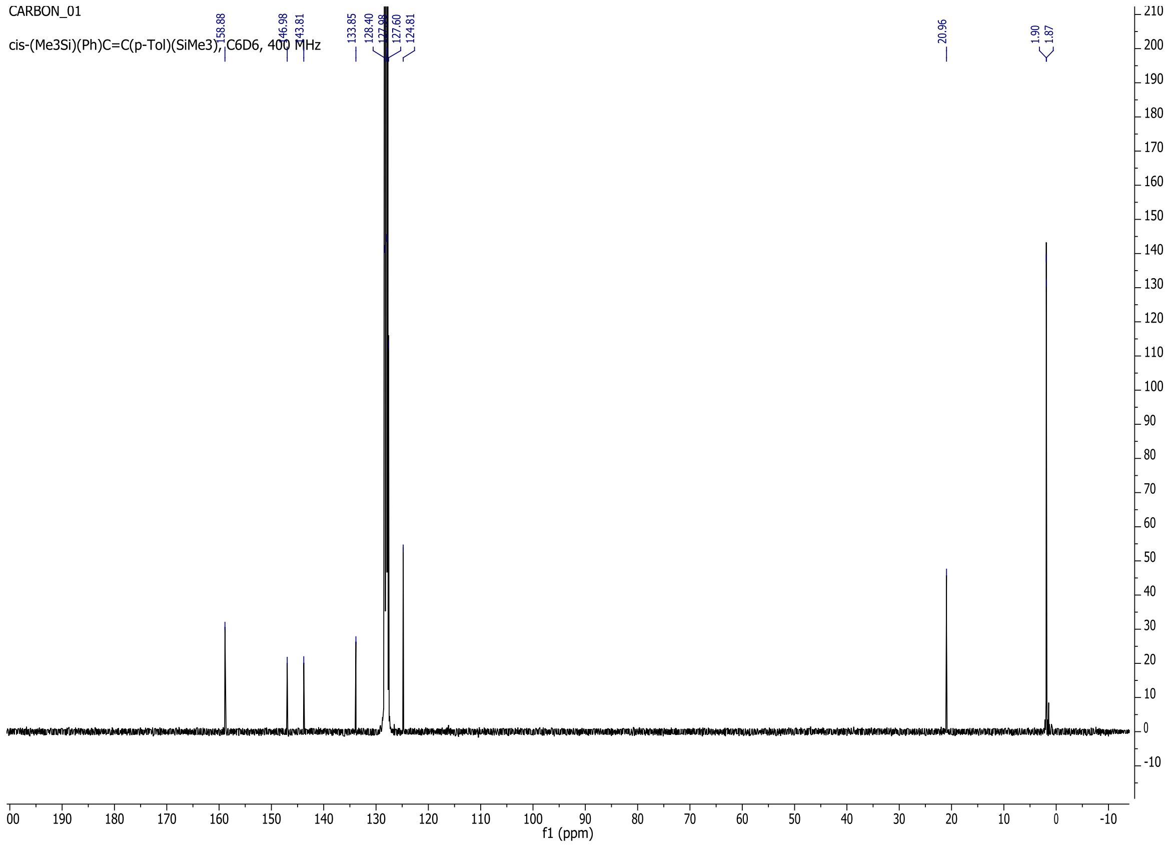
CARBON_01
cis-(Me3Si)(Ph)C=C(p-Tol)(SiMe3), C6D6, 400 MHz
158.88
146.98
143.81
133.85
128.40
127.98
127.60
124.81
20.96
1.90
1.87
f1 (ppm)

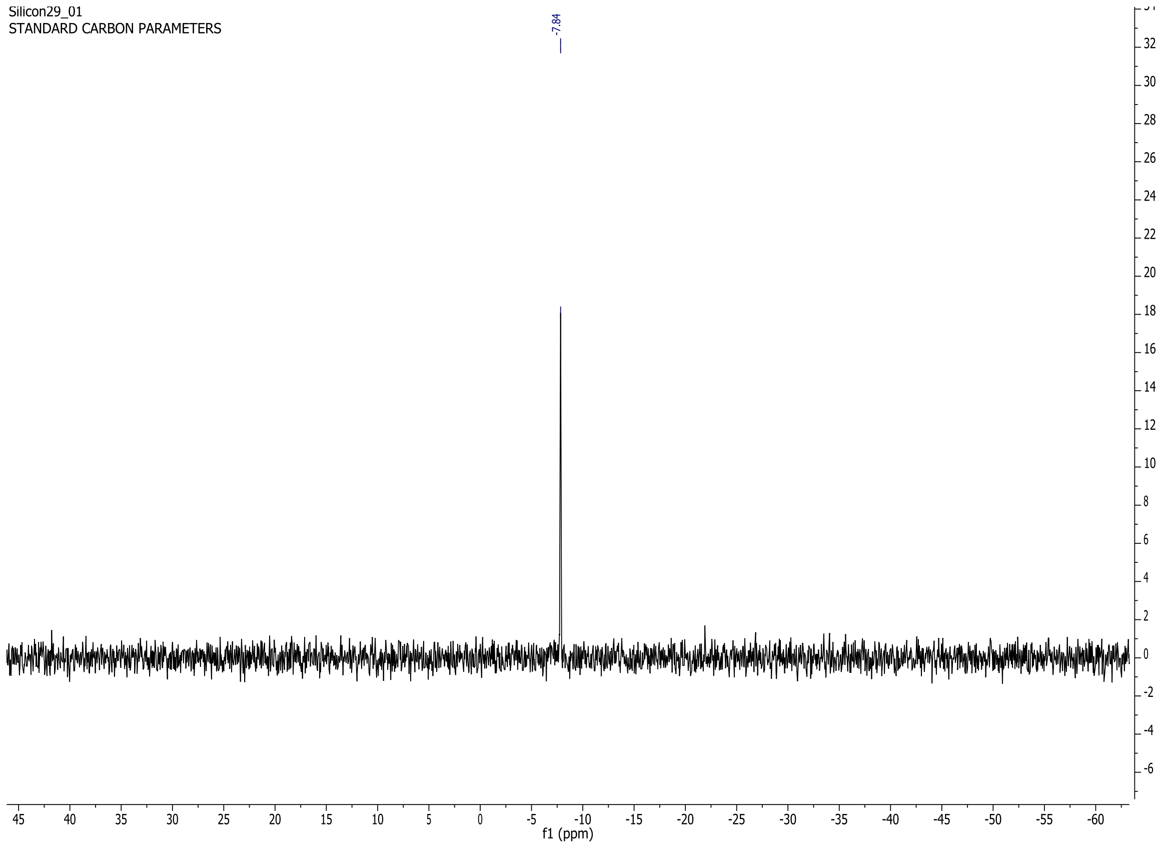
Silicon29_01
STANDARD CARBON PARAMETERS
-7.84
f1 (ppm)

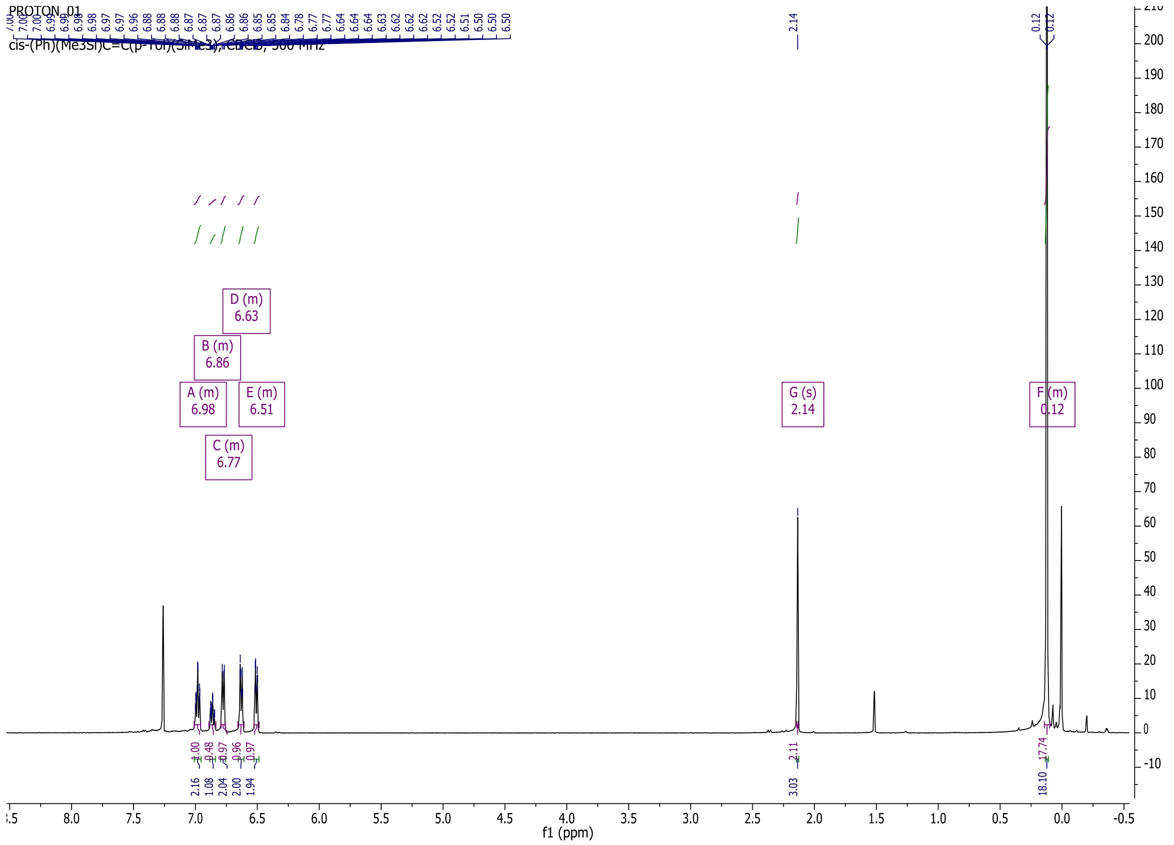
PROTON_01
cis-(Ph)(Me3Si)C=C(p-Tol)(SiMe3), CDCl3, 500 MHz
A (m)
6.98
B (m)
6.86
C (m)
6.77
D (m)
6.63
E (m)
6.51
F (m)
0.12
G (s)
2.14
f1 (ppm)

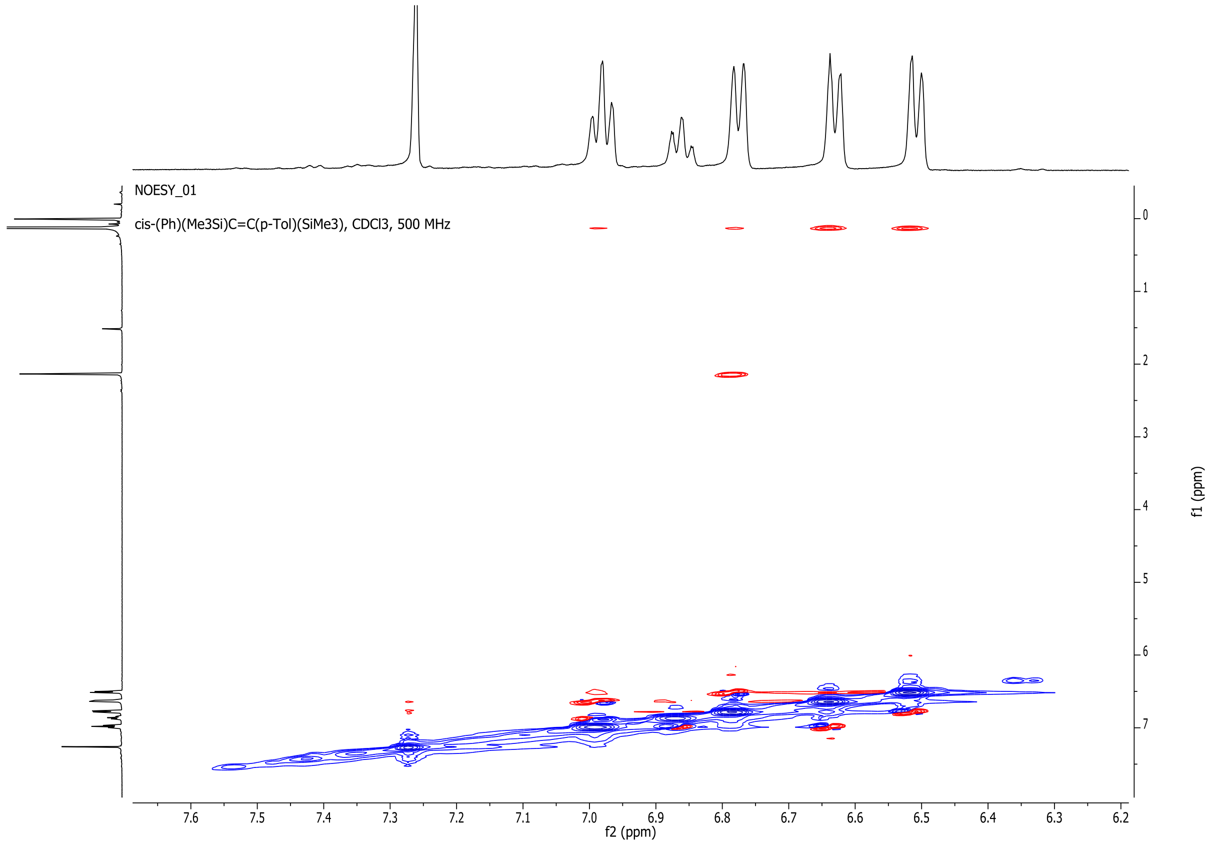
NOESY_01
cis-(Ph)(Me3Si)C=C(p-Tol)(SiMe3), CDCl3, 500 MHz
f2 (ppm)
f1 (ppm)

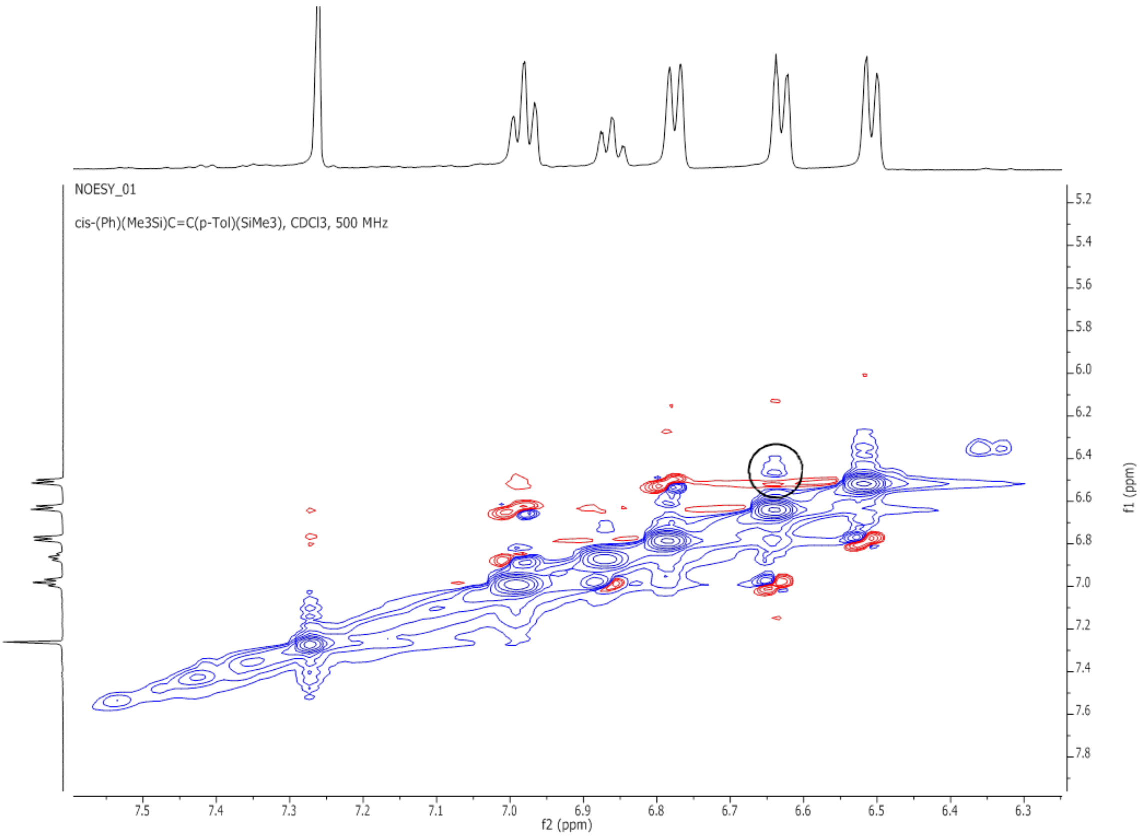
NOESY_01
cis-(Ph)(Me3Si)C=C(p-Tol)(SiMe3), CDCl3, 500 MHz
f2 (ppm)
f1 (ppm)

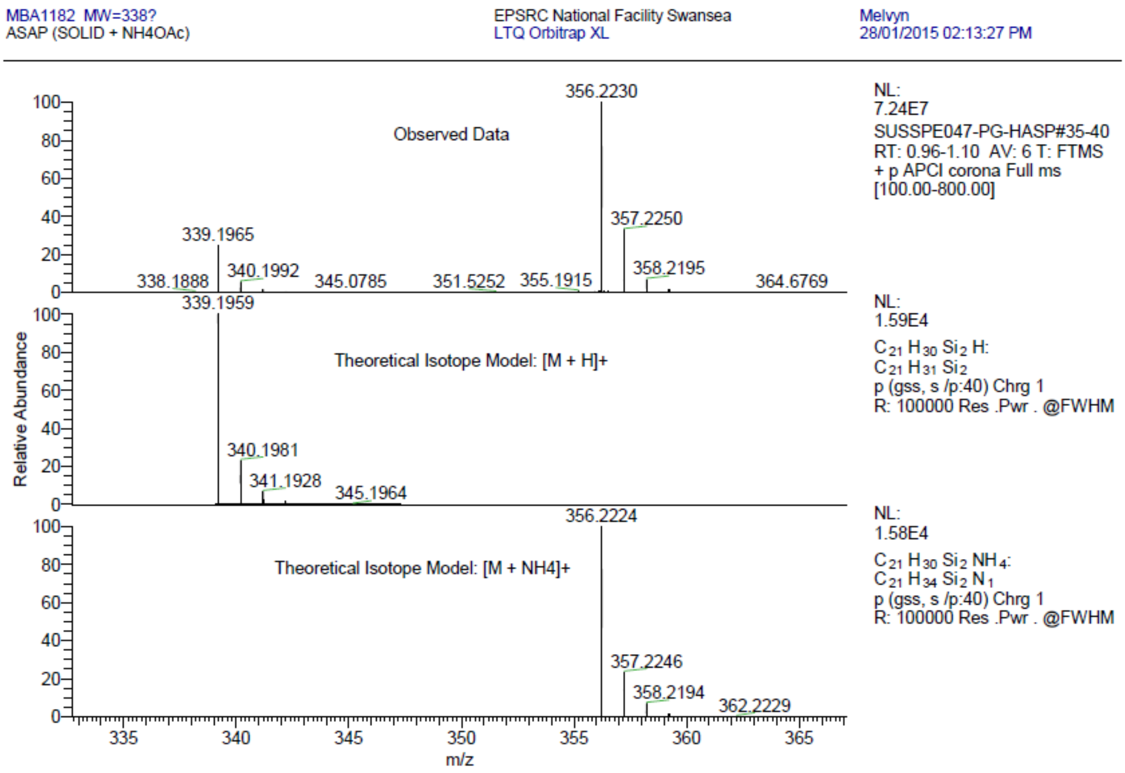
MBA1182 MW=338?
ASAP (SOLID + NH4OAc)
EPSRC National Facility Swansea
LTQ Orbitrap XL
Melvyn
28/01/2015 02:13:27 PM
Observed Data
356.2230
357.2250
339.1965
340.1992
338.1888
345.0785
351.5252
355.1915
358.2195
364.6769
NL:
7.24E7
SUSSPE047-PG-HASP#35-40
RT: 0.96-1.10 AV: 6 T: FTMS
+ p APCI corona Full ms
[100.00-800.00]
Theoretical Isotope Model: [M + H]+
339.1959
340.1981
341.1928
345.1964
NL:
1.59E4
C21 H30 Si2 H:
C21 H31 Si2
p (gss, s /p:40) Chrg 1
R: 100000 Res .Pwr . @FWHM
Theoretical Isotope Model: [M + NH4]+
356.2224
357.2246
358.2194
362.2229
NL:
1.58E4
C21 H30 Si2 NH4:
C21 H34 Si2 N1
p (gss, s /p:40) Chrg 1
R: 100000 Res .Pwr . @FWHM
Relative Abundance
m/z

1,4-bis((Z)-2-phenyl-1,2-bis(trimethylsilyl)vinyl)benzene (10)

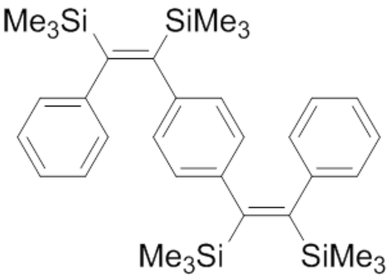

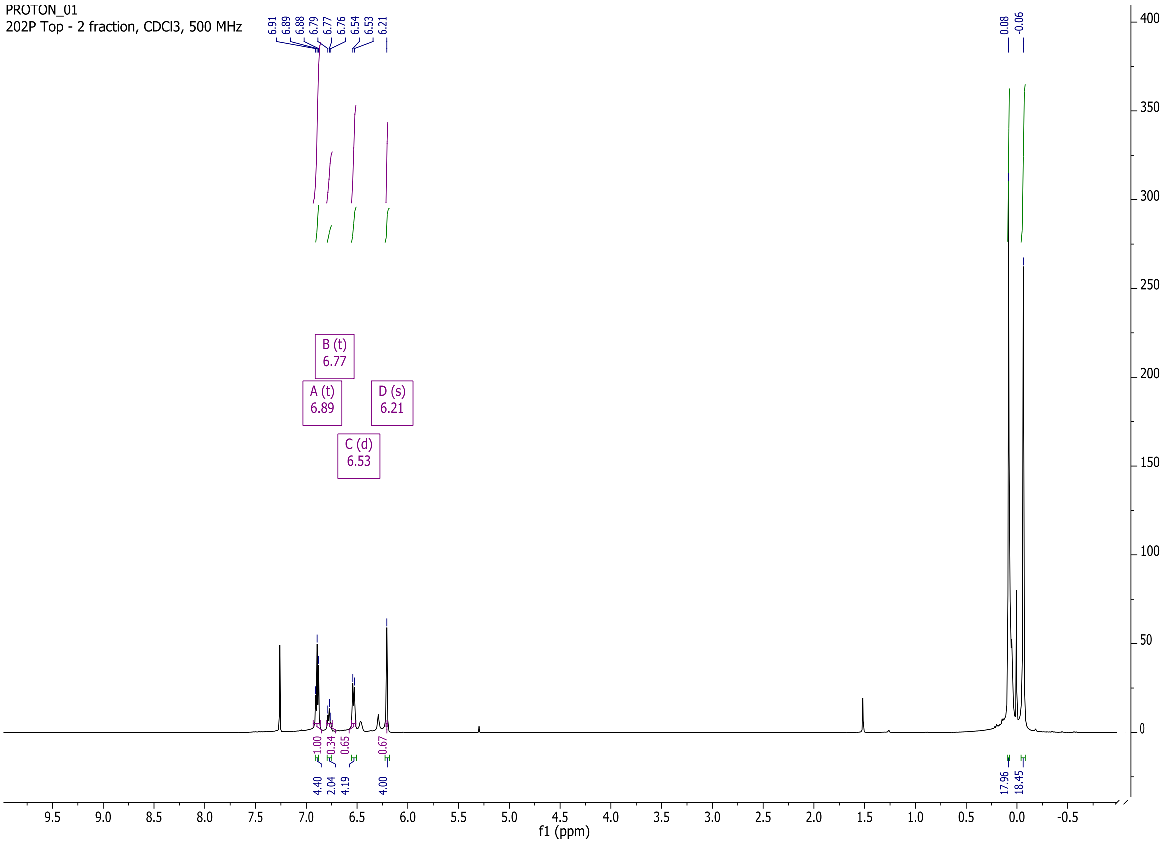

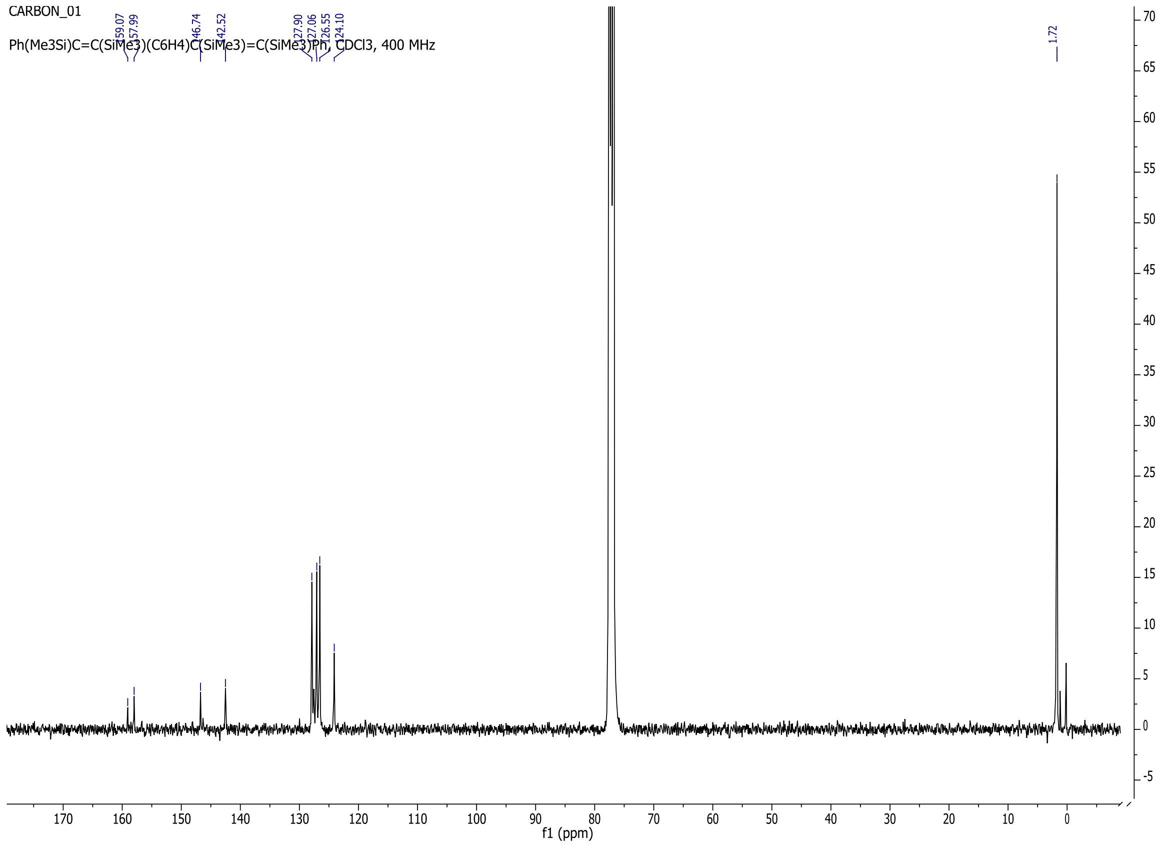
CARBON_01
Ph(Me3Si)C=C(SiMe3)(C6H4)C(SiMe3)=C(SiMe3)Ph, CDCl3, 400 MHz
159.07
157.99
146.74
142.52
127.90
127.06
126.55
124.10
1.72
f1 (ppm)

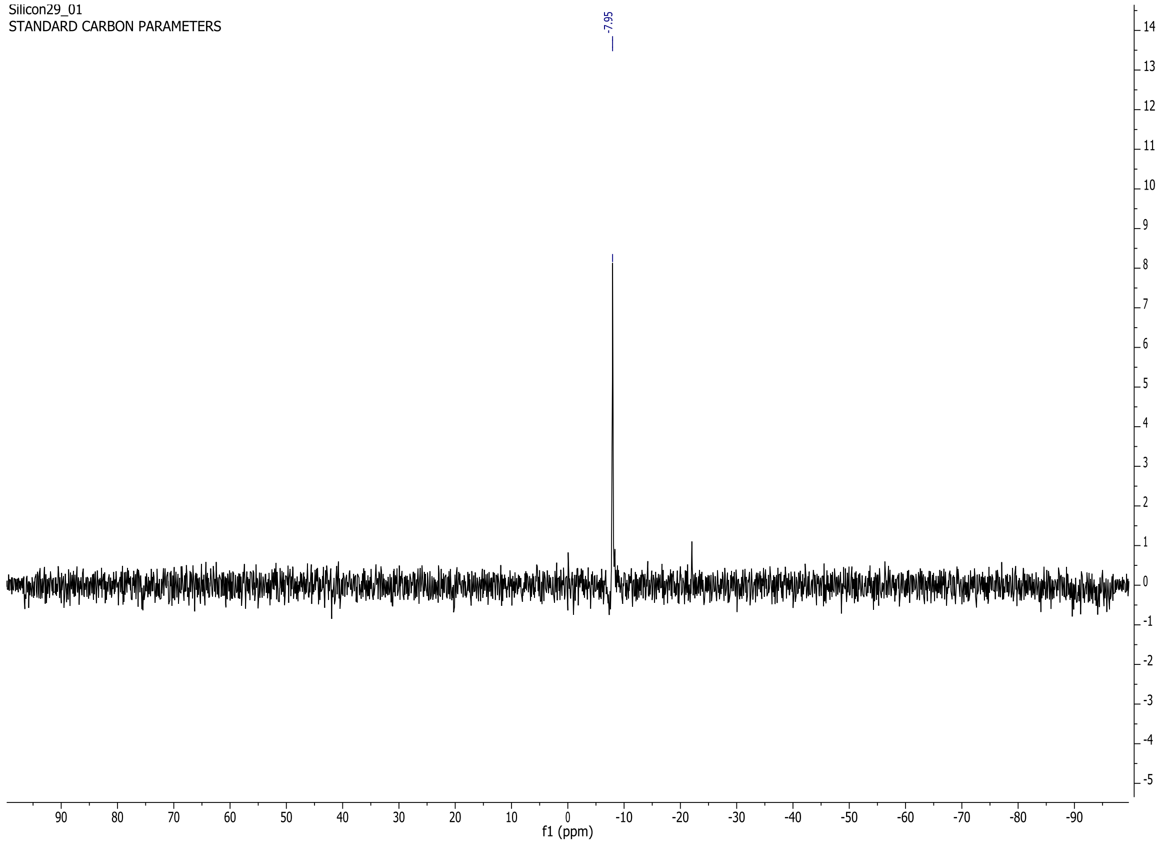
Silicon29_01
STANDARD CARBON PARAMETERS
-7.95
f1 (ppm)

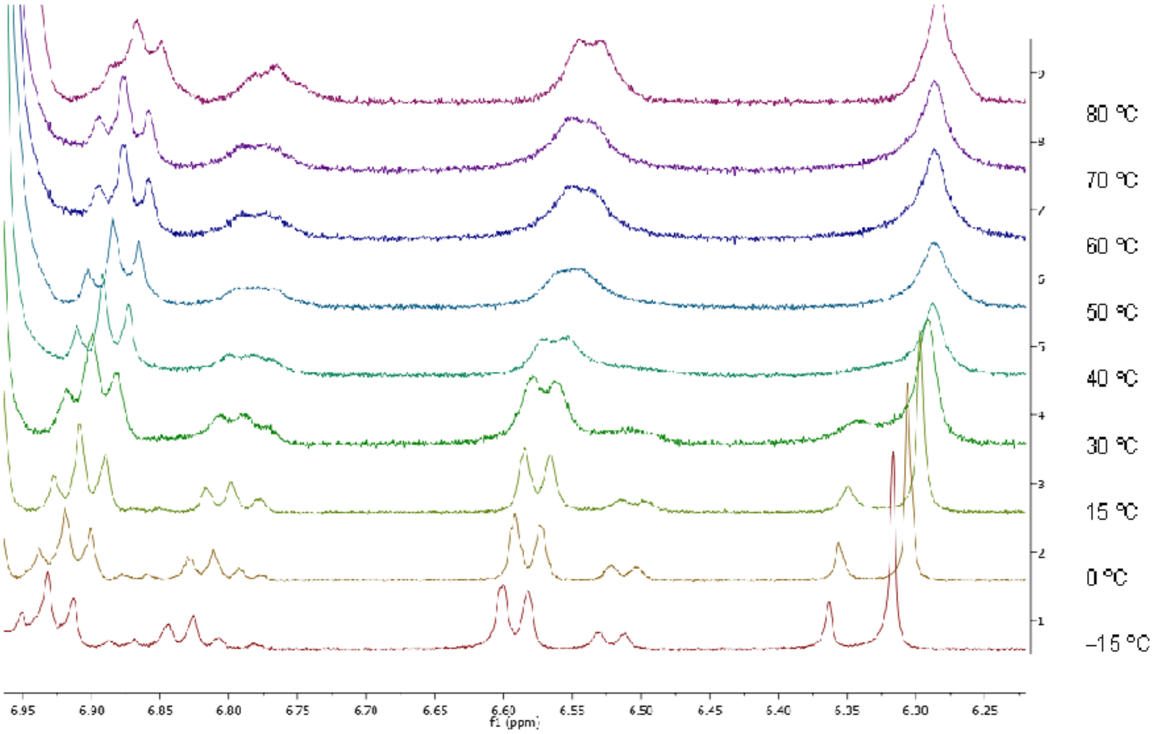
80 °C
70 °C
60 °C
50 °C
40 °C
30 °C
15 °C
0 °C
−15 °C
6.95
6.90
6.85
6.80
6.75
6.70
6.65
6.60
6.55
6.50
6.45
6.40
6.35
6.30
6.25
f1 (ppm)

cis-(Ph)(Me_3Si)C=C($SiMe_3$)(naphthalene) (11)

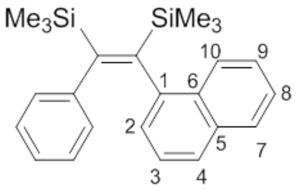

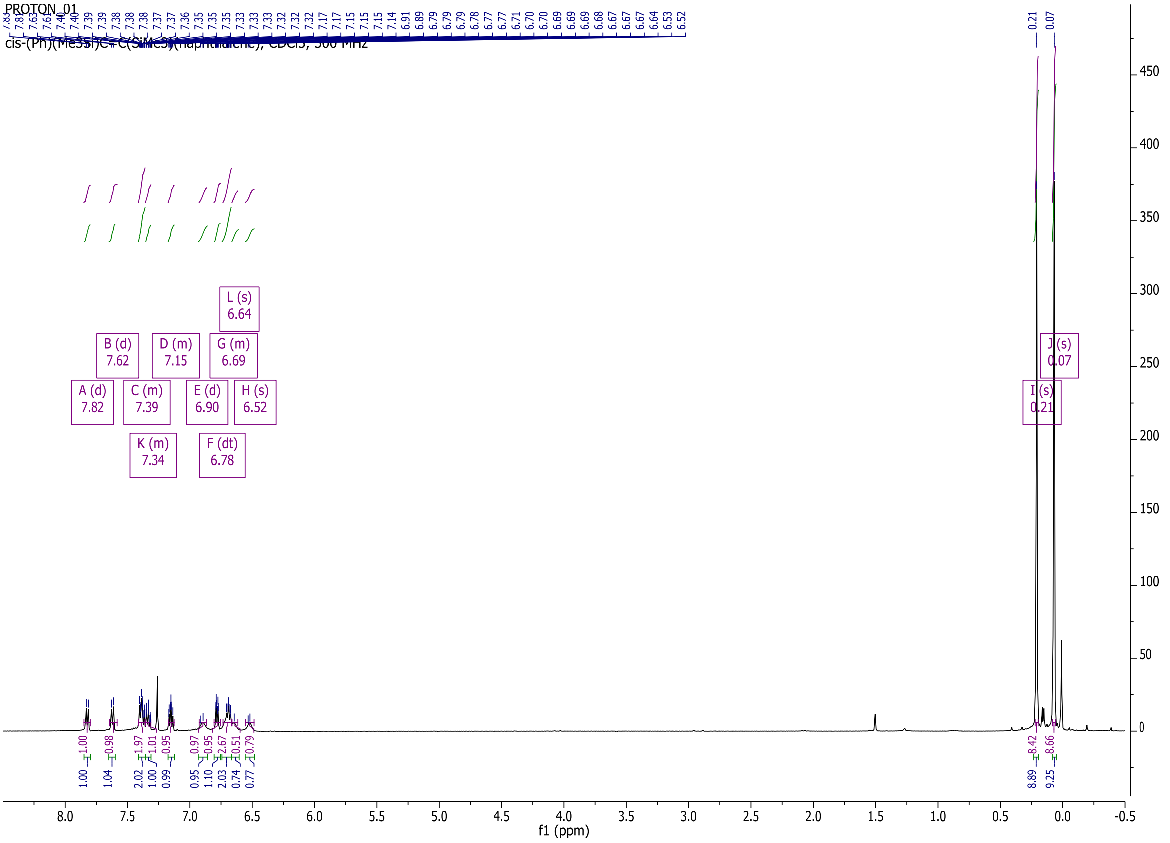

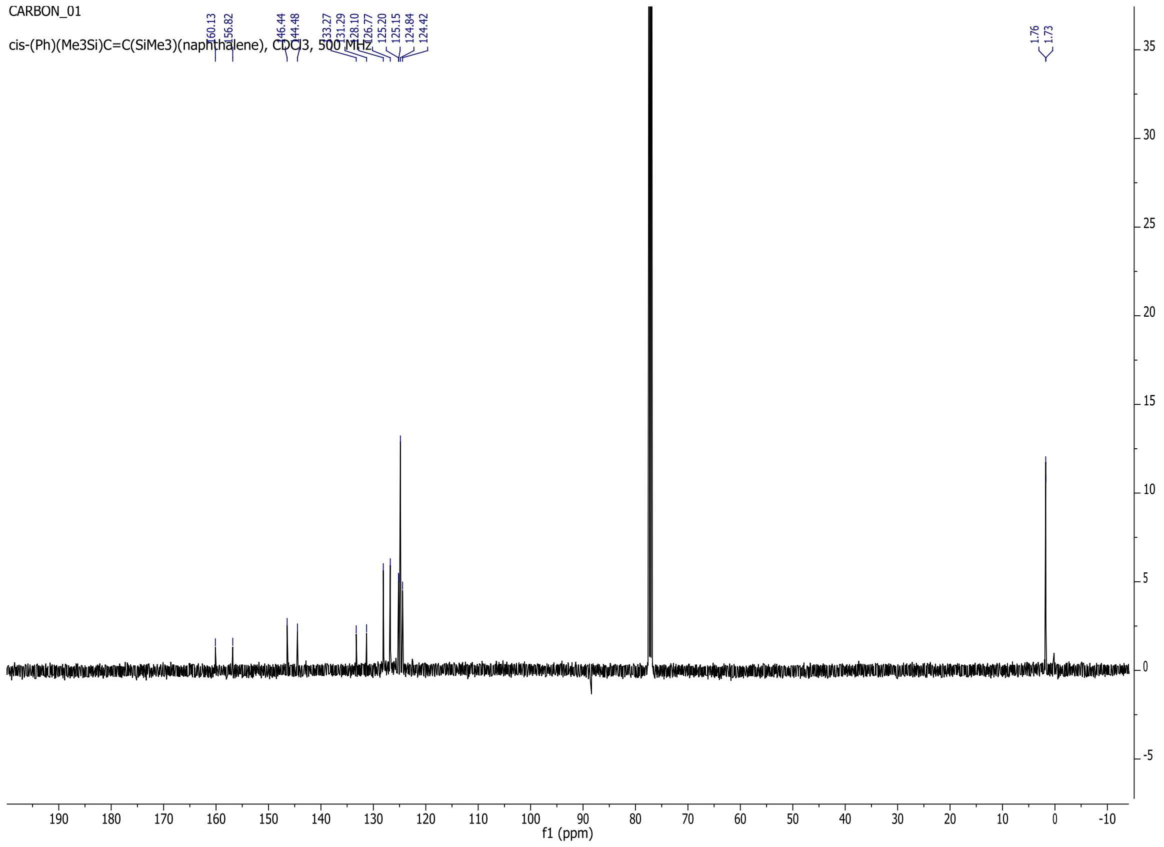
CARBON_01
cis-(Ph)(Me3Si)C=C(SiMe3)(naphthalene), CDCl3, 500 MHZ
160.13
156.82
146.44
144.48
133.27
131.29
128.10
126.77
125.20
125.15
124.84
124.42
1.76
1.73
f1 (ppm)

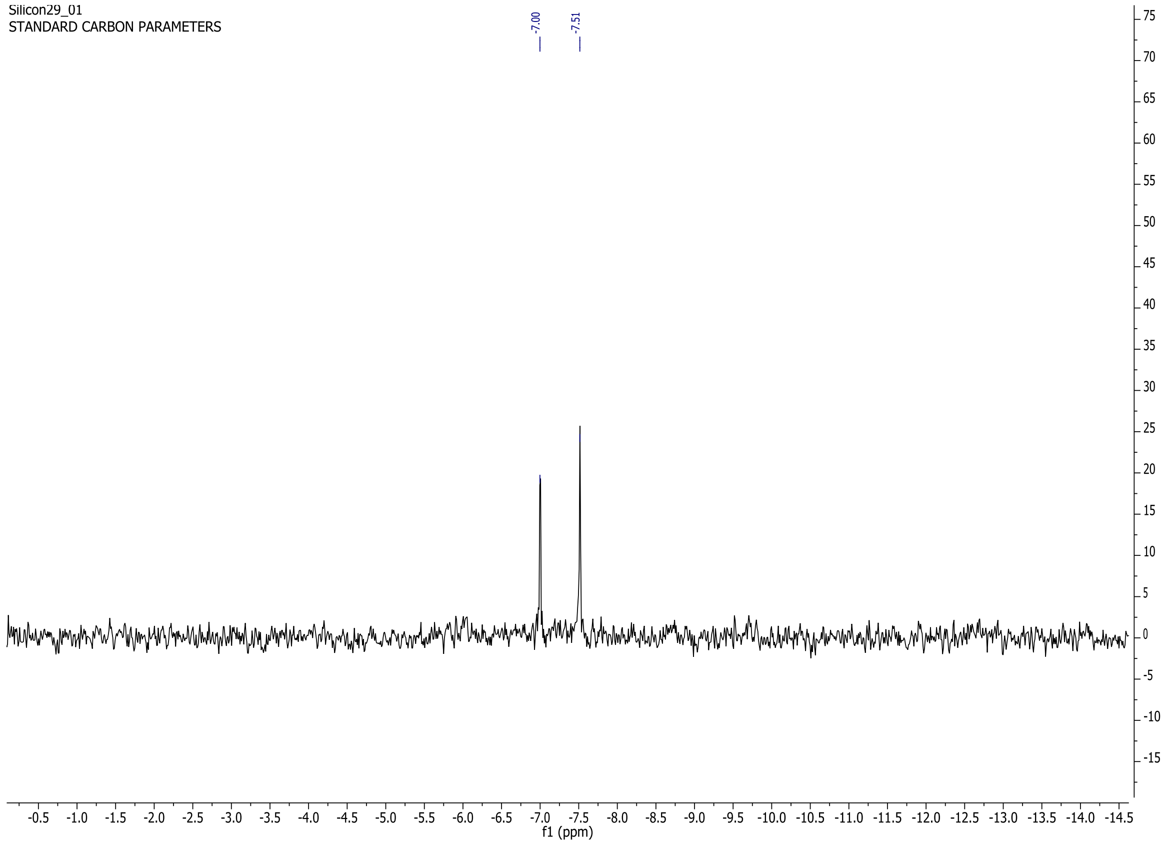
Silicon29_01
STANDARD CARBON PARAMETERS
-7.00
-7.51
f1 (ppm)

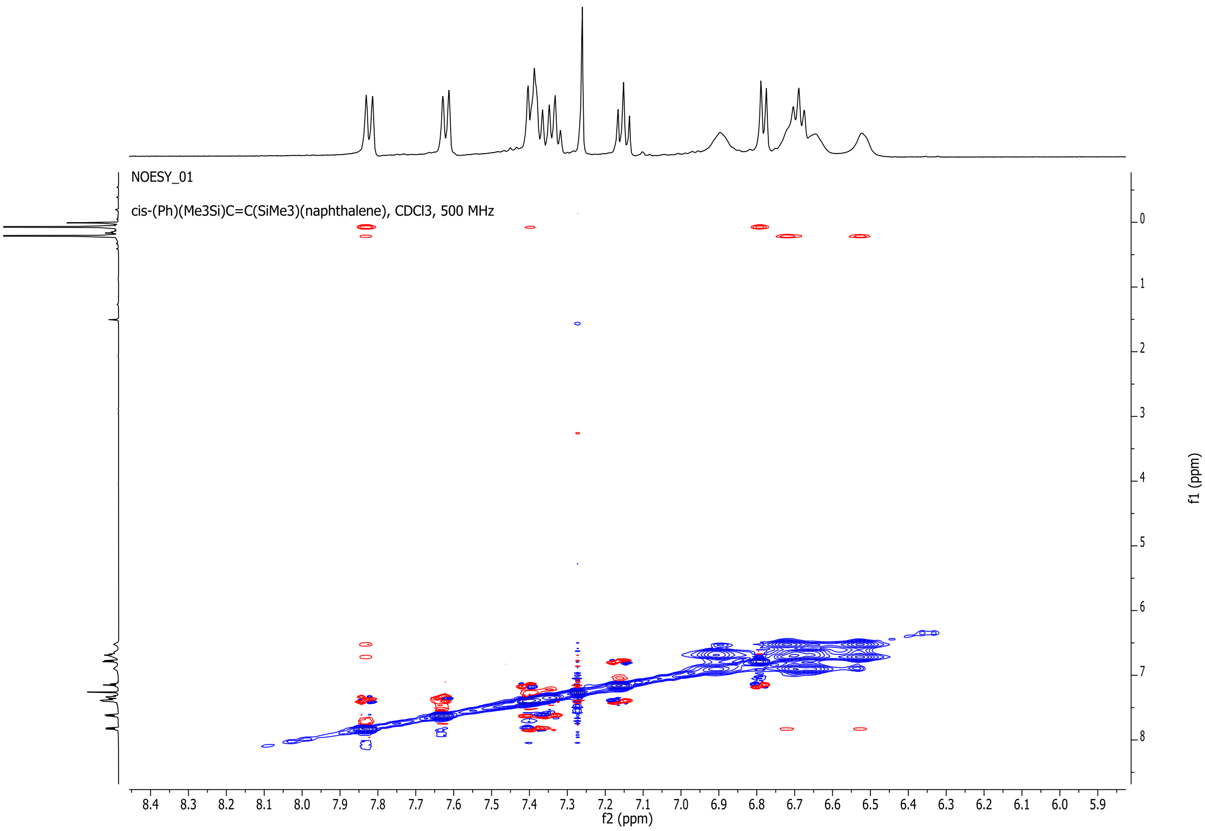

NOESY_01
cis-(Ph)(Me3Si)C=C(SiMe3)(naphthalene), CDCl3, 500 MHz
f2 (ppm)
f1 (ppm)

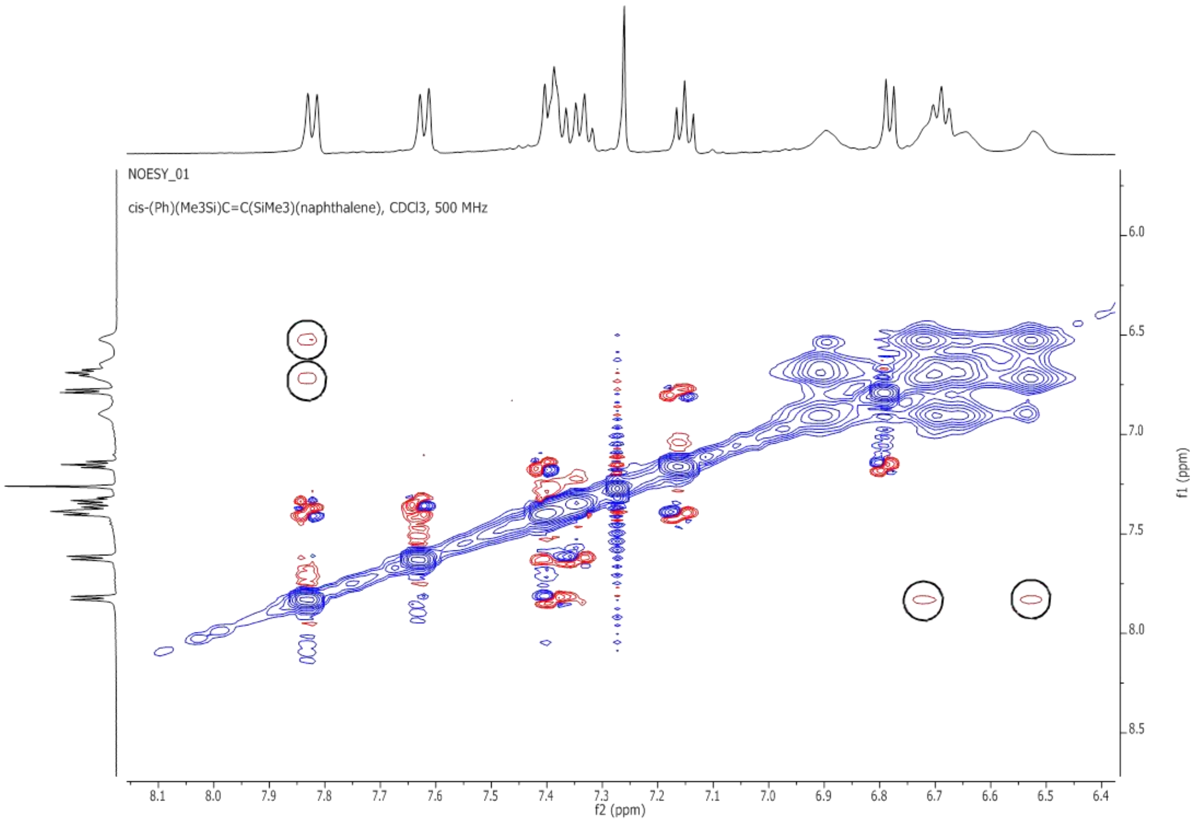

NOESY_01
cis-(Ph)(Me3Si)C=C(SiMe3)(naphthalene), CDCl3, 500 MHz
f2 (ppm)
f1 (ppm)

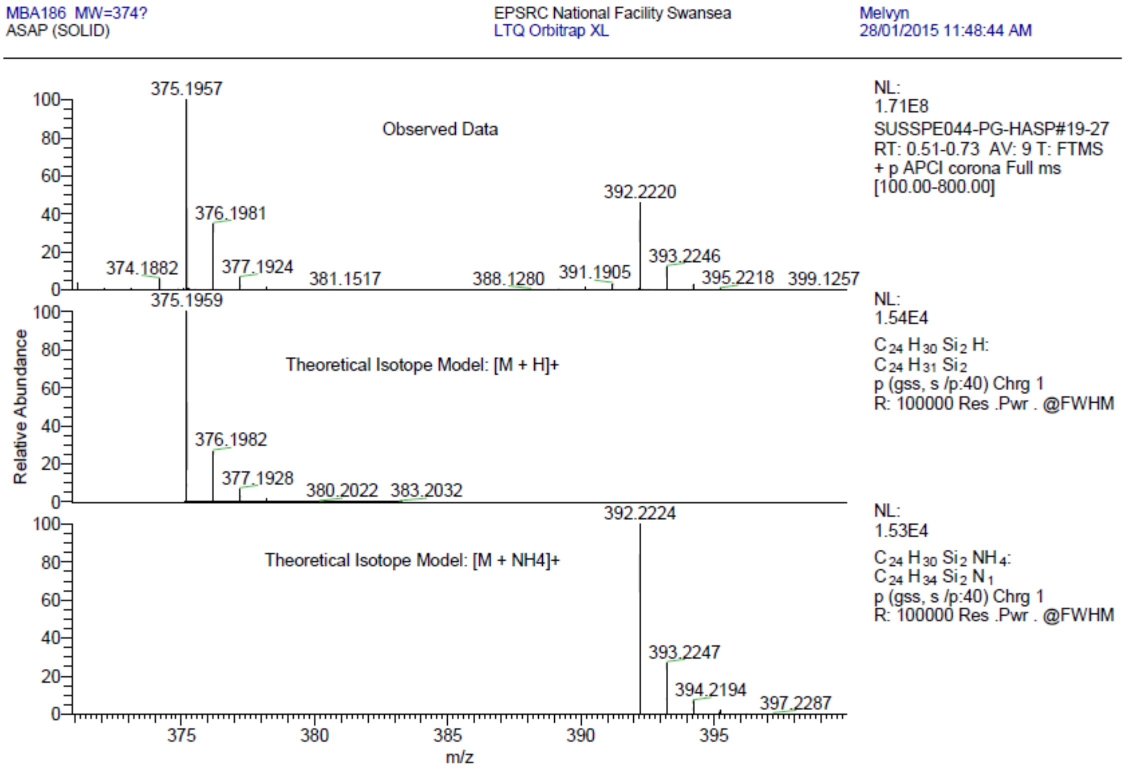

MBA186 MW=374?
ASAP (SOLID)
EPSRC National Facility Swansea
LTQ Orbitrap XL
Melvyn
28/01/2015 11:48:44 AM
375.1957
Observed Data
376.1981
374.1882
377.1924
381.1517
388.1280
391.1905
392.2220
393.2246
395.2218
399.1257
NL:
1.71E8
SUSSPE044-PG-HASP#19-27
RT: 0.51-0.73 AV: 9 T: FTMS
+ p APCI corona Full ms
[100.00-800.00]
375.1959
Theoretical Isotope Model: [M + H]+
376.1982
377.1928
380.2022
383.2032
NL:
1.54E4
C24 H30 Si2 H:
C24 H31 Si2
p (gss, s /p:40) Chrg 1
R: 100000 Res .Pwr . @FWHM
392.2224
Theoretical Isotope Model: [M + NH4]+
393.2247
394.2194
397.2287
NL:
1.53E4
C24 H30 Si2 NH4:
C24 H34 Si2 N1
p (gss, s /p:40) Chrg 1
R: 100000 Res .Pwr . @FWHM
Relative Abundance
m/z

1-phenyl-1,2,2-tris(trimethylsilyl)ethylene (12)

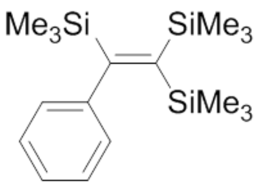

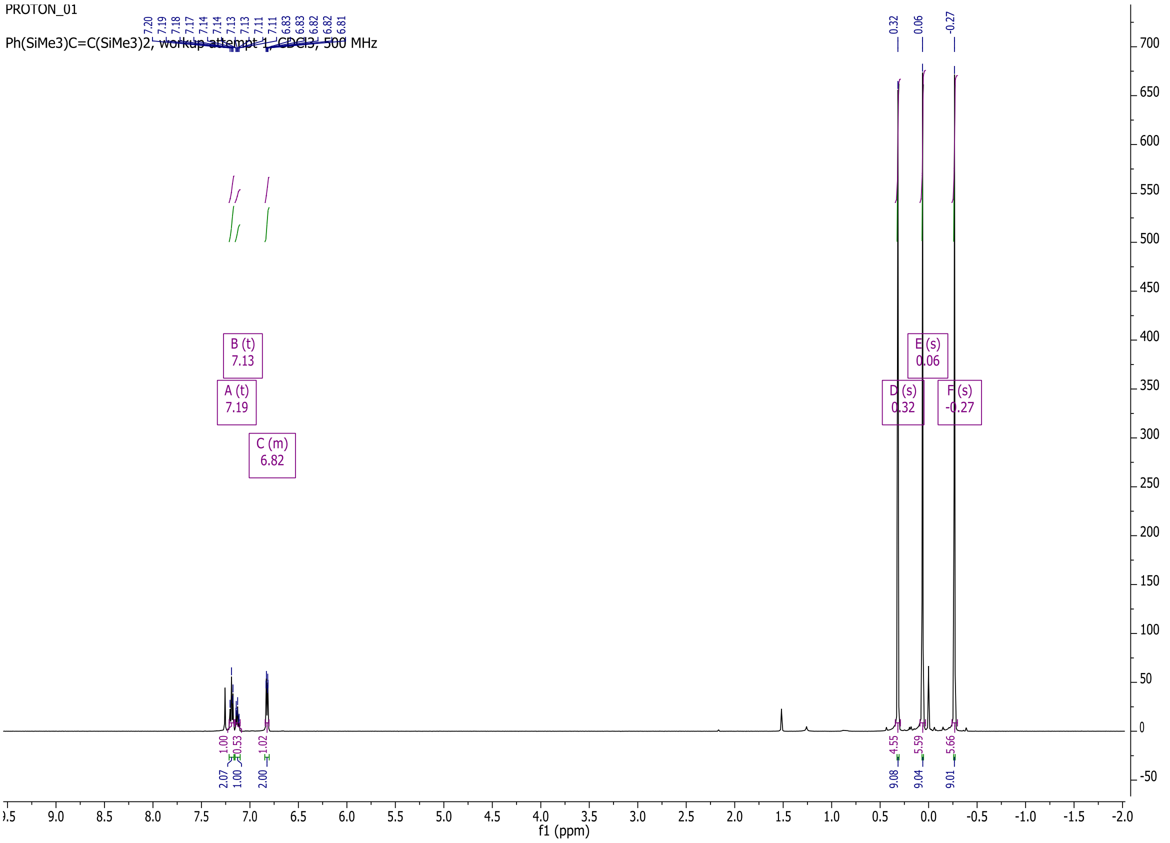

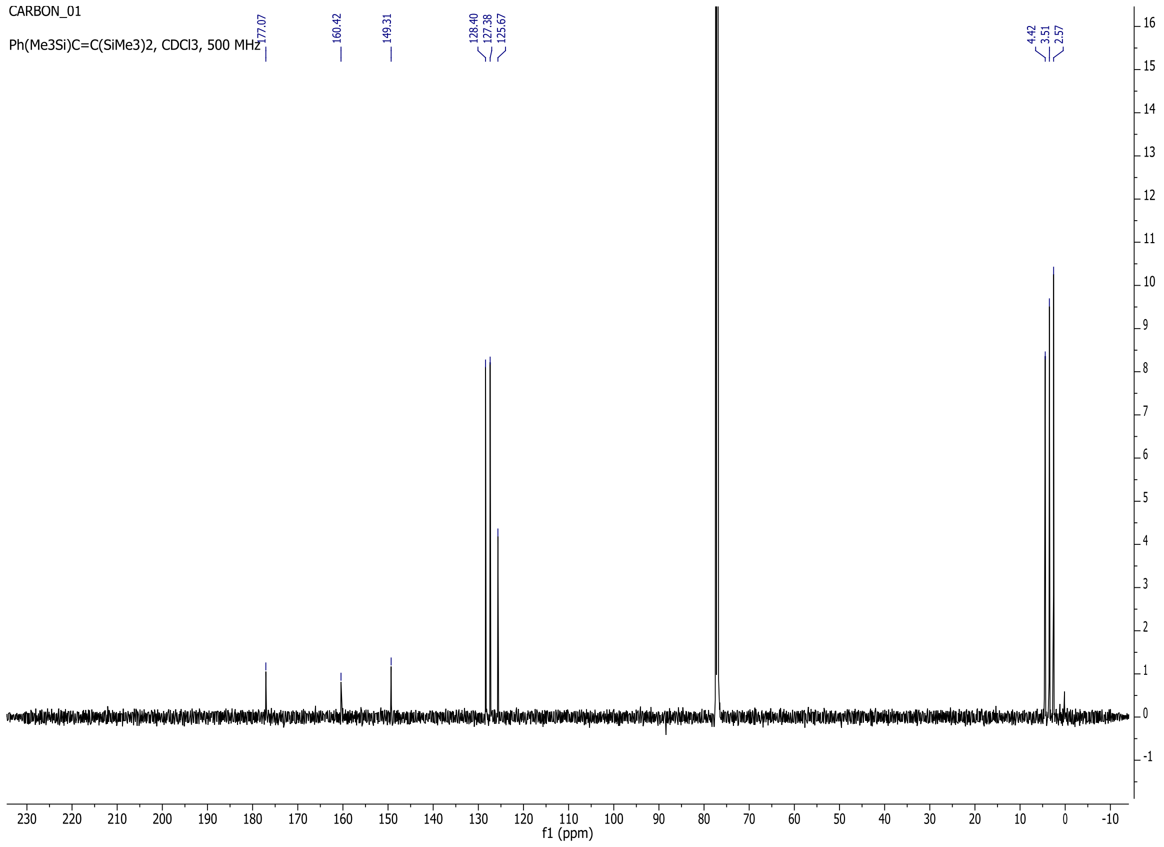

CARBON_01
Ph(Me3Si)C=C(SiMe3)2, CDCl3, 500 MHz
177.07
160.42
149.31
128.40
127.38
125.67
4.42
3.51
2.57
f1 (ppm)

cis-Ph(Me_3Si)C=C($SiMe_3$)H (13)

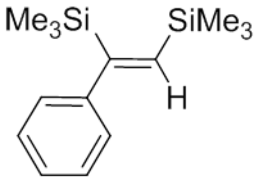

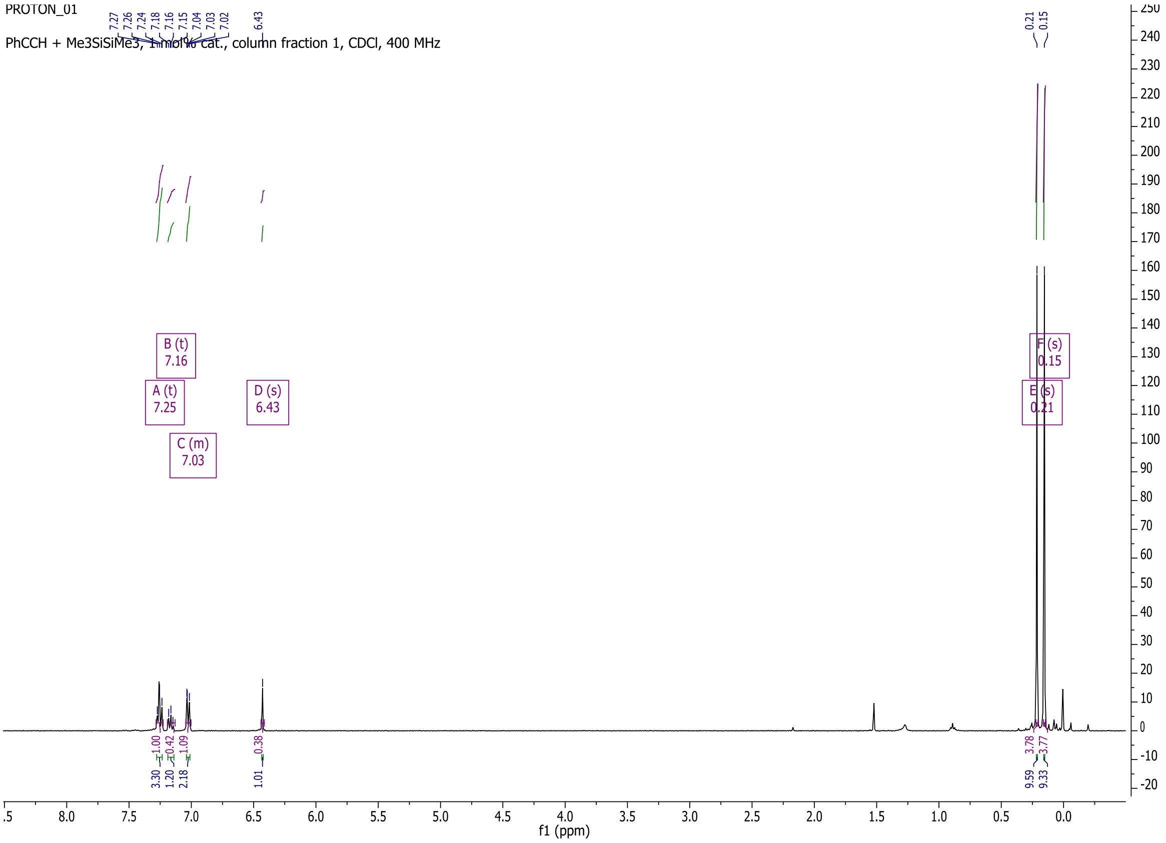

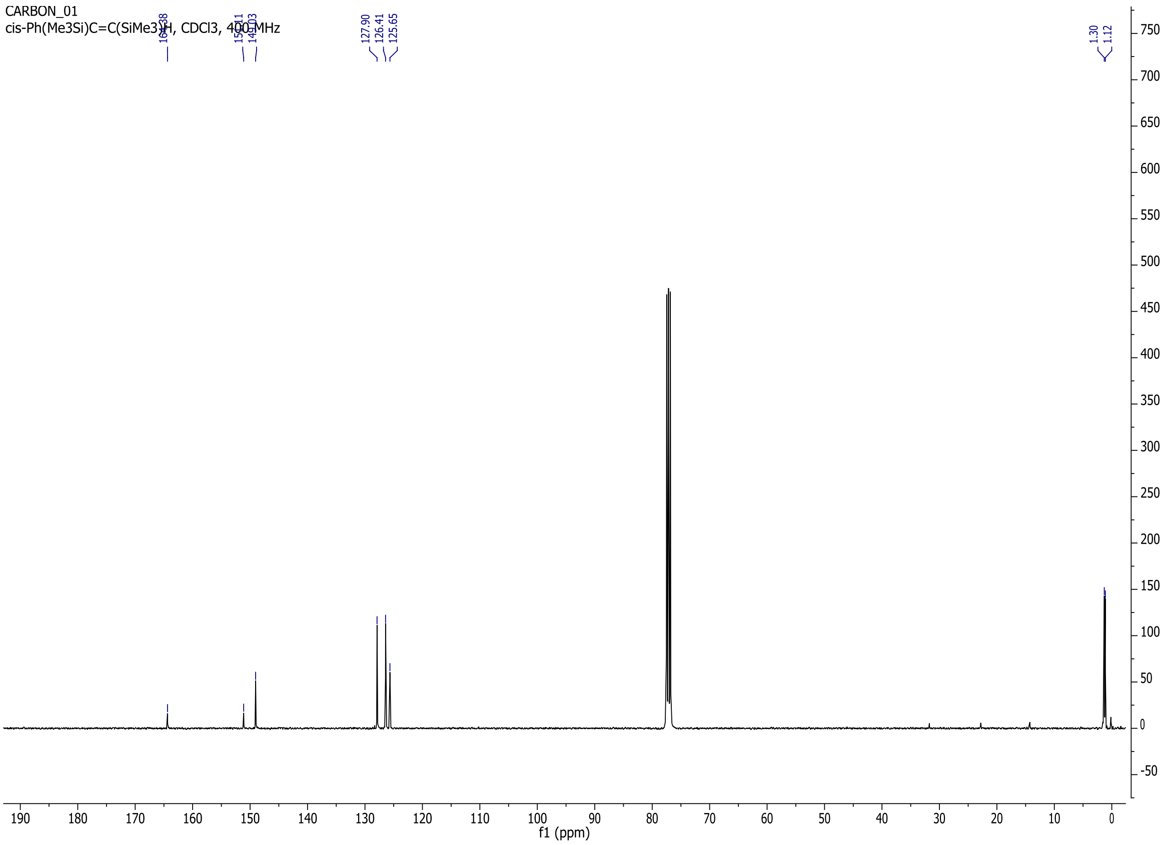
CARBON_01
cis-Ph(Me3Si)C=C(SiMe3)H, CDCl3, 400 MHz
127.90
126.41
125.65
1.30
1.12
f1 (ppm)

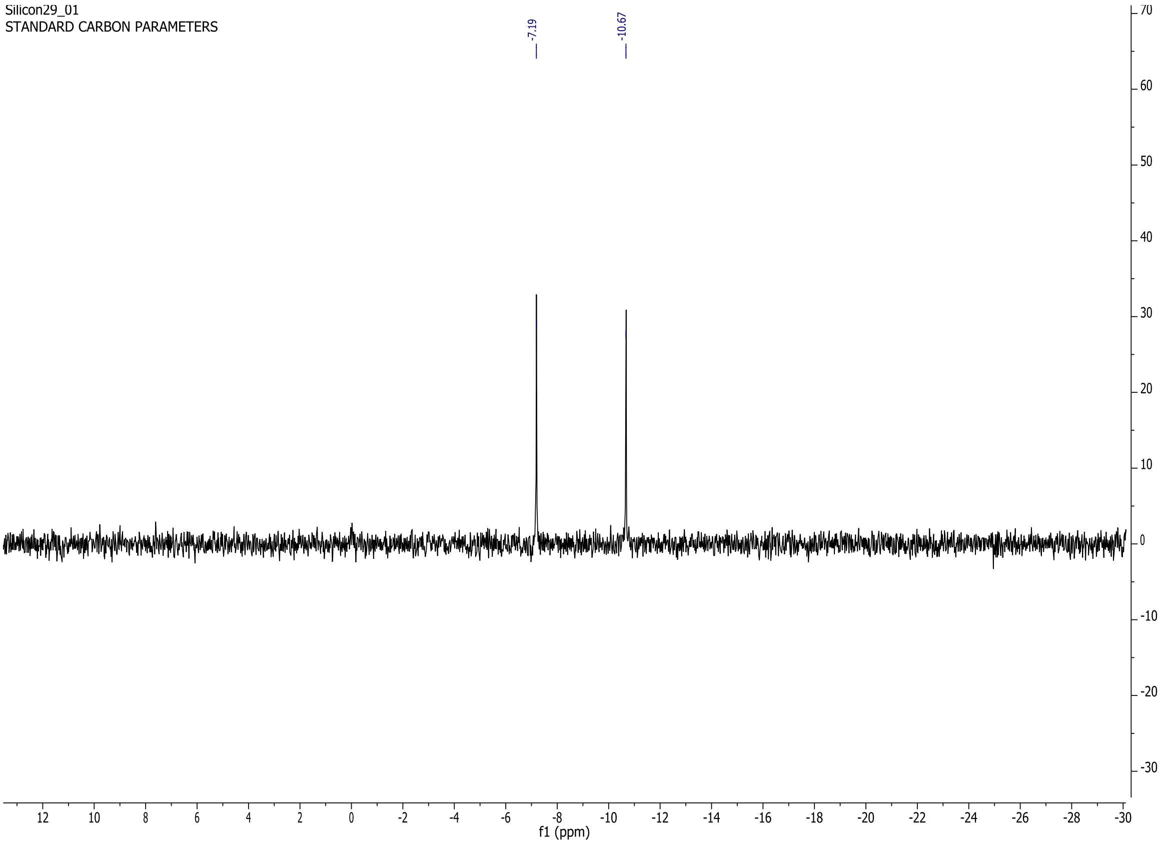
Silicon29_01
STANDARD CARBON PARAMETERS
-7.19
-10.67
f1 (ppm)

X-ray Crystallography Data:

Single crystal X-ray diffraction data for *cis*-Pd(ITMe)$_2$(SiMe$_3$)$_2$, Pd(ITMe)$_2$(PhCCPh) and *cis*-(Ph)(Me$_3$Si)C=C(SiMe$_3$)(Ph) collected at the University of Sussex on an Agilent Technologies Xcalibur Gemini Ultra diffractometer ($\lambda_{CuK\alpha}$ = 1.54184) equipped with a Eos CCD area detector. The data were collected at 173 K using an Oxford Cryosystems Cobra low temperature device. Data were processed using CrysAlisPro, and the unit cell parameters were refined against all data. Semi-empirical absorption corrections were carried out using the MULTI-SCAN program.[3] The structures were solved by charge-flipping using SUPERFLIP,[4] and refined F_0 by full matrix by full matrix least squares refinement using SHELXL-2013,[5] within OLEX2.[6] All non-hydrogen atoms were refined with anisotropic displacement parameters. Hydrogen atoms were added at calculated positions and refined using a riding models with isotropic displacement parameters based on the equivalent isotropic displacement parameter (Ueq) of the parent atom. The CIF files for *cis*-Pd(ITMe)$_2$(SiMe$_3$)$_2$, Pd(ITMe)$_2$(PhCCPh) and *cis*-(Ph)(Me$_3$Si)C=C(SiMe$_3$)(Ph) have been deposited with the CCDC and been given the deposition numbers 1029150, 1029151 and 1045559 respectively.

*Crystal structure data for **4***

Table S1. Crystal data and structure refinement data for 4

Empirical formula	$C_{20}H_{42}N_4PdSi_2$
Formula weight	501.15
Temperature/K	173
Crystal system	triclinic
Space group	P-1

a/Å	9.7136(4)
b/Å	10.2767(6)
c/Å	15.7695(9)
α/°	91.163(5)
β/°	100.298(4)
γ/°	108.564(4)
Volume/Å^3	1463.19(13)
Z	2
ρ_{calc}g/cm^3	1.137
μ/mm^{-1}	5.967
F(000)	528.0
Crystal size/mm^3	0.34 × 0.28 × 0.2
Radiation	CuKα (λ = 1.54184)
2Θ range for data collection/°	5.716 to 142.226
Completeness to θ = 71.113	95.7 %
Index ranges	-11 ≤ h ≤ 6, -11 ≤ k ≤ 12, -17 ≤ l ≤ 19
Reflections collected	7897
Independent reflections	5423 [R_{int} = 0.0303, R_{sigma} = 0.0509]
Data/restraints/parameters	5423/0/258
Goodness-of-fit on F^2	0.996
Final R indexes [I>=2σ (I)]	R_1 = 0.0271, wR_2 = 0.0645
Final R indexes [all data]	R_1 = 0.0297, wR_2 = 0.0659

Largest diff. peak/hole / e Å^{-3} 0.39/-0.55

Table S2. Fractional Atomic Coordinates (×10^4) and Equivalent Isotropic Displacement Parameters (Å^2×10^3) for 4. U_{eq} is defined as 1/3 of of the trace of the orthogonalised U_{IJ} tensor.

**Atom**	***x***	***y***	***z***	**U(eq)**
Pd1	1907.4(2)	8794.6(2)	7099.2(2)	22.09(6)
Si2	-272.3(6)	6956.0(6)	6569.9(4)	28.84(12)
Si1	1011.4(7)	8971.8(7)	8374.6(4)	31.00(13)
C7	2705(2)	8438(2)	5991.8(13)	25.0(4)
N2	3385.2(19)	7501.7(19)	5855.8(12)	26.8(3)
N4	3737(2)	11933(2)	7484.9(12)	29.2(4)
N1	2679.2(19)	9071.7(19)	5246.7(11)	26.4(3)
N3	5062(2)	10729.0(19)	8006.0(11)	26.7(3)
C14	3700(2)	10616(2)	7567.2(13)	26.4(4)
C18	5121(3)	12866(2)	7872.1(14)	32.9(5)
C17	5953(3)	12100(2)	8199.7(14)	31.9(4)
C13	4481(3)	6611(3)	4711.1(17)	40.9(5)
C11	3758(2)	7547(2)	5036.2(14)	30.3(4)
C3	2572(3)	9857(3)	9326.2(16)	47.0(6)
C9	3647(3)	6546(2)	6482.5(16)	36.1(5)
C12	3412(3)	9075(3)	3786.0(16)	43.5(6)
C5	-638(3)	6653(3)	5336.5(16)	43.1(6)

C8	2023(3)	10151(3)	5087.3(16)	37.6(5)
C19	7522(3)	12526(3)	8675.9(19)	45.7(6)
C15	5515(3)	9544(3)	8239.5(15)	35.6(5)
C20	5470(3)	14382(3)	7864.3(18)	45.2(6)
C4	-2107(2)	7159(3)	6713.3(16)	37.8(5)
C16	2489(3)	12343(3)	7079.8(18)	40.7(5)
C10	3311(2)	8542(2)	4654.9(14)	30.9(4)
C6	-360(3)	5178(3)	6933(2)	50.7(7)
C2	-213(4)	10104(4)	8331(2)	52.9(7)
C1	1(4)	7384(3)	8895.9(17)	52.9(7)

Table S3. Anisotropic Displacement Parameters (Å^2×10^3) for 4. The Anisotropic displacement factor exponent takes the form: -2π^2[h^{2}a*^{2}U$_{11}$+2hka*b*U$_{12}$+…].

**Atom**	**U$_{11}$**	**U$_{22}$**	**U$_{33}$**	**U$_{23}$**	**U$_{13}$**	**U$_{12}$**
Pd1	19.22(8)	25.87(9)	19.62(8)	2.26(5)	4.45(5)	4.87(6)
Si2	22.5(3)	30.0(3)	28.0(3)	2.2(2)	3.2(2)	1.1(2)
Si1	27.5(3)	40.7(3)	23.0(3)	1.7(2)	7.8(2)	7.3(2)
C7	20.5(9)	27.8(9)	24.0(9)	1.3(7)	4.6(7)	4.0(8)
N2	22.8(8)	29.3(8)	27.5(8)	0.2(7)	4.9(6)	7.6(7)
N4	30.3(9)	30.3(9)	25.6(8)	2.1(7)	6.6(7)	7.3(7)
N1	24.1(8)	30.3(9)	24.1(8)	4.3(7)	8.0(6)	5.8(7)
N3	23.2(8)	30.7(9)	22.4(8)	1.9(6)	4.9(6)	3.4(7)

C14	22.9(9)	30.5(10)	21.3(9)	2.0(7)	8.0(7)	0.6(8)
C18	34.8(11)	32.5(11)	27.6(10)	1.2(8)	10.8(8)	3.1(9)
C17	29.7(11)	35.3(11)	25.6(10)	0.5(8)	8.1(8)	2.3(9)
C13	35.8(12)	44.5(13)	45.1(13)	-10.4(10)	12.5(10)	15.2(11)
C11	23.5(10)	35.2(11)	29.9(10)	-5.9(8)	6.8(8)	5.9(8)
C3	36.7(13)	69.2(18)	25.5(11)	-7.2(11)	6.5(9)	4.6(12)
C9	36.8(12)	34.4(11)	39.0(12)	4.3(9)	4.7(9)	15.7(10)
C12	44.7(14)	56.7(15)	27.8(11)	4.2(10)	13.6(10)	11.0(12)
C5	35.3(12)	49.0(14)	32.7(11)	-6.2(10)	5.7(9)	-2.2(11)
C8	42.8(13)	38.5(12)	36.3(11)	9.9(9)	11(1)	17.5(10)
C19	34.5(13)	42.6(13)	45.5(14)	-0.6(11)	-2(1)	-1.8(10)
C15	30.3(11)	38.5(12)	34.9(11)	4.3(9)	1.5(9)	9.6(9)
C20	54.9(16)	30.7(12)	42.8(13)	3.4(10)	13.6(11)	2.0(11)
C4	23.6(10)	47.1(13)	37.9(12)	6(1)	5.1(9)	5.2(9)
C16	39.3(13)	39.6(12)	44.4(13)	5.2(10)	5(1)	16.2(10)
C10	26.4(10)	36.7(11)	26.7(10)	-1.7(8)	9.0(8)	4.6(8)
C6	47.5(15)	32.6(12)	64.8(18)	10.3(12)	7.2(13)	5.0(11)
C2	51.2(16)	72.1(19)	48.7(15)	4.1(13)	20.7(12)	32.8(15)
C1	53.8(16)	59.8(17)	32.7(12)	11.4(12)	13.7(11)	-1.9(13)

Table S4. Bond Lengths for 4

Atom	Atom	Length/Å	Atom	Atom	Length/Å
Pd1	Si2	2.3466(6)	N4	C14	1.352(3)
Pd1	Si1	2.3554(6)	N4	C18	1.400(3)
Pd1	C7	2.103(2)	N4	C16	1.454(3)
Pd1	C14	2.119(2)	N1	C8	1.451(3)
Si2	C5	1.914(3)	N1	C10	1.393(3)
Si2	C4	1.911(3)	N3	C14	1.347(3)
Si2	C6	1.906(3)	N3	C17	1.393(3)
Si1	C3	1.905(3)	N3	C15	1.454(3)
Si1	C2	1.905(3)	C18	C17	1.346(4)
Si1	C1	1.904(3)	C18	C20	1.486(3)
C7	N2	1.362(3)	C17	C19	1.493(3)
C7	N1	1.355(3)	C13	C11	1.490(3)
N2	C11	1.401(3)	C11	C10	1.345(4)
N2	C9	1.454(3)	C12	C10	1.494(3)

Table S5. Bond angles for 4.

Atom	Atom	Atom	Angle/°	Atom	Atom	Atom	Angle/°
Si2	Pd1	Si1	88.64(2)	C14	N4	C18	111.38(19)
C7	Pd1	Si2	88.72(6)	C14	N4	C16	124.7(2)
C7	Pd1	Si1	174.27(6)	C18	N4	C16	123.8(2)

C7	Pd1	C14	94.78(8)	C7	N1	C8	122.97(19)
C14	Pd1	Si2	172.63(6)	C7	N1	C10	112.40(19)
C14	Pd1	Si1	88.42(6)	C10	N1	C8	124.61(19)
C5	Si2	Pd1	111.85(8)	C14	N3	C17	111.76(19)
C4	Si2	Pd1	117.80(8)	C14	N3	C15	123.04(19)
C4	Si2	C5	99.77(12)	C17	N3	C15	125.20(19)
C6	Si2	Pd1	117.52(10)	N4	C14	Pd1	127.66(16)
C6	Si2	C5	102.54(14)	N3	C14	Pd1	128.09(16)
C6	Si2	C4	104.92(13)	N3	C14	N4	104.25(18)
C3	Si1	Pd1	111.91(8)	N4	C18	C20	122.5(2)
C2	Si1	Pd1	114.62(10)	C17	C18	N4	106.1(2)
C2	Si1	C3	102.09(15)	C17	C18	C20	131.3(2)
C1	Si1	Pd1	121.76(10)	N3	C17	C19	123.2(2)
C1	Si1	C3	99.40(13)	C18	C17	N3	106.5(2)
C1	Si1	C2	104.39(16)	C18	C17	C19	130.3(2)
N2	C7	Pd1	127.75(15)	N2	C11	C13	123.2(2)
N1	C7	Pd1	129.15(16)	C10	C11	N2	106.09(19)
N1	C7	N2	103.10(18)	C10	C11	C13	130.7(2)
C7	N2	C11	111.97(18)	N1	C10	C12	122.4(2)
C7	N2	C9	123.50(19)	C11	C10	N1	106.44(19)
C11	N2	C9	124.51(19)	C11	C10	C12	131.2(2)

Table S6. Torsion angles for 4

A	B	C	D	Angle/°	A	B	C	D	Angle/°
Pd1	C7	N2	C11	179.56(14)	C18	N4	C14	Pd1	-179.46(15)
Pd1	C7	N2	C9	1.3(3)	C18	N4	C14	N3	0.0(2)
Pd1	C7	N1	C8	-1.3(3)	C17	N3	C14	Pd1	179.43(14)
Pd1	C7	N1	C10	-179.65(15)	C17	N3	C14	N4	0.0(2)
C7	N2	C11	C13	-178.5(2)	C13	C11	C10	N1	178.8(2)
C7	N2	C11	C10	0.5(2)	C13	C11	C10	C12	-1.5(4)
C7	N1	C10	C11	-0.3(2)	C9	N2	C11	C13	-0.3(3)
C7	N1	C10	C12	-180.0(2)	C9	N2	C11	C10	178.8(2)
N2	C7	N1	C8	178.9(2)	C8	N1	C10	C11	-178.6(2)
N2	C7	N1	C10	0.6(2)	C8	N1	C10	C12	1.7(3)
N2	C11	C10	N1	-0.1(2)	C15	N3	C14	Pd1	-0.5(3)
N2	C11	C10	C12	179.5(2)	C15	N3	C14	N4	-179.96(19)
N4	C18	C17	N3	0.0(2)	C15	N3	C17	C18	180.0(2)
N4	C18	C17	C19	179.4(2)	C15	N3	C17	C19	0.5(4)
N1	C7	N2	C11	-0.7(2)	C20	C18	C17	N3	-179.0(2)
N1	C7	N2	C9	-178.94(19)	C20	C18	C17	C19	0.5(4)
C14	N4	C18	C17	0.0(2)	C16	N4	C14	Pd1	3.0(3)
C14	N4	C18	C20	179.1(2)	C16	N4	C14	N3	-177.6(2)
C14	N3	C17	C18	0.0(3)	C16	N4	C18	C17	177.6(2)
C14	N3	C17	C19	-179.5(2)	C16	N4	C18	C20	-3.3(3)

Table S7. Hydrogen Atom Coordinates (Å×10⁴) and Isotropic Displacement Parameters (Å²×10³) for 4

**Atom**	***x***	***y***	***z***	**U(eq)**
H13A	5432	6737	5098	61
H13B	4648	6827	4128	61
H13C	3839	5653	4694	61
H3A	3241	9314	9442	70
H3B	2154	9934	9839	70
H3C	3123	10779	9187	70
H9A	4714	6776	6698	54
H9B	3244	5605	6206	54
H9C	3159	6614	6967	54
H12A	2413	8912	3449	65
H12B	3932	8597	3482	65
H12C	3958	10065	3856	65
H5A	-709	7493	5076	65
H5B	-1569	5897	5139	65
H5C	177	6413	5163	65
H8A	1504	10236	5553	56
H8B	1320	9921	4534	56
H8C	2803	11027	5067	56
H19A	7560	12140	9239	69

H19B	7925	13534	8761	69
H19C	8111	12185	8339	69
H15A	4655	8703	8102	53
H15B	5931	9644	8860	53
H15C	6267	9487	7913	53
H20A	5489	14644	7271	68
H20B	6440	14846	8233	68
H20C	4711	14655	8083	68
H4A	-2141	7206	7330	57
H4B	-2929	6368	6411	57
H4C	-2193	8008	6472	57
H16A	2717	12813	6562	61
H16B	2301	12968	7488	61
H16C	1609	11524	6916	61
H6A	355	4864	6694	76
H6B	-1360	4529	6726	76
H6C	-121	5228	7566	76
H2A	389	11068	8316	79
H2B	-664	10008	8844	79
H2C	-993	9820	7810	79
H1A	-920	6860	8500	79
H1B	-230	7670	9436	79

H1C 632 6807 9020 79

Crystal structure data for ***6***

Table S8. Crystal data and structure refinement data for 6

Empirical formula	$C_{28}H_{34}N_4Pd$
Formula weight	532.99
Temperature/K	173.15
Crystal system	triclinic
Space group	P-1
a/Å	10.3873(6)
b/Å	11.4638(5)
c/Å	11.6798(5)
α/°	68.640(4)
β/°	87.052(4)
γ/°	88.047(4)
Volume/Å^3	1293.37(11)
Z	2
ρ_{calc}g/cm^3	1.369
μ/mm^{-1}	5.943
F(000)	552.0
Crystal size/mm^3	0.2 × 0.05 × 0.05
Radiation	CuKα (λ = 1.54184)

2Θ range for data collection/°	8.136 to 141.736
Completeness to θ = 70.868	96.6 %
Index ranges	$-12 \leq h \leq 12$, $-14 \leq k \leq 13$, $-14 \leq l \leq 9$
Reflections collected	8237
Independent reflections	4826 [R_{int} = 0.0319, R_{sigma} = 0.0470]
Data/restraints/parameters	4826/0/306
Goodness-of-fit on F^2	1.019
Final R indexes [I>=2σ (I)]	R_1 = 0.0281, wR_2 = 0.0683
Final R indexes [all data]	R_1 = 0.0323, wR_2 = 0.0702
Largest diff. peak/hole / e $Å^{-3}$	0.32/-0.66

Table S9. Fractional Atomic Coordinates (×10^4) and Equivalent Isotropic Displacement Parameters ($Å^2$×10^3) for 6. U_{eq} is defined as 1/3 of of the trace of the orthogonalised U_{IJ} tensor.

**Atom**	***x***	***y***	***z***	**U(eq)**
Pd1	145.0(2)	5333.4(2)	7377.7(2)	25.39(7)
N2	2597(2)	6023(2)	5663(2)	36.1(5)
N4	-1322(2)	7472(2)	7958(2)	31.0(5)
C5	846(2)	2351(2)	8040(2)	27.3(5)
C8	4918(3)	7741(4)	6685(4)	62.5(10)
N9	2774(2)	6633(2)	7162(2)	32.4(5)
N12	-1049(2)	7982(2)	6019(2)	33.7(5)
C13	2470(3)	6838(3)	8300(3)	46.6(7)

C16	4614(4)	6837(5)	4334(4)	74.8(13)
C17	-2163(4)	10098(3)	5159(4)	58.7(9)
C20	2092(3)	5449(4)	4857(3)	58.6(10)
C22	-4618(3)	4314(3)	8662(3)	49.4(8)
C24	-4148(3)	2127(3)	9418(3)	41.7(7)
C25	-2417(2)	3589(3)	8493(2)	28.3(5)
C27	1110(3)	141(3)	8378(3)	45.6(7)
C29	3760(3)	6648(3)	5455(3)	44.4(7)
C32	-2600(3)	9304(3)	8183(3)	51.5(8)
C33	336(3)	1159(3)	8305(3)	38.5(6)
C36	-5022(3)	3099(3)	9221(3)	45.3(7)
C2	-1265(3)	6757(3)	9269(3)	45.4(7)
C4	2184(3)	2455(3)	7857(2)	31.6(5)
C1	52(3)	3437(2)	7941(2)	28.2(5)
C3	-2860(3)	2362(3)	9064(3)	35.1(6)
C10	-3327(3)	4561(3)	8303(3)	39.3(6)
C12	-1732(3)	8976(3)	6201(3)	38.0(6)
C6	-1076(3)	3877(2)	8096(2)	27.9(5)
C15	-684(4)	7921(3)	4824(3)	47.9(8)
C7	-790(2)	7047(2)	7095(2)	29.8(5)
C9	1971(2)	6014(2)	6710(2)	28.1(5)
C21	2435(3)	261(3)	8201(3)	41.2(7)

C23	3877(3)	7029(3)	6405(3)	41.7(7)
C26	-1899(3)	8647(3)	7433(3)	36.4(6)
C28	2969(3)	1421(3)	7950(3)	38.0(6)

Table S10. Anisotropic Displacement Parameters (Å^2×10^3) for 6. The Anisotropic displacement factor exponent takes the form: -2π^2[h^{2}a*^{2}U$_{11}$+2hka*b*U$_{12}$+…].

Atom	U_{11}	U_{22}	U_{33}	U_{23}	U_{13}	U_{12}
Pd1	27.63(10)	27.43(10)	22.59(10)	-10.54(7)	-2.76(6)	-1.46(7)
N2	30.2(11)	49.9(14)	26.9(11)	-12.1(10)	-0.1(9)	-4.3(10)
N4	34.0(11)	30.1(11)	31.1(11)	-13.5(9)	-3.8(9)	0.4(9)
C5	29.9(12)	30.2(12)	22.7(11)	-10.7(10)	-2.8(9)	1.9(10)
C8	44.1(18)	47.8(19)	87(3)	-11.5(19)	-14.0(19)	-15.3(16)
N9	34.2(11)	27.8(11)	34.7(12)	-9.7(9)	-9.2(9)	-1.4(9)
N12	39.0(12)	30.4(11)	30.5(11)	-8.7(9)	-6.3(9)	-1.4(10)
C13	46.1(17)	51.4(18)	58.3(19)	-37.7(16)	-11.7(15)	-0.8(14)
C16	44(2)	110(4)	50(2)	-6(2)	17.2(17)	-13(2)
C17	66(2)	38.4(17)	62(2)	-4.9(16)	-19.8(18)	5.9(16)
C20	44.7(18)	104(3)	43.2(18)	-45(2)	-1.5(14)	-7.3(19)
C22	31.0(15)	54.4(19)	55.1(19)	-11.9(15)	-1.5(13)	11.3(14)
C24	36.5(15)	44.0(16)	42.8(16)	-13.1(13)	2.9(12)	-13.9(13)
C25	28.2(12)	34.4(13)	23.3(12)	-11.3(10)	-3.8(9)	-0.6(10)
C27	47.3(17)	29.8(14)	56.8(19)	-13.6(13)	9.9(14)	-4.2(13)

C29	31.4(14)	51.3(18)	38.7(16)	-2.3(13)	1.2(12)	-2.5(13)
C32	51.6(19)	40.7(17)	72(2)	-32.5(17)	1.8(17)	2.0(15)
C33	34.0(14)	33.6(14)	47.9(16)	-15.5(12)	5.9(12)	-4.4(11)
C36	26.2(13)	63(2)	43.0(16)	-14.7(15)	-1.0(12)	-4.8(13)
C2	58.8(19)	47.2(17)	30.2(14)	-15.3(13)	0.7(13)	9.1(15)
C4	30.0(13)	34.1(13)	31.2(13)	-12.0(11)	-3.1(10)	-2.4(11)
C1	31.9(13)	30.3(12)	24.7(12)	-12.1(10)	-3.9(10)	-4.8(10)
C3	33.4(14)	36.9(14)	35.0(14)	-13.4(12)	0.2(11)	-1.1(11)
C10	34.9(14)	37.6(15)	39.4(15)	-7.5(12)	-0.3(12)	3.2(12)
C12	37.0(14)	28.0(13)	47.1(16)	-10.1(12)	-10.9(12)	-2.5(11)
C6	35.0(13)	26.7(12)	22.4(11)	-8.9(9)	-4.5(10)	-0.1(10)
C15	62(2)	51.3(18)	27.8(14)	-10.4(13)	-9.0(13)	1.0(16)
C7	29.5(12)	29.6(12)	30.9(13)	-11.2(10)	-4.9(10)	-2.6(10)
C9	26.6(12)	29.1(12)	27.7(12)	-8.8(10)	-4.4(9)	-1.8(10)
C21	45.5(16)	34.2(14)	40.6(16)	-10.6(12)	0.2(13)	9.3(13)
C23	32.7(14)	31.6(14)	51.8(18)	-3.1(13)	-8.3(13)	-4.2(11)
C26	32.4(13)	31.7(13)	48.4(16)	-18.0(12)	-4.8(12)	-0.7(11)
C28	30.1(13)	43.8(16)	39.0(15)	-14.2(13)	-1.3(11)	3.5(12)

Table S11. Bond lengths for 6

Atom	Atom	Length/Å	Atom	Atom	Length/Å
Pd1	C1	2.033(3)	N12	C7	1.355(3)

Pd1	C6	2.029(3)	C16	C29	1.495(5)
Pd1	C7	2.083(3)	C17	C12	1.487(4)
Pd1	C9	2.079(3)	C22	C36	1.375(5)
N2	C20	1.455(4)	C22	C10	1.391(4)
N2	C29	1.391(4)	C24	C36	1.373(5)
N2	C9	1.352(3)	C24	C3	1.386(4)
N4	C2	1.454(4)	C25	C3	1.401(4)
N4	C7	1.357(3)	C25	C10	1.397(4)
N4	C26	1.389(4)	C25	C6	1.455(4)
C5	C33	1.402(4)	C27	C33	1.373(4)
C5	C4	1.397(4)	C27	C21	1.385(4)
C5	C1	1.442(4)	C29	C23	1.344(5)
C8	C23	1.494(4)	C32	C26	1.498(4)
N9	C13	1.449(4)	C4	C28	1.390(4)
N9	C9	1.356(3)	C1	C6	1.290(4)
N9	C23	1.393(4)	C12	C26	1.351(4)
N12	C12	1.397(4)	C21	C28	1.383(4)
N12	C15	1.453(4)			

Table S12. Bond angles for 6

Atom	Atom	Atom	Angle/°	Atom	Atom	Atom	Angle/°
C1	Pd1	C7	148.92(10)	C23	C29	C16	130.8(3)

C1	Pd1	C9	113.15(10)	C27	C33	C5	121.8(3)
C6	Pd1	C1	37.04(11)	C24	C36	C22	119.8(3)
C6	Pd1	C7	111.88(10)	C28	C4	C5	121.4(3)
C6	Pd1	C9	150.19(10)	C5	C1	Pd1	141.2(2)
C9	Pd1	C7	97.91(10)	C6	C1	Pd1	71.30(16)
C29	N2	C20	124.9(3)	C6	C1	C5	147.5(3)
C9	N2	C20	123.3(2)	C24	C3	C25	121.2(3)
C9	N2	C29	111.9(2)	C22	C10	C25	121.1(3)
C7	N4	C2	122.4(2)	N12	C12	C17	122.3(3)
C7	N4	C26	112.0(2)	C26	C12	N12	105.8(2)
C26	N4	C2	125.5(2)	C26	C12	C17	131.9(3)
C33	C5	C1	122.8(2)	C25	C6	Pd1	142.0(2)
C4	C5	C33	116.8(2)	C1	C6	Pd1	71.66(16)
C4	C5	C1	120.3(2)	C1	C6	C25	146.3(3)
C9	N9	C13	122.0(2)	N4	C7	Pd1	127.75(19)
C9	N9	C23	111.8(2)	N12	C7	Pd1	128.8(2)
C23	N9	C13	126.1(3)	N12	C7	N4	103.4(2)
C12	N12	C15	124.8(2)	N2	C9	Pd1	127.66(19)
C7	N12	C12	112.1(2)	N2	C9	N9	103.6(2)
C7	N12	C15	123.0(3)	N9	C9	Pd1	128.6(2)
C36	C22	C10	120.3(3)	C28	C21	C27	119.0(3)
C36	C24	C3	120.4(3)	N9	C23	C8	122.8(3)

C3	C25	C6	122.9(2)	C29	C23	C8	130.9(3)
C10	C25	C3	117.2(2)	C29	C23	N9	106.2(3)
C10	C25	C6	119.8(2)	N4	C26	C32	122.6(3)
C33	C27	C21	120.6(3)	C12	C26	N4	106.6(3)
N2	C29	C16	122.6(3)	C12	C26	C32	130.7(3)
C23	C29	N2	106.5(3)	C21	C28	C4	120.4(3)

Table S13. Torsion angles for 6

A	B	C	D	Angle/°	A	B	C	D	Angle/°
Pd1	C1	C6	C25	178.5(4)	C4	C5	C1	Pd1	10.8(4)
N2	C29	C23	C8	180.0(3)	C4	C5	C1	C6	-170.9(4)
N2	C29	C23	N9	-0.5(3)	C1	C5	C33	C27	179.1(3)
C5	C4	C28	C21	1.5(4)	C1	C5	C4	C28	179.9(2)
C5	C1	C6	Pd1	-178.9(4)	C3	C24	C36	C22	0.4(5)
C5	C1	C6	C25	-0.4(7)	C3	C25	C10	C22	-0.3(4)
N12	C12	C26	N4	-0.1(3)	C3	C25	C6	Pd1	-174.3(2)
N12	C12	C26	C32	-177.4(3)	C3	C25	C6	C1	8.0(5)
C13	N9	C9	Pd1	4.6(4)	C10	C22	C36	C24	-0.4(5)
C13	N9	C9	N2	-179.9(2)	C10	C25	C3	C24	0.3(4)
C13	N9	C23	C8	0.0(5)	C10	C25	C6	Pd1	5.9(4)
C13	N9	C23	C29	-179.7(3)	C10	C25	C6	C1	-171.8(4)
C16	C29	C23	C8	-2.0(6)	C12	N12	C7	Pd1	178.10(19)

C16	C29	C23	N9	177.6(4)	C12	N12	C7	N4	0.0(3)
C17	C12	C26	N4	177.8(3)	C6	C25	C3	C24	-179.4(3)
C17	C12	C26	C32	0.6(6)	C6	C25	C10	C22	179.4(3)
C20	N2	C29	C16	2.1(5)	C15	N12	C12	C17	0.1(5)
C20	N2	C29	C23	-179.7(3)	C15	N12	C12	C26	178.4(3)
C20	N2	C9	Pd1	-4.7(4)	C15	N12	C7	Pd1	-0.2(4)
C20	N2	C9	N9	179.7(3)	C15	N12	C7	N4	-178.3(3)
C27	C21	C28	C4	-1.1(4)	C7	N4	C26	C32	177.6(3)
C29	N2	C9	Pd1	174.9(2)	C7	N4	C26	C12	0.2(3)
C29	N2	C9	N9	-0.7(3)	C7	N12	C12	C17	-178.1(3)
C33	C5	C4	C28	-0.8(4)	C7	N12	C12	C26	0.1(3)
C33	C5	C1	Pd1	-168.5(2)	C9	N2	C29	C16	-177.5(3)
C33	C5	C1	C6	9.9(6)	C9	N2	C29	C23	0.8(4)
C33	C27	C21	C28	0.0(5)	C9	N9	C23	C8	179.7(3)
C36	C22	C10	C25	0.3(5)	C9	N9	C23	C29	0.0(3)
C36	C24	C3	C25	-0.4(5)	C21	C27	C33	C5	0.6(5)
C2	N4	C7	Pd1	2.3(4)	C23	N9	C9	Pd1	-175.12(19)
C2	N4	C7	N12	-179.6(3)	C23	N9	C9	N2	0.4(3)
C2	N4	C26	C32	-2.8(4)	C26	N4	C7	Pd1	-178.23(19)
C2	N4	C26	C12	179.7(3)	C26	N4	C7	N12	-0.1(3)
C4	C5	C33	C27	-0.2(4)					

Table S14. Hydrogen Atom Coordinates (Å×10^4) and Isotropic Displacement Parameters (Å^2×10^3) for 6

Atom	*x*	*y*	*z*	U(eq)
H8A	4577	8560	6652	94
H8B	5636	7858	6078	94
H8C	5222	7271	7510	94
H13A	2245	7723	8115	70
H13B	3221	6612	8817	70
H13C	1740	6319	8737	70
H16A	4840	6023	4277	112
H16B	5402	7257	4391	112
H16C	4161	7356	3601	112
H17A	-1415	10490	4630	88
H17B	-2590	10698	5481	88
H17C	-2770	9844	4681	88
H20A	1873	6104	4075	88
H20B	1317	4978	5250	88
H20C	2745	4880	4705	88
H22	-5222	4988	8521	59
H24	-4428	1288	9799	50
H27	733	-652	8551	55
H32A	-3400	8865	8545	77

H32B	-2804	10168	7654	77
H32C	-2054	9310	8840	77
H33	-569	1053	8437	46
H36	-5903	2933	9470	54
H2A	-867	5934	9397	68
H2B	-2140	6648	9645	68
H2C	-750	7207	9650	68
H4	2564	3249	7665	38
H3	-2266	1680	9210	42
H10	-3059	5404	7922	47
H15A	-1461	7923	4383	72
H15B	-181	7152	4936	72
H15C	-162	8649	4347	72
H21	2970	-444	8251	49
H28	3878	1512	7840	46

Crystal structure data for ***5***

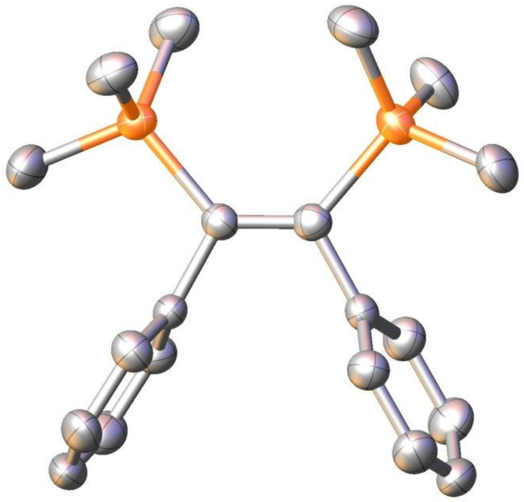

Table S15. Crystal data and structure refinement for 5

Identification code	cis-(Ph)(Me3Si)C=C(SiMe3)(Ph)
Empirical formula	$C_{20}H_{28}Si_2$
Formula weight	324.60
Temperature/K	173
Crystal system	monoclinic
Space group	I2/a
a/Å	14.9857(7)
b/Å	11.5692(5)
c/Å	22.8580(12)
α/°	90
β/°	91.020(5)
γ/°	90
Volume/Å^3	3962.3(3)
Z	8
ρ_{calc}g/cm^3	1.088
μ/mm^{-1}	1.566
F(000)	1408.0
Crystal size/mm^3	0.35 × 0.25 × 0.05
Radiation	CuKα (λ = 1.54184)
2Θ range for data collection/°	13.918 to 143.706
Index ranges	-13 ≤ h ≤ 18, -14 ≤ k ≤ 14, -20 ≤ l ≤ 28

Reflections collected	9565
Independent reflections	3639 [R_{int} = 0.0418, R_{sigma} = 0.0511]
Data/restraints/parameters	3639/0/206
Goodness-of-fit on F^2	1.061
Final R indexes [I>=2σ (I)]	R_1 = 0.0517, wR_2 = 0.1212
Final R indexes [all data]	R_1 = 0.0782, wR_2 = 0.1326
Largest diff. peak/hole / e $Å^{-3}$	0.37/-0.25

Table S16. Fractional Atomic Coordinates (×10^4) and Equivalent Isotropic Displacement Parameters ($Å^2$×10^3) for *5*. U_{eq} is defined as 1/3 of of the trace of the orthogonalised U_{IJ} tensor.

**Atom**	***x***	***y***	***z***	**U(eq)**
Si1	2930.3(4)	2136.2(5)	6207.3(3)	36.7(2)
Si2	1295.6(4)	4626.4(5)	6385.6(3)	39.9(2)
C3	1299.1(13)	913.8(17)	6215.4(9)	33.7(5)
C4	-1259.9(17)	2137.0(19)	6725.9(11)	51.1(7)
C5	-1738.5(16)	2343(2)	6215.5(12)	50.6(7)
C6	986.1(15)	-927(2)	6661.9(12)	53.7(7)
C7	-1309.2(17)	2757(2)	5735.4(12)	53.1(7)
C8	-353.6(15)	2350.7(18)	6752.3(10)	42.4(6)
C9	1044.3(14)	440.7(19)	5681.3(10)	43.0(6)
C10	90.9(14)	2768.0(16)	6269.6(9)	35.2(5)
C11	1269.7(15)	210.9(19)	6706.3(10)	43.1(6)

C12	-399.7(16)	2974.0(19)	5763.5(10)	45.2(6)
C13	1663.4(14)	2127.1(16)	6256.7(8)	32.5(5)
C14	3355.6(16)	3253(2)	5695.3(10)	52.9(6)
C15	2319.8(16)	4993(2)	6817.9(11)	54.7(7)
C16	769.5(15)	-693(2)	5638.6(12)	53.9(7)
C17	350.7(17)	5239(2)	6805.0(11)	54.8(7)
C18	1081.1(14)	3016.8(17)	6293.6(9)	34.5(5)
C19	741.4(15)	-1372(2)	6130.1(14)	56.7(7)
C20	3487.3(15)	2269(2)	6939.7(10)	51.0(7)
C21	1321.0(18)	5342.7(19)	5652.6(10)	55.6(7)
C22	3263.2(15)	712(2)	5905.0(12)	57.2(7)

Table S17. Anisotropic Displacement Parameters (Å^2×10^3) for 5. The Anisotropic displacement factor exponent takes the form: $-2\pi^2[h^2a^{*2}U_{11}+2hka^*b^*U_{12}+\ldots]$.

**Atom**	**U_{11}**	**U_{22}**	**U_{33}**	**U_{23}**	**U_{13}**	**U_{12}**
Si1	32.1(4)	36.2(4)	42.0(4)	2.5(2)	1.3(3)	-2.4(3)
Si2	45.9(4)	29.4(4)	44.4(4)	0.6(3)	0.3(3)	0.2(3)
C3	24.7(11)	31.1(12)	45.4(13)	1.3(10)	1.4(9)	1.3(10)
C4	44.8(16)	46.7(15)	62.1(17)	2.1(11)	10.8(13)	-0.8(12)
C5	30.8(13)	40.1(14)	81(2)	-5.2(13)	-1.3(14)	-0.1(11)
C6	46.5(15)	36.1(14)	78.6(19)	11.4(13)	7.5(14)	2.4(12)
C7	41.1(16)	56.7(17)	61.2(17)	1.5(13)	-11.3(13)	3.0(13)
C8	39.0(14)	39.5(13)	48.7(14)	3.4(10)	1.2(11)	-2.0(11)

C9	37.0(13)	42.2(14)	49.7(14)	-6.2(11)	-0.6(11)	-2.3(11)
C10	33.8(13)	25.9(11)	45.8(14)	-3.4(9)	1.5(11)	2(1)
C11	44.1(14)	35.9(13)	49.2(15)	0.8(10)	1.3(11)	0.4(11)
C12	41.3(15)	45.9(14)	48.4(14)	4.5(10)	-1.8(12)	1.9(11)
C13	34.0(12)	29.8(12)	33.6(12)	0.7(8)	0.0(9)	-0.7(10)
C14	44.8(14)	59.8(16)	54.4(15)	15.1(12)	4.7(12)	-3.5(13)
C15	60.1(17)	40.3(13)	63.3(17)	-3.4(12)	-6.6(13)	-10.6(13)
C16	41.4(15)	49.8(16)	70.4(18)	-21.7(14)	-2.0(13)	-0.9(13)
C17	61.0(16)	36.6(14)	66.9(17)	-8.9(11)	5.5(13)	4.6(12)
C18	34.1(12)	34.6(12)	34.8(12)	2.8(9)	-0.2(10)	-2.5(10)
C19	35.3(14)	31.2(14)	104(2)	-10.8(15)	6.5(15)	-2.0(11)
C20	40.1(14)	62.6(17)	50.4(15)	6.4(12)	0.3(12)	-6.3(12)
C21	80.1(19)	35.0(14)	51.7(15)	4.6(11)	0.3(13)	0.7(12)
C22	36.8(14)	53.1(15)	82.0(19)	-12.0(13)	9.0(13)	3.7(13)

Table S18. Bond Lengths for 5

Atom	Atom	Length/Å	Atom	Atom	Length/Å
Si1	C13	1.904(2)	C4	C8	1.381(3)
Si1	C14	1.863(2)	C5	C7	1.368(3)
Si1	C20	1.864(2)	C6	C11	1.386(3)
Si1	C22	1.859(2)	C6	C19	1.365(3)
Si2	C15	1.860(2)	C7	C12	1.386(3)

Si2	C17	1.864(2)	C8	C10	1.386(3)
Si2	C18	1.901(2)	C9	C16	1.378(3)
Si2	C21	1.870(2)	C10	C12	1.380(3)
C3	C9	1.385(3)	C10	C18	1.512(3)
C3	C11	1.387(3)	C13	C18	1.353(3)
C3	C13	1.509(3)	C16	C19	1.372(4)
C4	C5	1.380(3)			

Table S19. Bond Angles for 5

Atom	Atom	Atom	Angle/°	Atom	Atom	Atom	Angle/°
C14	Si1	C13	113.18(10)	C19	C6	C11	120.1(2)
C14	Si1	C20	110.75(11)	C5	C7	C12	120.1(2)
C20	Si1	C13	112.24(10)	C4	C8	C10	120.9(2)
C22	Si1	C13	106.96(10)	C16	C9	C3	121.1(2)
C22	Si1	C14	106.50(12)	C8	C10	C18	121.53(19)
C22	Si1	C20	106.75(12)	C12	C10	C8	118.1(2)
C15	Si2	C17	105.50(12)	C12	C10	C18	120.36(19)
C15	Si2	C18	114.75(10)	C6	C11	C3	120.8(2)
C15	Si2	C21	110.20(12)	C10	C12	C7	121.1(2)
C17	Si2	C18	107.51(10)	C3	C13	Si1	111.20(13)
C17	Si2	C21	108.66(12)	C18	C13	Si1	130.13(15)
C21	Si2	C18	109.95(10)	C18	C13	C3	118.60(18)

C9	C3	C11	118.0(2)	C19	C16	C9	120.0(2)
C9	C3	C13	121.09(19)	C10	C18	Si2	110.77(14)
C11	C3	C13	120.77(19)	C13	C18	Si2	130.10(16)
C5	C4	C8	120.1(2)	C13	C18	C10	119.12(18)
C7	C5	C4	119.6(2)	C6	C19	C16	120.1(2)

Table S20. Torsion Angles for 5

A	B	C	D	Angle/°	A	B	C	D	Angle/°
Si1	C13	C18	Si2	-6.7(3)	C9	C3	C11	C6	-0.5(3)
Si1	C13	C18	C10	174.19(15)	C9	C3	C13	Si1	-91.6(2)
C3	C9	C16	C19	0.3(3)	C9	C3	C13	C18	85.4(3)
C3	C13	C18	Si2	176.85(15)	C9	C16	C19	C6	-0.1(4)
C3	C13	C18	C10	-2.2(3)	C11	C3	C9	C16	0.0(3)
C4	C5	C7	C12	-0.4(4)	C11	C3	C13	Si1	83.7(2)
C4	C8	C10	C12	0.6(3)	C11	C3	C13	C18	-99.2(2)
C4	C8	C10	C18	179.41(19)	C11	C6	C19	C16	-0.5(4)
C5	C4	C8	C10	-0.3(3)	C12	C10	C18	Si2	77.9(2)
C5	C7	C12	C10	0.7(4)	C12	C10	C18	C13	-102.8(2)
C8	C4	C5	C7	0.2(4)	C13	C3	C9	C16	175.5(2)
C8	C10	C12	C7	-0.8(3)	C13	C3	C11	C6	-176.03(19)
C8	C10	C18	Si2	-100.89(19)	C18	C10	C12	C7	-179.63(19)
C8	C10	C18	C13	78.4(3)	C19	C6	C11	C3	0.8(3)

Table S21. Hydrogen Atom Coordinates (Å×10^4) and Isotropic Displacement Parameters (Å^2×10^3) for 5

Atom	*x*	*y*	*z*	U(eq)
H4	-1555	1847	7060	61
H5	-2363	2199	6197	61
H6	962	-1397	7002	64
H7	-1635	2895	5382	64
H8	-30	2210	7106	51
H9	1059	906	5339	52
H11	1446	513	7077	52
H12	-109	3270	5429	54
H14A	3144	3079	5297	79
H14B	4010	3251	5708	79
H14C	3137	4015	5812	79
H15A	2353	5832	6873	82
H15B	2847	4727	6608	82
H15C	2299	4613	7200	82
H16	599	-1005	5269	65
H17A	305	4834	7180	82
H17B	-206	5142	6579	82
H17C	456	6064	6876	82
H19	551	-2154	6100	68

H20A	3364	3034	7104	77
H20B	4133	2172	6899	77
H20C	3258	1671	7201	77
H21A	1421	6174	5704	83
H21B	750	5217	5447	83
H21C	1805	5012	5423	83
H22A	3107	99	6181	86
H22B	3909	704	5844	86
H22C	2950	581	5531	86

*Crystal Structure Data for **10***

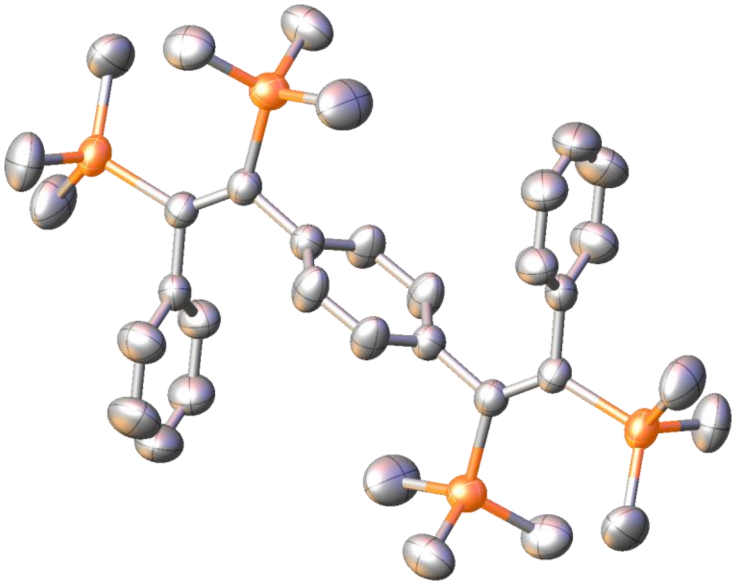

Table 22. Crystal data and structure refinement data for 10

Identification code	C34H50Si4
Empirical formula	$C_{22.67}H_{33.33}Si_{2.67}$

Formula weight	380.75
Temperature/K	298
Crystal system	monoclinic
Space group	C2/c
a/Å	15.2223(6)
b/Å	11.1780(4)
c/Å	21.3358(8)
α/°	90
β/°	95.192(3)
γ/°	90
Volume/Å^3	3615.5(2)
Z	6
ρ_{calc}g/cm^3	1.0491
μ/mm^{-1}	0.184
F(000)	1241.6
Crystal size/mm^3	0.25 × 0.2 × 0.1
Radiation	Mo Kα (λ = 0.71073)
2Θ range for data collection/°	7.12 to 58.34
Index ranges	$-17 \le h \le 11$, $-15 \le k \le 7$, $-26 \le l \le 28$
Reflections collected	5931
Independent reflections	3811 [R_{int} = 0.0247, R_{sigma} = 0.0487]
Data/restraints/parameters	3811/0/177

Goodness-of-fit on F^2	0.989
Final R indexes [I>=2σ (I)]	R_1 = 0.0517, wR_2 = 0.1383
Final R indexes [all data]	R_1 = 0.0874, wR_2 = 0.1733
Largest diff. peak/hole / e Å^{-3}	0.32/-0.27

Table 23. Fractional Atomic Coordinates (×10^4) and Equivalent Isotropic Displacement Parameters (Å^2×10^3) for *10*. U_{eq} is defined as 1/3 of of the trace of the orthogonalised U_{IJ} tensor.

Atom	*x*	*y*	*z*	U(eq)
Si01	7193.4(5)	3618.5(6)	3124.0(3)	54.5(3)
Si02	5691.0(4)	1987.5(6)	4172.7(4)	54.2(2)
C003	5455.7(15)	4363(2)	4560.8(11)	44.6(5)
C004	6467.3(16)	5570(2)	3734.4(11)	49.7(6)
C005	6456.2(15)	4222(2)	3721.8(11)	45.8(5)
C006	5940.6(15)	3642(2)	4105.3(11)	45.3(5)
C007	4636.2(17)	4849(3)	4396.9(12)	60.0(7)
C008	5808.8(17)	4526(2)	5170.0(12)	57.6(7)
C009	7060.4(19)	6202(2)	4130.7(14)	63.4(7)
C00A	5885(2)	6210(3)	3325.7(16)	74.4(9)
C00B	7753(2)	2181(3)	3342.2(17)	82.4(10)
C00C	7074(2)	7440(3)	4130.1(16)	78.2(9)
C00D	6497(3)	8053(3)	3728.4(19)	92.0(11)
C00E	6566(2)	1228(3)	4690.5(17)	81.6(10)

C00F	5499(2)	1244(3)	3388.6(16)	77.1(9)
C00G	8114(2)	4703(3)	3064.9(18)	90.8(11)
C00H	5904(3)	7453(3)	3328(2)	97.8(12)
C00I	6545(2)	3528(4)	2347.2(15)	88.7(10)
C00J	4637(2)	1816(3)	4539(2)	104.6(13)

Table 24. Anisotropic Displacement Parameters (Å^2×10^3) for 10. The Anisotropic displacement factor exponent takes the form: $-2\pi^2[h^2a^{*2}U_{11}+2hka^*b^*U_{12}+\ldots]$.

**Atom**	**U_{11}**	**U_{22}**	**U_{33}**	**U_{12}**	**U_{13}**	**U_{23}**
Si01	58.7(5)	58.0(4)	49.2(4)	-1.6(3)	18.5(3)	-5.3(3)
Si02	52.7(5)	47.3(4)	63.8(5)	-2.2(3)	12.3(3)	1.2(3)
C003	43.8(13)	46.1(12)	45.0(13)	2.5(10)	9.6(10)	1.7(10)
C004	59.1(15)	49.4(13)	42.4(13)	5.0(11)	14.2(11)	4.7(11)
C005	49.3(14)	46.3(12)	42.7(13)	0.6(10)	8.7(10)	-3.4(10)
C006	44.8(13)	48.4(12)	43.2(13)	2.5(10)	7.3(10)	-0.2(10)
C007	54.5(16)	83.5(18)	41.2(13)	16.9(13)	-0.3(11)	-5.6(13)
C008	45.1(14)	76.6(17)	51.1(15)	22.1(12)	3.5(11)	-0.5(13)
C009	79.7(19)	51.1(14)	59.1(17)	-0.8(13)	4.3(15)	-2.3(13)
C00A	83(2)	64.3(17)	74(2)	8.6(15)	-3.2(17)	8.9(16)
C00B	77(2)	79(2)	96(3)	18.3(16)	32.2(18)	3.0(18)
C00C	103(2)	55.0(16)	78(2)	-6.1(17)	16.6(19)	-6.7(16)
C00D	134(3)	49.4(17)	98(3)	4.6(19)	37(3)	6.1(18)
C00E	91(2)	68.0(19)	83(2)	-2.5(15)	-9.3(18)	15.0(17)

C00F	81(2)	65.5(18)	82(2)	-6.2(15)	-5.4(17)	-10.0(16)
C00G	83(2)	94(2)	103(3)	-21.6(18)	50(2)	-17(2)
C00H	128(3)	71(2)	92(3)	31(2)	2(2)	23(2)
C00I	92(3)	122(3)	54.4(18)	0(2)	14.9(17)	-8.7(19)
C00J	94(3)	79(2)	150(4)	-21.7(19)	59(3)	-2(2)

Table 25. Bond Lengths for 10

Atom	Atom	Length/Å	Atom	Atom	Length/Å
Si01	C005	1.897(2)	C003	C008	1.373(3)
Si01	C00B	1.859(3)	C004	C005	1.508(3)
Si01	C00G	1.867(3)	C004	C009	1.375(4)
Si01	C00I	1.854(3)	C004	C00A	1.384(4)
Si02	C006	1.896(2)	C005	C006	1.350(3)
Si02	C00E	1.857(3)	C007	C008[1]	1.384(3)
Si02	C00F	1.867(3)	C009	C00C	1.384(4)
Si02	C00J	1.856(3)	C00A	C00H	1.390(5)
C003	C006	1.506(3)	C00C	C00D	1.356(5)
C003	C007	1.377(3)	C00D	C00H	1.363(5)

[1]1-X,1-Y,1-Z

Table 26. Bond Angles for 10

Atom	Atom	Atom	Angle/°	Atom	Atom	Atom	Angle/°
C00B	Si01	C005	115.13(13)	C00A	C004	C005	120.0(2)
C00G	Si01	C005	107.93(13)	C00A	C004	C009	118.0(2)
C00G	Si01	C00B	104.39(17)	C004	C005	Si01	111.16(15)
C00I	Si01	C005	108.72(14)	C006	C005	Si01	130.43(17)
C00I	Si01	C00B	111.82(17)	C006	C005	C004	118.4(2)
C00I	Si01	C00G	108.53(17)	C003	C006	Si02	111.06(16)
C00E	Si02	C006	110.70(13)	C005	C006	Si02	130.23(17)
C00F	Si02	C006	112.46(13)	C005	C006	C003	118.7(2)
C00F	Si02	C00E	112.03(15)	C008[1]	C007	C003	121.1(2)
C00J	Si02	C006	108.62(14)	C007[1]	C008	C003	121.7(2)
C00J	Si02	C00E	107.63(19)	C00C	C009	C004	121.5(3)
C00J	Si02	C00F	105.07(18)	C00H	C00A	C004	120.2(3)
C007	C003	C006	122.2(2)	C00D	C00C	C009	119.8(3)
C008	C003	C006	120.6(2)	C00H	C00D	C00C	120.1(3)
C008	C003	C007	117.1(2)	C00D	C00H	C00A	120.4(3)
C009	C004	C005	122.0(2)				

[1]1-X,1-Y,1-Z

Table 27. Hydrogen Atom Coordinates (Å×10⁴) and Isotropic Displacement Parameters (Å²×10³) for 10

Atom	*x*	*y*	*z*	U(eq)
H00A	5481(2)	5806(3)	3048.9(16)	89.3(10)
H00p	5508(3)	7878(3)	3054(2)	117.4(14)
H00f	6507(3)	8885(3)	3726.3(19)	110.5(14)
H00e	7477(2)	7850(3)	4404.4(16)	93.9(11)
H009	7461.4(19)	5788(2)	4405.3(14)	76.1(9)
H007	4377.1(17)	4755(3)	3988.1(12)	72.0(8)
H008	6361.5(17)	4207(2)	5294.9(12)	69.2(8)
H00b	8115(13)	2278(7)	3731(6)	123.6(14)
H00c	7319(2)	1574(6)	3391(12)	123.6(14)
H00d	8115(13)	1948(11)	3017(6)	123.6(14)
H00g	7119(4)	1297(18)	4511(6)	122.5(14)
H00h	6612(11)	1595(14)	5099(4)	122.5(14)
H00i	6418(8)	397(5)	4730(9)	122.5(14)
H00j	5086(12)	1706(11)	3122(4)	115.6(13)
H00k	6047(3)	1189(18)	3200(5)	115.6(13)
H00l	5267(14)	456(8)	3441.0(19)	115.6(13)
H00m	7876(2)	5481(6)	2960(12)	136.2(17)
H00n	8467(10)	4743(18)	3461(4)	136.2(17)
H00o	8474(10)	4445(13)	2743(9)	136.2(17)

H00q	6068(10)	2973(18)	2371(4)	133.1(16)
H00r	6312(15)	4303(6)	2232(6)	133.1(16)
H00s	6919(5)	3260(20)	2036(3)	133.1(16)
H00t	4714(6)	2080(30)	4967(5)	157(2)
H00u	4188(5)	2290(20)	4310(9)	157(2)
H00v	4465(11)	990(5)	4528(14)	157(2)

Crystal Structure Data for ***11***

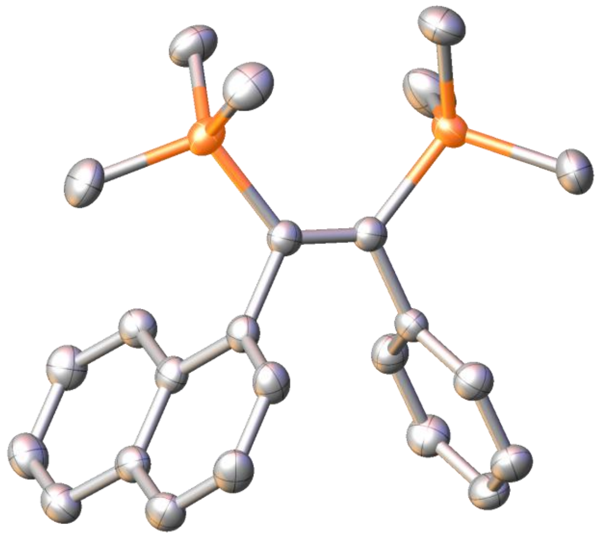

Table 28. Crystal data and structure refinement data for 11

Identification code	C24H30Si2
Empirical formula	$C_{19.2}H_{24}Si_{1.6}$
Formula weight	299.74
Temperature/K	173.15
Crystal system	monoclinic

Space group	$P2_1/c$
a/Å	11.5212(3)
b/Å	15.2445(5)
c/Å	12.6733(3)
α/°	90
β/°	90.765(2)
γ/°	90
Volume/$Å^3$	2225.68(11)
Z	5
ρ_{calc}g/cm^3	1.1181
μ/mm^{-1}	0.164
F(000)	808.9
Crystal size/mm^3	0.25 × 0.3 × 0.3
Radiation	Mo Kα (λ = 0.71073)
2Θ range for data collection/°	6.96 to 58.12
Index ranges	-14 ≤ h ≤ 11, -17 ≤ k ≤ 20, -13 ≤ l ≤ 16
Reflections collected	13843
Independent reflections	5107 [R_{int} = 0.0346, R_{sigma} = 0.0413]
Data/restraints/parameters	5107/0/240
Goodness-of-fit on F^2	0.963
Final R indexes [I>=2σ (I)]	R_1 = 0.0429, wR_2 = 0.1274
Final R indexes [all data]	R_1 = 0.0569, wR_2 = 0.1455

Largest diff. peak/hole / e Å^{-3} 0.40/-0.31

Table 29. Fractional Atomic Coordinates (×10^4) and Equivalent Isotropic Displacement Parameters (Å^2×10^3) for *11*. U_{eq} is defined as 1/3 of of the trace of the orthogonalised U_{IJ} tensor.

Atom	*x*	*y*	*z*	U(eq)
Si01	7034.1(4)	7795.1(3)	1991.7(3)	30.95(15)
Si02	9508.1(4)	6154.5(3)	1809.3(3)	31.53(15)
C003	7734.3(13)	4471.9(11)	2912.7(11)	27.3(3)
C004	5833.8(13)	6224.1(10)	2367.3(12)	27.1(3)
C005	7027.8(13)	6550.1(10)	2085.4(11)	26.5(3)
C006	7579.8(13)	4997.0(11)	1986.3(11)	27.2(3)
C007	7461.3(13)	3567.4(11)	2874.1(12)	29.7(3)
C008	7890.1(13)	5954.7(11)	1977.1(11)	27.0(3)
C009	8167.1(14)	4831.5(11)	3879.1(11)	31.0(4)
C00A	4989.8(14)	6022.4(11)	1604.4(12)	32.5(4)
C00B	5543.2(14)	6135.3(11)	3421.8(12)	32.3(4)
C00C	7170.4(14)	4601.5(11)	1082.4(12)	33.6(4)
C00D	7028.9(14)	3196.9(12)	1921.3(12)	35.0(4)
C00E	3624.2(15)	5651.9(12)	2953.8(13)	36.9(4)
C00F	6889.4(15)	3709.1(12)	1044.8(13)	36.3(4)
C00G	3898.6(15)	5739.6(12)	1898.5(13)	36.7(4)
C00H	7639.4(15)	3051.6(12)	3787.5(13)	36.3(4)

C00I	4449.5(15)	5854.7(12)	3714.0(13)	37.4(4)
C00J	8347.5(15)	4303.7(12)	4738.2(12)	36.1(4)
C00K	8079.8(16)	3406.7(13)	4695.1(13)	39.7(4)
C00L	10051.9(16)	6970.1(14)	2800.2(14)	44.8(5)
C00M	8149.8(17)	8283.5(13)	1114.3(15)	47.1(5)
C00N	10308.0(16)	5110.5(14)	2091.1(14)	44.3(5)
C00O	5619.4(16)	8132.8(13)	1389.0(15)	46.2(5)
C00P	9842.7(17)	6443.1(15)	417.8(13)	47.8(5)
C00Q	7143(2)	8257.0(14)	3346.0(14)	53.7(5)

Table 30. Anisotropic Displacement Parameters (Å^2×10^3) for 11. The Anisotropic displacement factor exponent takes the form: -2π^2[h^{2}a*^{2}U$_{11}$+2hka*b*U$_{12}$+…].

Atom	U_{11}	U_{22}	U_{33}	U_{12}	U_{13}	U_{23}
Si01	36.1(3)	23.9(3)	32.8(3)	-1.50(17)	-3.54(18)	2.63(17)
Si02	26.6(3)	39.3(3)	28.5(3)	-2.19(18)	-2.05(17)	-0.49(18)
C003	23.6(7)	29.5(9)	28.7(8)	3.4(6)	0.8(5)	-1.9(6)
C004	27.6(8)	21.2(8)	32.3(8)	0.7(6)	-1.1(6)	-0.1(6)
C005	28.3(8)	27.4(8)	23.6(7)	-1.8(6)	-3.2(5)	1.8(6)
C006	26.1(8)	28.1(8)	27.4(7)	3.1(6)	-1.3(5)	-1.8(6)
C007	27.6(8)	27.3(9)	34.3(8)	1.8(6)	0.8(6)	-1.7(6)
C008	28.8(8)	28.8(9)	23.3(7)	-1.2(6)	-3.4(5)	1.0(6)
C009	34.2(8)	31.0(9)	27.9(8)	0.5(7)	-1.0(6)	-2.0(6)
C00A	35.2(9)	32.8(9)	29.4(8)	-2.2(7)	-2.5(6)	-1.5(6)

C00B	31.7(9)	34.4(9)	30.8(8)	0.7(7)	-3.9(6)	-0.6(7)
C00C	38.4(9)	32.9(9)	29.4(8)	2.8(7)	-7.1(6)	-2.5(7)
C00D	37.4(9)	27.1(9)	40.3(9)	0.2(7)	-1.9(7)	-5.9(7)
C00E	30.4(9)	34.9(10)	45.4(10)	-3.2(7)	3.2(6)	1.8(7)
C00F	39.7(10)	33.7(10)	35.4(9)	1.9(7)	-8.3(7)	-10.7(7)
C00G	34.4(9)	35.4(10)	40.1(9)	-5.4(7)	-5.0(7)	-3.0(7)
C00H	40.3(10)	31.1(9)	37.4(9)	1.0(7)	2.5(7)	3.7(7)
C00I	37.9(10)	41.6(10)	33.0(9)	-0.5(8)	5.4(6)	1.4(7)
C00J	39.4(9)	42.1(10)	26.8(8)	2.6(8)	-1.3(6)	-2.3(7)
C00K	45.4(10)	40.4(11)	33.4(9)	4.1(8)	0.7(7)	6.9(7)
C00L	38.4(10)	54.4(12)	41.3(10)	-11.6(9)	-8.5(7)	-2.6(8)
C00M	50.8(12)	35.4(11)	55.1(11)	-7.3(9)	0.8(8)	10.1(9)
C00N	31.0(9)	54.3(13)	47.5(10)	7.0(8)	-0.6(7)	-1.6(9)
C00O	43.6(11)	35.9(10)	58.9(12)	5.2(8)	-6.6(8)	10.1(9)
C00P	44.0(11)	64.6(14)	34.9(9)	-5.3(9)	4.4(7)	1.2(8)
C00Q	83.1(16)	33.9(11)	43.8(11)	-0.4(10)	-6.5(9)	-5.5(8)

Table 31. Bond Lengths for 11

**Atom**	**Atom**	**Length/Å**	**Atom**	**Atom**	**Length/Å**
Si01	C005	1.9017(17)	C005	C008	1.354(2)
Si01	C00M	1.8659(18)	C006	C008	1.503(2)
Si01	C00O	1.8633(18)	C006	C00C	1.373(2)

Si01	C00Q	1.8579(18)	C007	C00D	1.418(2)
Si02	C008	1.9036(16)	C007	C00H	1.412(2)
Si02	C00L	1.8693(18)	C009	C00J	1.367(2)
Si02	C00N	1.871(2)	C00A	C00G	1.385(2)
Si02	C00P	1.8629(17)	C00B	C00I	1.386(2)
C003	C006	1.430(2)	C00C	C00F	1.399(2)
C003	C007	1.415(2)	C00D	C00F	1.365(2)
C003	C009	1.426(2)	C00E	C00G	1.385(2)
C004	C005	1.510(2)	C00E	C00I	1.380(2)
C004	C00A	1.396(2)	C00H	C00K	1.363(2)
C004	C00B	1.389(2)	C00J	C00K	1.403(3)

Table 32. Bond Angles for 11

Atom	Atom	Atom	Angle/°	Atom	Atom	Atom	Angle/°
C00M	Si01	C005	116.02(8)	C008	C006	C003	121.54(13)
C00O	Si01	C005	107.30(8)	C00C	C006	C003	118.49(15)
C00O	Si01	C00M	104.55(9)	C00C	C006	C008	119.95(13)
C00Q	Si01	C005	108.72(8)	C00D	C007	C003	119.55(14)
C00Q	Si01	C00M	111.15(10)	C00H	C007	C003	118.98(14)
C00Q	Si01	C00O	108.77(10)	C00H	C007	C00D	121.46(16)
C00L	Si02	C008	110.57(8)	C005	C008	Si02	128.69(13)
C00N	Si02	C008	108.85(8)	C006	C008	Si02	112.93(11)

C00N	Si02	C00L	106.13(9)	C006	C008	C005	118.38(14)
C00P	Si02	C008	111.03(8)	C00J	C009	C003	120.36(16)
C00P	Si02	C00L	114.03(9)	C00G	C00A	C004	120.57(15)
C00P	Si02	C00N	105.90(9)	C00I	C00B	C004	121.27(15)
C007	C003	C006	119.46(13)	C00F	C00C	C006	122.16(15)
C009	C003	C006	121.89(15)	C00F	C00D	C007	120.09(16)
C009	C003	C007	118.64(13)	C00I	C00E	C00G	119.25(16)
C00A	C004	C005	122.47(14)	C00D	C00F	C00C	120.25(15)
C00B	C004	C005	119.47(13)	C00E	C00G	C00A	120.65(15)
C00B	C004	C00A	118.05(15)	C00K	C00H	C007	121.26(17)
C004	C005	Si01	110.33(10)	C00E	C00I	C00B	120.22(15)
C008	C005	Si01	131.24(12)	C00K	C00J	C009	120.77(15)
C008	C005	C004	118.39(14)	C00J	C00K	C00H	119.94(15)

Table 33. **Hydrogen Atom Coordinates (Å×10^4) and Isotropic Displacement Parameters (Å^2×10^3) for 11**

**Atom**	***x***	***y***	***z***	**U(eq)**
H00B	6093.2(14)	6266.6(11)	3941.9(12)	38.8(4)
H00I	4271.2(15)	5803.0(12)	4424.8(13)	44.9(5)
H00E	2891.8(15)	5458.4(12)	3147.9(13)	44.3(5)
H00G	3344.7(15)	5607.2(12)	1382.2(13)	44.0(5)
H00A	5161.9(14)	6078.4(11)	892.7(12)	39.0(4)

H00C	7076.6(14)	4937.8(11)	474.9(12)	40.4(4)
H00F	6606.7(15)	3463.9(12)	420.6(13)	43.6(5)
H00D	6839.9(14)	2604.2(12)	1893.0(12)	41.9(5)
H009	8327.6(14)	5428.2(11)	3925.5(11)	37.2(4)
H00J	8651.2(15)	4542.3(12)	5358.2(12)	43.4(5)
H00K	8202.8(16)	3054.5(13)	5285.3(13)	47.6(5)
H00H	7452.6(15)	2458.2(12)	3769.4(13)	43.5(5)
H00l	9727(9)	7536(2)	2644(6)	67.2(7)
H00m	9825(10)	6791(5)	3493.8(17)	67.2(7)
H00n	10883.1(18)	7002(7)	2772(8)	67.2(7)
H00o	8910.6(17)	8153(8)	1390(6)	70.6(7)
H00p	8067(8)	8041(7)	418(3)	70.6(7)
H00q	8046(8)	8907.8(16)	1083(9)	70.6(7)
H00r	10311(10)	4999(5)	2836.7(17)	66.4(7)
H00s	9931(7)	4634.2(19)	1729(8)	66.4(7)
H00t	11092(3)	5162(3)	1852(9)	66.4(7)
H00u	5530(5)	7864(7)	708(5)	69.3(7)
H00v	4997.8(17)	7949(8)	1835(5)	69.3(7)
H00w	5601(5)	8759.4(15)	1314(10)	69.3(7)
H00x	9465(10)	6035(6)	-49.8(14)	71.7(7)
H00y	9569(11)	7026(4)	269(4)	71.7(7)
H	10666(2)	6418(9)	317(3)	71.7(7)

H00z	6501(8)	8053(8)	3755(4)	80.5(8)
Ha	7858(7)	8071(9)	3673(5)	80.5(8)
Hb	7125(14)	8885.9(14)	3309.8(17)	80.5(8)

References

[1] a) L. R. Titcomb, PhD thesis, University of Sussex (UK), **2001**. b) D. E. Roberts, PhD thesis, University of Sussex (UK), **2013**.

[2] A. Simonneau, J. Friebel, M. Oestreich, *Eur. J. Org. Chem*. **2014**, 2077-2083.

[3] R. H. Blessing, *Acta Crystallogr, A, Found. Crystallogr*. **1995**, *51*, 33-38.

[4] L. Palatinus, G. Chapuis, *J. Appl. Cryst*. **2007**, *40*, 786-790.

[5] G. M. Sheldrick, *J. Appl. Cryst*. **2011**, *44*, 1281-1284.

[6] O. V. Dolomanov, L. J. Bourhis, R. J. Gildea, J. A. K. Howard, H. Puschmann, *J. Appl. Cryst*. **2009**, *42*, 339-341.
